# A game-based approach for designing a collaborative evolution mechanism for unmanned swarms on community networks

**DOI:** 10.1038/s41598-022-22365-z

**Published:** 2022-11-07

**Authors:** Zhonghong Wu, Li Pan, Minggang Yu, Jintao Liu, Dan Mei

**Affiliations:** 1 Weaponry Engineering College, Naval University of Engineering, Wuhan, China; 2 Electronics Engineering College, Naval University of Engineering, Wuhan, China; 3 Institute of Command and Control Engineering, Army Engineering University of PLA, Nanjing, China; 4 Qingdao Campus, Naval Aviation University, Qingdao, China

**Keywords:** Evolutionary theory, Applied mathematics, Information technology, Computational models

## Abstract

Intelligent and coordinated unmanned aerial vehicle (UAV) swarm combat will be the main mode of warfare in the future, and mechanistic design of autonomous cooperation within swarms is the key to enhancing combat effectiveness. Exploration of the essential features and patterns of autonomous collaboration in unmanned swarms has become the focus of scientific research and technological applications, in keeping with the evolving conceptions of the military theatre. However, given the unique attributes of the military and the novelty of the warfare mode of unmanned swarms, few achievements have been reported in the existing research. In this study, we analysed the military requirements of unmanned swarm operations and proposed an analytic framework for autonomous collaboration. Then, a literature review addressing swarm evolution dynamics, game-based swarm collaboration, and collaborative evolution on complex networks was conducted. Next, on the basis of the above work, we designed a community network for unmanned swarm cooperation and constructed a collaborative evolution model based on the multiplayer public goods game (PGG). Furthermore, according to the “network” and “model”, the dynamic evolution process of swarm collaboration was formally deduced. Finally, a simulation was conducted to analyse the influence of relevant parameters (i.e., swarm size, degree distribution, cost, multiplication factor) on the collaborative behaviour of unmanned swarms. According to the simulation results, some reasonable suggestions for collaborative management and control in swarm operation are given, which can provide theoretical reference and decision-making support for the design of coordination mechanisms and improved combat effectiveness in unmanned swarm operation.

## Introduction

With the maturity of concepts and technologies related to intelligent and unmanned combat, unmanned combat platforms and equipment have been applied with increasing frequency on training grounds and even on the battlefield due to their low cost, easy deployment, and flexible organization. Intelligent and collaborative unmanned aerial vehicle (UAV) swarms will be the main form of warfare in the future^1^.

Unmanned swarm combat is a joint combat operation in which multiple heterogeneous unmanned platforms expand the capability of a single platform to complete missions and the overall combat effectiveness of the swarms through capability complementation and coordinated actions^2^. Unmanned swarm combat places extremely high requirements on collaboration among multiple platforms. Currently, collaboration in unmanned swarms is mainly realized through centralized control and distributed autonomy. In the complex electromagnetic environment of the battlefield, poor communication and even communication failure are common^3^. When centralized control fails, the unmanned swarms must respond immediately according to the battlefield situation to achieve self-organization and self-coordination to continue carrying out the planned military actions.

**Figure 1:**
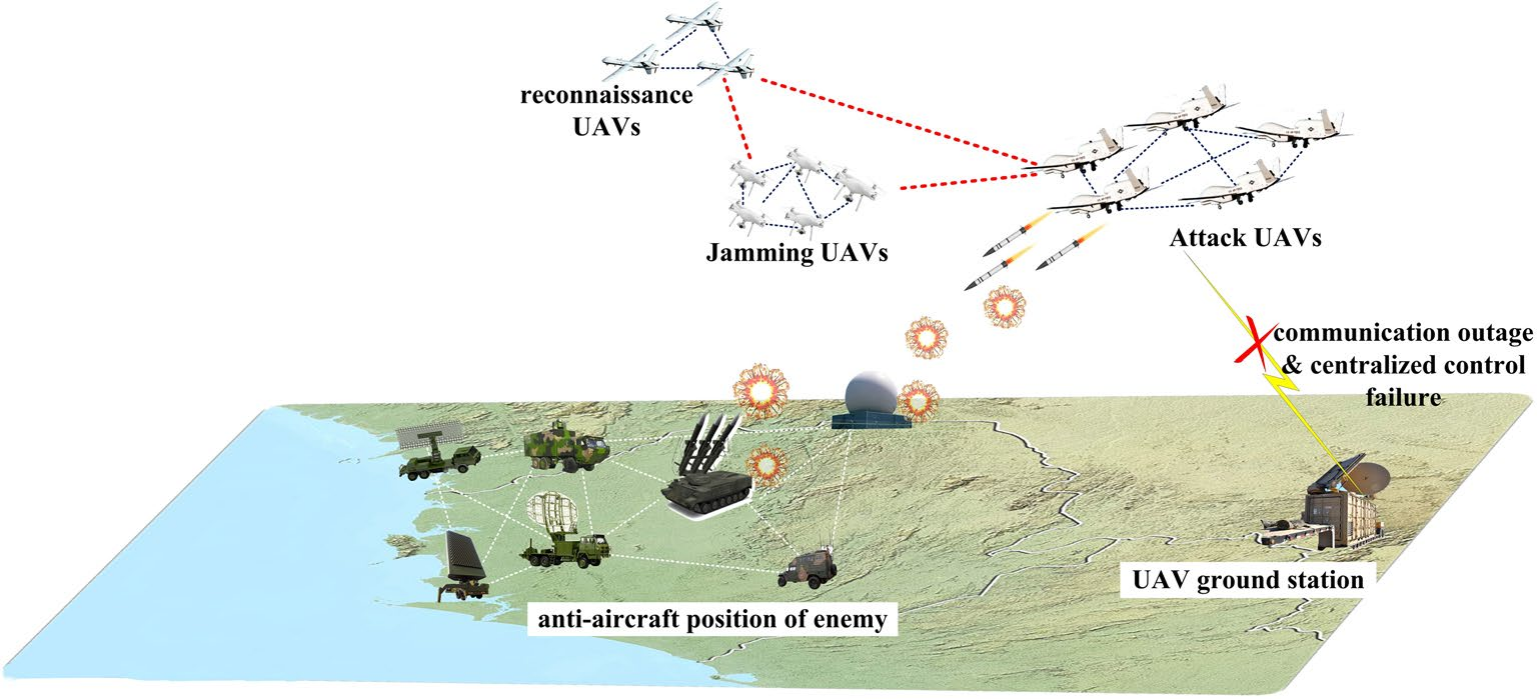
Autonomous collaboration of unmanned swarms.

The autonomous collaboration of unmanned swarms requires intelligent unmanned platforms with heterogeneous functions that carry out function segmentation and closely collaborate in various military missions based on an integrated air–space–ground information network. For example, in collaborative search tasks, unmanned swarms equipped with multiple sensors (e.g., visible light, infrared, radar) should be deployed to make full use of various detection methods to detect and locate obstructed targets and to improve area coverage through task division. In a collaborative strike mission, reconnaissance UAVs first implement an intelligence investigation to detect the position, speed, and technical and tactical indicators of the target. Then, jamming UAVs perform electronic interference on the target while attack UAVs attack the target. Figure 1 shows the scenario for autonomous collaboration of unmanned swarms in a strike mission.

Interaction and collaboration among multiple individuals is a common phenomenon in nature. In microbial communities^4,5^, the animal kingdom^6,7^, and human society^8,9^, individuals form a closely connected collaborative network through adaptation, communication, division of labour, and collaboration, thereby developing capabilities that a single individual does not have and accomplishing tasks that a single individual is unable to complete. Elucidation of the emergence and maintenance of collective collaborative behaviour has for a century been a problem that has puzzled researchers in many disciplines, including biology, computer science, and control science. Collaborative behaviour has become one of the frontier hot topics in multidisciplinary research.

In the military domain, exploring the characteristics and laws of swarm networking and probing into the autonomous organization and coordination mechanisms of swarms is of vital practical value for designing combat modes and developing the combat power of unmanned swarms.

However, given the unique attributes of the military and the novelty of the warfare mode of unmanned swarms, few achievements have been reported in the existing research. Much basic research work remains to be carried out in regard to the specific field of autonomous collaboration of unmanned swarms. First, community networks should be constructed that satisfy the needs of information communication, functional clustering, and network energy collection based on the combat features of unmanned swarms. Second, an evolutionary game model that can characterize autonomous collaboration in swarm operations must be determined. Third, dynamic processes of swarm collaborative evolution should be designed, including payoff calculation and strategy update rules.

In our previous work, we explored the collaboration and resource optimization allocation mechanism in unmanned swarms and deduced the strategy abundance function and conditions for strategy dominance in unmanned swarms based on evolutionary game theory^10–16^. Based on the previous achievements, the present study was carried out in three aspects. First, we designed a community information network based on the operational capabilities required of unmanned swarms. Second, we abstracted collaboration in unmanned swarms into an N-person evolutionary game with multiple rounds of iterations, established a collaborative evolution model of unmanned swarms based on the evolutionary game of multiple public goods, and presented the evolutionary dynamic process of unmanned swarms on complex community networks. Finally, through a simulated analysis of the influence of swarm size, degree distribution, cost, and payoff on the level of collaboration in unmanned swarms, we examined the mechanism of collaboration emergence in unmanned swarms and proposed reasonable suggestions for promoting collaboration in unmanned swarm combat.

Focusing on the military requirements of unmanned swarm operation, this paper innovatively introduces game theory and complex network theory into the design of the collaborative evolution mechanism of unmanned swarms. The proposed framework, model, and method provide a new view and technical approach to solve the autonomous collaboration of swarm combat.

## Analytical framework for autonomous collaborative behaviour in unmanned swarms

**Figure 2:**
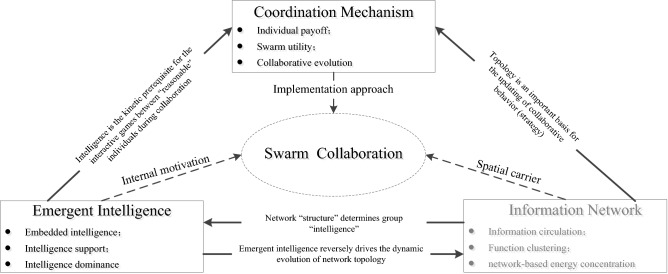
Framework for autonomous collaborative behaviour in unmanned swarms.

The autonomous collaboration and collaborative evolution of unmanned swarms involve three key components, i.e., the emergence of swarm intelligence, construction of information networks, and design of coordination mechanisms, which jointly form the basic framework of autonomous collaborative behaviour in unmanned swarms. Among these components, the emergence of intelligence from individuals to a swarm is the internal driving force of autonomous collaborative behaviour in unmanned swarms, information networks form the topological space where information interaction occurs within a swarm and serve as the spatial carrier of autonomous collaborative behaviour, and a coordination mechanism is the fundamental approach for achieving autonomous collaborative behaviour in unmanned swarms. Figure 2 shows the relationships among the three components.

According to the classic systems engineering principle “structure determines function”, an information network with a topological structure is the foundation for the emergence of intelligence in swarms, and the emergence of intelligence in turn drives the dynamic reconstruction of network topology. Moreover, the emergence of intelligence is essentially a time–space game among individuals based on payoff, with intelligence being the premise for “rational” individuals to collaborate to carry out interactive games. The adjustment of collaborative behaviour and the updating of strategy are closely correlated with the spatial network structure of a swarm. Therefore, network topology is an important basis for updating individual behaviour (strategy) in a coordination mechanism.

### Emergence of intelligence

Intelligence, including the single intelligence of individuals and the collective emergent intelligence of groups, is a key requirement of distributed autonomous control of swarms. In unmanned swarms, units with “intelligence” not only passively accept preset instructions but also, most importantly, optimally coordinate and organize their own resources, costs, behaviour, and other factors through the processes of unmanned autonomy, senior driven, collaborative interaction, utility optimization, capability generation, and so on. At the swarm level, higher-level intelligence beyond individual intelligence emerges, ultimately finally realizing optimization of the overall utility of the swarm. In fact, the concept of directing unmanned swarms to carry out military missions according to a predetermined plan has inherent shortcomings. In a complex environment, the battlefield situation is changing continuously. If micromanagement of a single unmanned platform is implemented, resources such as communication will be seriously overloaded. In other words, the responsive control of a large number of unmanned platforms is beyond the current technology, cognition, and decision-making capabilities of human beings, with a high probability of leading to the failure of combat operations. Hence, a greater degree of decision-making and permission to act must be transferred to the autonomous control system of an unmanned swarm to enable the unmanned platform to independently coordinate its own decision-making to elicit behaviours supporting the swarm's ability to realize its goals.

Moreover, intelligence and autonomy are the cores of intelligent combat mechanisms for winning in battlefields. The Defense Science Board of the U.S. Department of Defense pointed out that intelligence and autonomy are the core capabilities of the U.S. military’s unmanned systems and analysed the benefits brought by intelligence and autonomy to UAVs, unmanned ground systems, unmanned marine vehicles, and unmanned space systems^17^. In the future, unmanned swarm combat systems will achieve stronger perception, analysis, planning, decision-making, and execution capabilities to autonomously perceive battlefield situations, plan combat missions, carry out combat actions, coordinate combat actions, and evaluate combat effects.

Although the individual in the swarm has intelligence, the achievement of the optimal overall utility at the swarm level is not achieved overnight but, rather, is an iterative and self-organizing evolution process. Individuals must modify and improve strategies through a large number of repeated game processes, learning, imitation, and trial and error to constantly adapt to the external environment and finally achieve the optimal overall utility of the swarm. In the military field, from the long-term development of warfare, the generation of intelligence and its impact on combat is also a long-term development process. Based on the current situation, the development of unmanned combat originates from the remote-control approach featuring human–computer interaction, undergoes the collaborative transformation characterized by human–machine integration, and develops towards autonomous behaviour featuring human–machine integration^18^. It is foreseeable that the emergence of intelligence in unmanned swarms will also experience the evolution from “embedding of intelligence”, where humans play the leading role supplemented by machines, to “intelligent support” featuring unmanned autonomy, and finally to “intelligence dominance” with biomimetic autonomy and swarm attack and defence capabilities.

### Construction of an information network

From the perspective of scale, with air forces as the case considered, unmanned swarm combat in the future can be divided into three levels: the wing type formed by fewer than thirty or fifty UAVs, the cluster type formed by thirty to one hundred UAVs, and the swarm type formed by hundreds and even thousands of UAVs. From the perspective of intelligence, once unmanned swarms enter the stage of “intelligence dominance” with biomimetic autonomy and swarm attack and defence capabilities, the scale of swarms will certainly be extremely large. The expansion of scale leads to more complicated interactions. How to build a swarm network based on the demands of information interaction between nodes and the business logic of swarm combat is a practical problem that remains to be solved by combat planners.

The construction of information networks in unmanned swarm combat is not a rejection of traditional combat networks. Instead, these networks develop and evolve from traditional combat networks. Hence, a representative idea is to integrate random networks^19^ and community networks^20,21^ based on traditional tree networks to construct a network topology tightly coupled with combat missions, which not only retains the hierarchical and regular features of traditional combat networks but also features the characteristics of complex networks.

**Figure 3:**
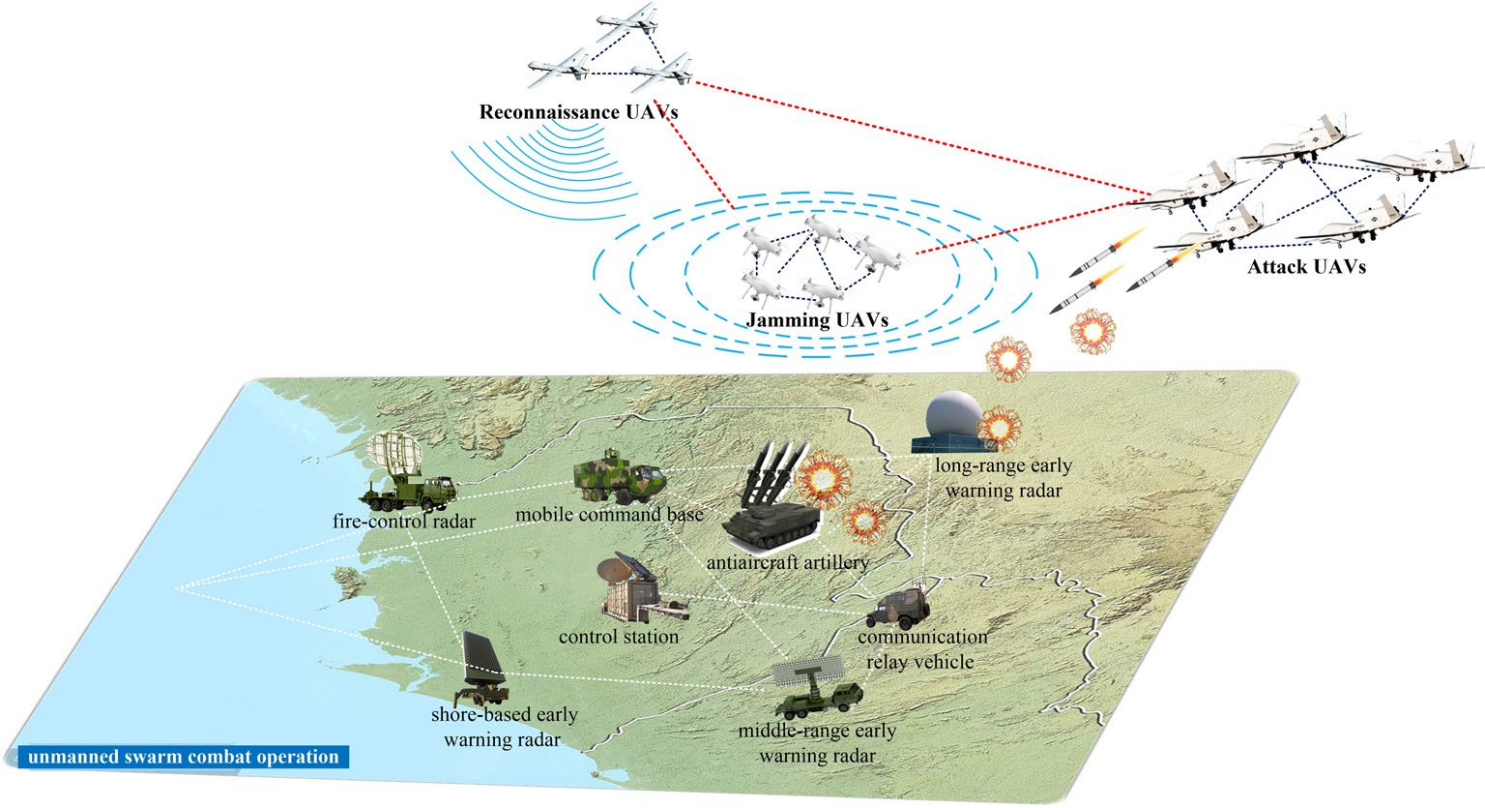
Sketch diagram of unmanned swarms attacking a ground force.

For instance, in a typical unmanned swarm attack on the ground or at sea^22^, unmanned swarms can be divided into multiple subswarms performing different tasks, such as intelligence reconnaissance, electromagnetic interference, and strikes. The subswarms are tightly coupled internally while loosely coupled with each other, exhibiting the organizational form of a “community network”. Figure 3 shows an example of unmanned swarms attacking a ground force. It is necessary to grasp the effective interactive transmission of information so that heterogeneous unmanned forces can be “dispersed in form but concentrated in spirit”. The top priority of network form design is to establish a mapping relationship between military requirements (e.g., information communication and network energy gathering) and complex network characteristics (e.g., scale-free and small-world networks).

### Design of coordination mechanism

In the military field, the winning mechanism of intelligent warfare is primarily manifested in "intelligence" and "autonomy". Therefore, the autonomy of various unmanned systems and platforms will have to be improved with the needs of the battlefield in the future. An unmanned swarm combat system will have higher perception, analysis, planning, decision-making, and execution capabilities and must be able to continuously complete the necessary control functions under uncertain object and environmental conditions and without human participation. Due to the regional distribution, intelligent autonomy, and decentralization of an unmanned swarm combat system, UAVs in unmanned swarms must conduct orderly collaboration and cooperation based on an information network, thereby ensuring high battlefield survivability and mission completion capabilities. Moreover, from the perspective of systems theory, the elements of unmanned swarms and those of the battlefield environment constitute a giant complex system in which the elements depend on, interact with, and restrict each other. The ultimate aim of collaboration among multiple unmanned platforms is to find the optimal control strategy for the entire giant system. Therefore, the design of the coordination mechanism is very important because it is the “soul” of swarm combat.

When interacting with other platforms, a single intelligent unmanned platform will inevitably calculate and evaluate its own energy, loss, cost, and behavioural cost to maximize its own payoff. This process is inevitably accompanied by competition among individuals, which causes the individual payoff to deviate from the optimal total utility of the swarms. Hence, keeping the individual payoff consistent with swarm utility is a key issue in the design of a coordination mechanism.

For example, in a fire strike mission, an intelligent combat platform with decision-making capabilities will prudently control the amount of firepower launched to maintain its own combat effectiveness; from the perspective of the swarms, the more firepower is contributed by each combat unit to the swarms, the higher the overall survival rate of the swarms and the greater the combat effectiveness^23–25^. The contradiction between the two will result in the tragedy of the commons^26^, which will not only lose the opportunity to win the battle but also provide the enemy with a counterattack opportunity.

The design of a high-quality coordination mechanism is the key to solving the contradiction between individual payoff and the total utility of the swarms. Currently, the competition and conflict between components (individuals) and the system (collective) remain to be further explored under classic multiagent system theory^27^, complex adaptive systems theory^28^, and the complex network theory framework.

## Related work

Autonomous collaboration is the ultimate state that unmanned swarms will reach. Rather than being achieved overnight, such a state will be realized by multiple iterations and gradual evolution. Therefore, the key to discovering the coordination mechanism and ultimately achieving autonomous collaboration in unmanned swarms is to explore the mechanism of collaborative evolution of unmanned swarms over time.

Based on the abovementioned military requirements, in this section, focusing on the collaborative evolution mechanism, we present a literature review on pioneering studies worldwide on swarm system dynamics, game-based swarm collaboration, and collaborative evolution of complex networks. The dynamic model is a powerful tool for revealing evolutionary mechanisms in swarm systems. Therefore, we first categorized the classic methods for modelling swarm dynamics; second, game theory was introduced into the swarm dynamics process in view of the unity of opposites of collaboration and conflict in swarm interaction; third, considering swarm evolution in spatial dimensions, we analysed the influence of spatial structure on dynamic evolution and the level of collaboration of unmanned swarms based on complex networks.

### Dynamic modelling of swarm systems

A swarm system, as a kind of complex dynamic evolutionary system, contains a large number of random and nonlinear factors, such as changes in the environment and interactions between individuals^29^. In a mathematical sense, dynamics (dynamic systems) refers to a discipline that studies the evolutionary patterns of systems under the action of various complex factors. Therefore, dynamic modelling of swarm systems is an effective tool for revealing the evolutionary mechanisms of swarm systems. Based on its degree of continuity, the dynamic process can be divided into the differential process (e.g., copying the dynamic equation^30^) and the Markov process (e.g., the average abundance transition probability^31,32^). Based on its spatial characteristics, the dynamic process can be divided into dynamic processes in Euclidean space (e.g., three-dimensional space) and dynamic processes in topological space (e.g., complex networks).

Swarm dynamics in Euclidean space involve two types of modelling ideas according to their spatial characteristics: spatially discrete Lagrangian modelling based on individuals and spatially continuous Eulerian modelling based on collective processes^33^. The specific models include the attraction–repulsion model^34^, fluid mechanics model^35^, Boids model^36^, particle swarms model^37^, and biological swarms (e.g., pigeon flocks^38^ and wolf packs^39^) model.

The collaborative evolution of swarms examined in this study is an example of dynamics in topological space. Dynamics in topological space cluster and split swarms according to different characteristics of individuals, e.g., behaviours, functions, and probability distributions. Vertical stratification and horizontal clustering are typical cases. Furthermore, the evolution of swarms in terms of behaviour, structure, and function is driven based on logical connections and communication between individuals. The modelling methods involved in swarm dynamics in topological space were classified in this study.

#### Networked systems and graph theory description

A swarm system in topological space refers to a networked system formed by the interaction of multiple individuals, which can be mathematically described and researched with graph theory. A swarm system can be represented by a graph $G = (V,E,A)$, where $V$ denotes a collection of nodes representing individuals, $E$ is a collection of edges that each describe the connection relationship between any two nodes, and $A$ stands for the adjacency matrix describing the association topology (coupling relationships and strength) of the swarm system^40,41^. When studying swarm dynamics using a graph, the Laplacian matrix and its algebraic eigenvalue properties are effective tools for describing and analysing graph topological structure^42^, and other concepts such as the connected graph^43^ and the spanning tree^44^ are also widely used in swarm dynamics.

#### Swarm dynamics model based on the cellular automaton

Since the cellular automaton was proposed by Von Neumann, it has quickly attracted widespread attention in the research field of swarm systems due to its simple local evolutionary rules and diverse and complex holistic emergent phenomena. The cellular automaton is a network dynamics model with discrete time, space, and state, where there is a causal and interactive relationship between space and time^45^. For a step size, the variable value of each cell is determined by the variable values of its neighbouring cells according to local rules. Therefore, a cellular automaton, with the ability to simulate the spatiotemporal evolution of networked complex systems, is an effective tool for studying the dynamic evolution of such systems.

#### Agent-based swarm dynamics model

Unlike the swarm dynamics model based on the cellular automaton, in agent-based swarm dynamic modelling, individuals are abstracted as agents with beliefs, desires, and intentions (BDIs). The theoretical basis of the agent-based swarm dynamic modelling method is that the global behaviour of a complex system is generated from the interactions between the low-level components of the system^46^. When modelling, we first need to build agents with different BDI attributes from the bottom to the top and then design the local interaction rules between the agents for swarm attributes to emerge based on the rules. This agent-based modelling method, which can simulate a variety of complex emergent behaviours of swarms, is particularly applicable to describing complicated and intelligent behaviours of swarms.

#### Swarm dynamic models based on game theory

If there is rationality or bounded rationality in the interactions between individuals in a swarm, game theory can be used to model the dynamic processes of swarm behaviours. All individuals are regarded as the participants in a game, with a variety of optional behaviours constituting the strategy set of the game. The group of individuals, the strategy set, and the corresponding payoff of each strategy form the game situation. Each individual selects a specific strategy by evaluating the influence of surrounding individuals and environmental factors and maximizing the payoff for the swarms and their individual payoff through adaptive learning during repeated games^47,48^. Finally, the mechanism of swarm behaviour emergence is revealed with the help of the Nash equilibrium of classic game theory or an evolutionarily stable strategy for an evolutionary game.

### Game-based swarm collaboration

In contrast to traditional optimization, swarm collaboration does not necessarily improve the adaptability of all individuals by simply selecting a specific behaviour. A more complicated situation is that in which individuals’ attempts to improve their interests are often in conflict with each other in interactions among individuals who directly influence each other. Unlike the traditional methods that maximize unilateral utility, game theory emphasizes the interdependence of individual strategies and focuses on analysing the interactions and effects of multiple individual behaviours, which reveal the unity of the opposites collaboration and conflict. Game theory provides an effective research framework for studying the interactions and coordination among multiple individuals in a swarm.

In contrast to classic game theory, evolutionary game theory^49–52^ holds that, due to the bounded rationality of individuals, the optimal equilibrium in an individual’s game cannot be found at the beginning, as in classic game theory; instead, individual strategies are modified and improved through abundant and repeated game processes. Evolutionary game theory describes the process of swarms eventually reaching an evolutionarily stable state by constantly adapting to the external environment through learning, imitation, and trial and error under the circumstances of imperfect rationality, asymmetric information, and deviations from the environment and expectations. Therefore, evolutionary game theory can describe the local dynamic nature of dynamic systems to predict individual behaviour and the emergence of swarm intelligence.

The application of evolutionary game theory in the field of swarm collaboration has been extended to various problems, including natural biological evolution^53–55^, environmental pollution control^56–58^, urban public resource construction^59,60^, and human cultural evolution^21,61,62^. Its application in the military domain mainly includes formation control, path planning, and mission assignment of unmanned swarms.

#### Formation control

Formation control aims to maintain a specific formation at the physical level and to guarantee a rational communication relationship at the information level^63^. One of the key factors is the division of labour; that is, an individual with any one role or function cannot independently complete a specific task that must be completed through the division of labour and collaboration between two or more different roles.

In an intelligence reconnaissance mission, collaboration between the intelligence unit and the information integration unit is required; a fire strike mission cannot be completed without close coordination among the intelligence, support, and firepower units. In such coordinated control, an important task is to effectively distribute strategies across complex networks so that individuals with different strategies can be distributed as homogeneously as possible around individuals with complementary strategies^24,64^. Adaptive collaborative control of swarms can be achieved by choosing the appropriate game type (e.g., the snowdrift game (SG) or the chicken game), designing a suitable method of calculating the payoff, and updating the evolutionary rules.

#### Path planning

Path planning, as an important research field of swarm collaboration, aims to make rapid decisions in complicated environments according to requirements (e.g., the shortest distance, minimum energy consumption, and the shortest time) and plan an optimal path from the starting point to the finishing point.

Compared with single individuals, path planning for swarm collaboration presents higher technical requirements. Since the individuals in a swarm have to compete against and cooperate with each other as well as adapt to the environment, both path planning for individuals and mutual collaboration among individuals are necessary^65,66^. The optimal path planning for individuals is not equivalent to the best path planning for the swarms. Therefore, the key is to coordinate the allocation of resources among individuals and realize a balance between individual needs and collective interest.

In swarm path planning based on evolutionary games, individuals are regarded as game players, path segments are taken as strategies, and the degree to which demands are satisfied is considered the game payoff. On this basis, the payoff function, evolutionary factors, and disturbance factors of individuals are constructed; individual behaviour is integrated with swarm utility; and the selection logic of individuals is simulated through evolution to realize collaboration where individuals obey the swarms and ultimately attain the evolutionarily stable strategy and the global optimal solution.

#### Mission assignment

An overall mission is divided into multiple subtasks. Individuals in the unmanned swarms perform their respective subtasks to achieve a division of labour at the collective level.

Since the costs for implementing different subtasks vary and the payoffs also differ, individuals with rationality and bounded rationality tend to prefer tasks that can result in higher payoffs at lower execution costs, especially when the payoffs of the tasks might be shared by all individuals. There may even be a “free-rider” phenomenon^67^, which could impair the execution of the overall mission, harm the interests of the swarms, and ultimately lead to the dilemma of division of labour^68,69^.

The reaction threshold model^70^, self-reinforcing model^71^, foraging model^72^, and network task assignment model^73^ are the traditional models used to realize mission assignment. These models are mostly inspired by the social behaviour of ant colonies and swarms, but they are not universally applicable to all domains. In evolutionary games, various methods for researching dynamics, such as replicator dynamics^30^, pairwise comparison^74^, and Moran processes^75^, are applied to map the missions to different strategies; the payoff function is designed based on the cost and payoff of the missions; and the missions are effectively assigned by adjusting the prevalence or fixed probability of each strategy. In recent years, researchers in various fields have attached importance to the exploration of mission assignment mechanisms by combining evolutionary games with complex networks^76–78^. An evolutionary game provides a unified framework for solving the problem of self-organized mission assignment from a new perspective.

According to the connectivity of individuals in topological space, swarms can be divided into well-mixed swarms and structured swarms. The former assumes that all individuals are connected (from the perspective of graph theory, the graph is fully connected), while the latter imposes the constraint that individuals only interact with their neighbours, thereby forming scale-free complex networks. The collaborative evolution of groups is actually a collaborative evolution within a spatial structure, so the integration of evolutionary game theory and complex networks holds great practical and application value. In fact, this combination has led to many interesting conclusions in specific fields, which have become new knowledge points.

#### Collaborative evolution mechanisms in complex networks

In 1992, Nowak and May introduced the concept of spatial dimensions into evolutionary game theory, pioneering the study of network evolutionary games^79,80^. Network evolutionary game theory uses the network to describe interactions between individuals and emphasizes the influence of network structure on the dynamic evolution and the level of collaboration of the swarms. Unlike the case of a well-mixed swarm, as long as the structure is appropriate, simple strategies can also maintain the survival of the cooperators.

The research team led by Nowak conducted theoretical derivations and numerical simulations of collective evolution in spatial structures such as regular lattices, doughnut charts, Erdős-Rényi random graphs, and small-world networks and innovatively proposed the relationship between the benefit–cost ratio (*b*/*c*) of a strategy and the average network connectivity degree *k*, pointing out that the lower the network connectivity is, the higher the probability for collaboration to arise by natural selection^81,82^. In addition, they used pairwise approximation theory to theoretically derive collaboration in regular lattices and obtained the boundary conditions for the generation and expansion of collaboration^83^. Based on the above research, by further analysing the differences between homogeneous and heterogeneous networks in the promotion of collaborative behaviours, weak connections were found to be more likely to promote collaboration in heterogeneous networks^40^. In a study of graphical multiparty games conducted by Pena et al., spatially structured games were found to be more likely to promote collaborative behaviours in collaborative games than were unstructured populations^41^. Collaborative evolution in the context of spatial structure has been extended to social networks to analyse the critical conditions for generating collaborative behaviours in human society^84^. In view of the contradiction between the probability and time of evolutionary convergence, Josef et al. preliminarily explored the tradeoff between fixation probability and fixation time in a spatially structured system^85^ and further extended collaborative evolution in structured populations to weighted graphs^86^. For N-person snowdrift games, past studies^87,88^ have investigated the relationship between the benefit–cost ratio (*b*/*c*) and the level of collaboration in well-mixed and structured populations and compared the significant differences between homogeneous/heterogeneous networks and unstructured populations in the promotion of collaboration.

Promising achievements in collaborative evolution mechanisms in complex networks have been attained by the research team led by Wang from Beijing University^89–94^, the team led by Zheng from Zhejiang University^95–98^, and the team led by Lyu from Beihang University. These researchers have conducted long-term and systematic studies on various evolutionary game models, such as the prisoner’s dilemma (PD), public goods, snowdrift, and deer hunting, as well as investigations of evolutionary dynamics and collaborative emergence mechanisms in complex networks, such as regular lattice networks, random graph networks, small-world networks, and free-scale networks.

Santos has played a leading role in the study of evolutionary games in scale-free networks. Due to the complexity of these networks, previous studies related to collaborative behaviours in scale-free networks have mainly been carried out by means of statistical simulation. We know the input and output but have no clue about the mechanism. Santos theoretically derived the internal mechanism of collaborative evolution by abstracting, simplifying, and approximating scale-free networks^99^. Based on a comparative analysis of collaborative behaviours in several special network structures, such as uniform attachment networks, configuration networks, and scale-free random networks, Santos revealed that the direct attachment between the scale-free nature (heterogeneity) of networks and nodes with many degrees of network connectivity are the core factors for the emergence of collaboration. The scale-free network based on the growth and preferential attachment mechanism provides a unified framework for the emergence of collaborative behaviours^100–103^.

Community networks, as a special type of complex network tightly coupled internally and loosely connected externally, are commonly found in natural biological communities, social groups, scientific research teams, and military swarms. Studies of collaborative evolution mechanisms in community networks have mainly been carried out from the following two aspects: exploring the influence of network degree, including average degree, internal degree, and external degree, on the level of collaboration based on specific game models and studying how the parameters of game models (e.g., the temptation of betrayal $b$ in PD and the benefit–cost ratio $r$ in SG) affect the collaborative level.

There have been staged and representative research results. Simulation analysis of the PD model on community networks showed that the collaboration level declined with the increase in the average degree of the network; given a constant average degree, swarm collaboration was enhanced by either increasing the internal degree $m$ or reducing the external degree $n$, while collaboration was inhibited by increasing $n$ with $m$ unchanged or increasing $m$ with $n$ remaining constant^90,104^. Through further exploration of PD and SG on community networks, collaboration was found to weaken with increasing $b$ in PD, and collaboration was inhibited with increasing $r$ in SG^20,105–107^. In recent years, some machine learning methods have been applied to the modeling and characteristic analysis of community networks. The literature^108^ develop a unified architecture of network community finding methods to characterize the state-of-the-art of the field of community detection, and in^109^, a novel redefined SBM (Stochastic blockmodel) with Poisson distribution and its block-wise learning algorithm that can efficiently analyze large-scale networks are proposed. Nevertheless, compared with well-mixed swarms, given the same parameters, the level of collaboration was significantly improved.

The challenges for the study of collaborative evolution on community networks include the design of a rational community generation mechanism based on the specific research background, selection of a suitable evolutionary game model, and construction of a dynamic process that satisfies practical needs. A community network also serves as a spatial structure foundation for the collaborative evolution of unmanned swarms in this study. Considering the dynamic reconstruction of the underlying network structure in evolution, collaborative evolution on complex networks has developed towards coevolution, namely, the coevolution of the strategy and the underlying interactive network topology^110^.

In this section, we review studies worldwide on swarm collaboration from three perspectives. The above research findings are of great theoretical and engineering application value. However, they cannot be directly transplanted to the field of unmanned swarm operations to solve the problem of autonomous collaboration.

In “Unmanned swarms in community networks”, a community information network based on the operational capabilities required of unmanned swarms is designed. In “Unmanned swarm collaboration model based on multiplayer public goods evolutionary game”, we abstract collaboration in unmanned swarms into an N-person evolutionary game with multiple rounds of iterations, establish a collaborative evolution model of unmanned swarms based on the evolutionary game of multiple public goods, and present the evolutionary dynamic process of unmanned swarms in complex community networks. In the last section, through a simulated analysis, some reasonable suggestions for promoting collaboration in unmanned swarm combat are proposed.

## Unmanned swarms in community networks

The information network can maintain the static form to a certain extent when the confrontation between us and the enemy is not fierce or in a short time interval of the whole cycle of the confrontation. Therefore, this study starts with a relatively simple situation and assumes that the swarm information network is a static network.

Information networks for unmanned swarms must be able to reflect the composition rules and principles of the battlefield combat system. First, a network should be capable of revealing the correct flow of materials, energy, and especially information; second, a network should be able to reflect the informational cohesion of homogeneous (functional) combat units as well as the loose coupling of information among heterogeneous units. Moreover, considering operational commands, we must take into account the hierarchical nature of the information network. According to the requirements concerning information network construction in “Analytical framework for autonomous collaborative behaviour in unmanned swarms”, we propose in this section a method of constructing community networks for unmanned swarms and analyse network characteristics such as degree distribution. A community network functions as the spatial basis for collaborative evolutionary behaviours in unmanned swarms.

### Network construction

We modelled a military operation in which a total of $M\left( {M \ge 2} \right)$ unmanned swarms with heterogeneous functions were required to collaboratively complete the combat missions. The community network was generated by inner-community preferential attachment and inter-community preferential attachment as follows:

#### Initialization

Each community was initially composed of $m_{0} \left( {m_{0} > 1} \right)$ fully connected nodes. A fixed node was randomly selected in each community, and each community was connected with the remaining $M - 1$ communities with $C_{M}^{2}$ edges to ensure that every two communities were connected by one edge.

**Figure 4:**
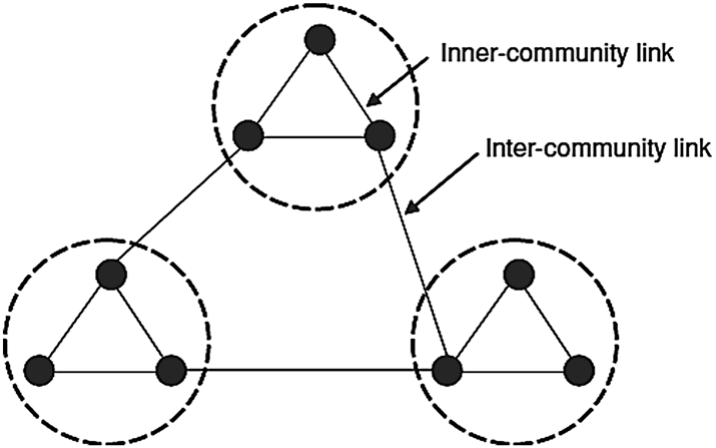
Initial network with $M = 3$ and $m_{0} = 3$.

Figure 4 shows an initial network with $M = 3$ and $m_{0} = 3$.

#### Growth

In each time step, a new node was added to a random community. The new node was connected to $m(1 \le m \le m_{0} )$ currently available nodes in the community through $m$ edges and to the $n(0 \le n < m)$ nodes in the other $M - 1$ communities through $n$ edges.

#### Preferential attachment

Preferential attachment occurred simultaneously between internal nodes and external nodes.

##### Inner-community preferential attachment

When a new node was internally attached to a randomly selected community (e.g., the $j$-th community), the probability $\prod_{{s_{ij} }}$ of node $i$ in community $j$ being selected was proportional to its internal degree $s_{ij}$.

1 $$\prod_{{s_{ij}}} = \frac{{s_{ij}}}{{\sum\nolimits_{k} {s_{kj}}}}$$

##### Inter-community preferential attachment

When a new node was externally attached to node $i$ in the $k(k \ne j)$-th community, the probability $\prod_{{l_{ik} }}$ of node $i$ being selected was proportional to its external degree $l_{ik}$.

2 $$\prod_{{l_{ik} }} = \frac{{l_{ik} }}{{\sum\nolimits_{i,k \ne j} {l_{ik} } }}$$

After repeating the three steps described above, a community network with $M$ communities, $Mm_{0} + t$ nodes, and $(MC_{{m_{0} }}^{2} + C_{M}^{2} ) + (m + n)t$ edges was generated after $t$ time steps.

### Analysis of network characteristics

$s_{ij}$ is assumed to be continuous. According to the mean field theory^106^, $\prod_{{s_{ij} }} = {{s_{ij} } \mathord{\left/ {\vphantom {{s_{ij} } {\sum\nolimits_{k} {s_{kj} } }}} \right. \kern-\nulldelimiterspace} {\sum\nolimits_{k} {s_{kj} } }}$ is approximately averaged as the continuous rate of change in $s_{ij}$. Therefore, for node $i$ in community $j$, the following equation is obtained:

3 $$\frac{{\partial_{{s_{ij} }} }}{{\partial_{t} }} = A\frac{{s_{ij} }}{{\sum\nolimits_{k} {s_{kj} } }}$$

For community $j$, $\Delta s_{j} = {m \mathord{\left/ {\vphantom {m M}} \right. \kern-\nulldelimiterspace} M}$ and $s_{ij} = \sum\nolimits_{k} {s_{kj} } = 2mt\frac{1}{M} + m_{0} (m_{0} - 1)$. Therefore, the following equation is obtained:

4 $$\frac{{\partial_{{s_{ij} }} }}{{\partial_{t} }} = \frac{m}{M}\frac{{s_{ij} }}{{\sum\nolimits_{k} {s_{kj} } }}$$

Over a long time $t$, $\sum\nolimits_{k} {s_{kj} } = 2mt\frac{1}{M} + m_{0} (m_{0} - 1) \approx 2mt\frac{1}{M}$, and thus the following equation is obtained: $\frac{{\partial_{{s_{ij} }} }}{{\partial_{t} }} \approx \frac{{s_{ij} }}{2t} \Rightarrow \frac{{\partial_{{s_{ij} }} }}{{s_{ij} }} \approx \frac{{\partial_{t} }}{2t}$, that is

5 $$s_{ij} (t) \approx c(t)^{0.5}$$

At a time step $t_{i}$, a new node $i$ is added into swarms $j$, so the initial conditions of $s_{ij} (t_{i} ) = m$ are satisfied. By substituting $s_{ij} (t_{i} ) = m$ into the above equation, the following equation is obtained:

6 $$s_{{ij}} (t) \approx m\left( {\frac{t}{{t_{i} }}} \right)^{{0.5}}$$

Then, the probability of the node degree being lower than $k$ satisfies the following equation:

7 $$P(s_{{ij}} (t) < k) = P\left( {t_{i} > \frac{{m^{2} t}}{{k^{2} }}} \right)$$

Assuming that all nodes, including the initial nodes, are added to the network with the same time step (interval), then time step $t_{i}$ is a homogeneously distributed random variable whose probability density can be expressed as follows:

8 $$P_{i} (t_{i} ) = \frac{1}{{Mm_{0} + t}}$$

By substituting Eq. (8) into Eq. (6), the following equation is derived:

9 $$P\left( {t_{i} > \frac{{m^{2} t}}{{k^{2} }}} \right) = 1 - P\left( {t_{i} \le \frac{{m^{2} t}}{{k^{2} }}} \right) = 1 - \frac{{m^{2} t}}{{k^{2} (Mm_{0} + t)}}$$

By taking the derivative of $k$ in the above equation, the probability density of $P(k)$ is obtained as follows:

10 $$P(k) = \frac{{\partial P(s_{ij} (t) < k)}}{\partial k} = \frac{{2m^{2} t}}{{Mm_{0} + t}}k^{ - 3}$$

This probability density function obeys $P(k)\sim k^{ - \gamma }$, the power-law distribution pattern of $\gamma = 3$.

Similarly, the external degree distribution of nodes satisfies the following equation:

11 $$\frac{{\partial_{{l_{ik} }} }}{{\partial_{t} }} = \frac{M - 1}{M}n\frac{{l_{ik} }}{{\sum\nolimits_{m,n,n \ne j} {l_{mn} } }}$$

where $\sum\nolimits_{m,n,n \ne j} {l_{mn} } = 2\frac{M - 1}{M}nt + [M(M - 1) - (M - 1)]$, and the solution to the above equation can be approximated as follows:

12 $$l_{{ik}} (t) = n\left( {\frac{{t + \beta }}{{t_{j} + \beta }}} \right)^{{0.5}}$$

where $\beta = {{[M(M - 1) - (M - 1)]M} \mathord{\left/ {\vphantom {{[M(M - 1) - (M - 1)]M} {2n(M - 1)}}} \right. \kern-\nulldelimiterspace} {2n(M - 1)}}$. When $t$ is large, $2\frac{M - 1}{M}nt > > [M(M - 1) - (M - 1)]$. Therefore, ${{\partial_{{l_{ik} }} } \mathord{\left/ {\vphantom {{\partial_{{l_{ik} }} } {\partial_{t} }}} \right. \kern-\nulldelimiterspace} {\partial_{t} }} \approx {{l_{ik} } \mathord{\left/ {\vphantom {{l_{ik} } {2t}}} \right. \kern-\nulldelimiterspace} {2t}}$; that is,

13 $$l_{{ik}} (t) \approx n\left( {\frac{t}{{t_{j} }}} \right)^{{0.5}}$$

Hence, the degree distribution of external attachment is expressed as follows:

14 $$P(k) = \frac{{2n^{2} t}}{{Mm_{0} + t}}k^{ - 3}$$

This probability density function obeys $P(k)\sim k^{ - \gamma }$, the power-law distribution pattern of $\gamma = 3$.

Based on the above conclusions, $k_{ij} (t)$, the degree of node $i$ in community $j$, is expressed as follows:

15 $$k_{{ij}} (t) = s_{{ij}} (t) + l_{{ij}} (t) \approx (m + n)\left( {\frac{t}{{t_{i} }}} \right)^{{0.5}}$$

Then, the degree distribution of the entire community network is expressed as follows:

16 $$P(k) = \frac{{2(m + n)^{2} t}}{{Mm_{0} + t}}k^{ - 3}$$

In other words, the entire community network also obeys the power-law distribution pattern of $\gamma = 3$.

In addition, since $n < m$, based on Eqs. (6) and (13), the external degree of nodes is always lower than the internal degree, which further verifies the above features of community networks.

## Unmanned swarm collaboration model based on multiplayer public goods evolutionary game

Multicluster unmanned swarm collaboration with community structure is essentially a multiplayer evolutionary game with multiple iterations. Among the many evolutionary game models, the public goods game (PGG) provides a basic theoretical framework for revealing the collaborative evolution mechanism and solving the tragedy of public resources. With investment in public goods as the background, this game model portrays the process during which the cooperators and defectors (free riders) conduct strategic games based on parameters such as cost, multiplication factor, and selection intensity with the passage of time so that the proportions of cooperators and defectors in the swarms dynamically change and eventually reach an evolutionarily stable state. In the PGGs, balancing individual payoff and swarm utility and increasing the proportion of cooperators are important prerequisites for solving the tragedy of the commons and realizing autonomous collaboration of unmanned swarms.

In this section, based on the framework of the multiplayer public goods evolutionary game, we model the collaborative evolution of multicluster unmanned swarms on community networks and present the corresponding evolutionary game framework and dynamic process.

### Framework of multiplayer public goods evolutionary game

Evolutionary game theory combines “equilibrium” in economics with “adaptability” in biology to depict the process by which individuals adapt to the external environment through learning, imitation, and trial-and-error under boundary rationality and asymmetric information. The PGG provides a basic theoretical framework for revealing the cooperative evolution mechanism and coping with the tragedy of the commons. PGG reflects that investors (collaborators) and hitchhikers (non-collaborators) play strategic games over time based on cost, multiplication factor, selection intensity, etc., which makes the proportion of collaborators and betrayers in the population change dynamically and finally tends to an evolutionarily stable state (ESS). The research focus of PGG is to calculate the mathematical expectation of the proportion of collaborators in a population after a multi-round game, that is, the average abundance, and then analyse the relationship between the average abundance and parameters (i.e., cost, multiplication factor, selection intensity, etc.) to achieve the ultimate purpose of manual control.

In essence, the autonomous collaboration of unmanned swarms is a multiparty and multi-round game process that focuses on the autonomous allocation of public resources. Therefore, we use a multiplayer public goods evolutionary game to model the cooperative evolution of unmanned swarms.

Table 1 shows the relationship between concepts of unmanned swarms on community networks and the multiplayer PGGs.

**Table 1:** Relationships between concepts of unmanned swarms on community networks and the multiplayer public goods game.

Unmanned swarm collaborative evolution	Public goods evolutionary game
Unmanned swarms	Spatially structured population
Clusters	Community networks
Public resources (e.g., bombs and communication)	Public goods
Single unmanned platform	Individual
Multiple unmanned platforms participating in collaborative combat	Multiple players
Unmanned platforms contribute resources to the swarms	Collaboration strategy (*C*)
“Free-riding” phenomenon among platforms refusing to contribute resources	Defection Strategy (*D*)
Resources obtained by platforms under specific spatial structures and strategies	Payoff
Platforms’ payoff-based strategy conversion under specific spatial structures	Game
Dynamic change in proportion of platforms holding different strategies in multiple rounds of games	Evolution
The proportions become stable after multiple rounds of games, and the games are terminated	Evolutionarily stable state

In the specific application scenario, a single unmanned platform in the swarm acts as an individual. A swarm composed of multiple unmanned platforms with a common mission (e.g., fire attack on the same position and intelligence reconnaissance on the same area). A single unmanned platform has different optional behaviour modes (such as dropping ammunition, dropping ammunition of different equivalents, and not dropping ammunition) as a game strategy. At every moment, the platform interacts with its “neighbours” (other unmanned platforms with physical connections based on geographical location and logical connections based on information communication). According to its own and its neighbours’ strategies, it obtains certain combat effectiveness and certain benefits (payoff). An intelligent unmanned platform with independent decision-making ability changes its behaviour mode (strategy update) by evaluating its payoff. After multiple rounds of games and repeated strategy updates among individuals, a high degree of synergy has emerged at the swarm level, making swarm control finally reach the target state (such as consistency, synchronization, division of labour, etc.). In the above process, the selection of game type, the design of income calculation method, and the determination of strategy update rules are very important. The above factors are the key to the realization of swarm independent cooperation goals.

The evolutionary game occurs in multicluster unmanned swarms on community networks. With the progression of multiple rounds of evolution, a single unmanned platform $i$ constantly updates its strategy in the strategy set $\{C,{\kern 1pt} {\kern 1pt} {\kern 1pt} {\kern 1pt} D\}$ according to the payoffs for itself and its neighbours until the proportions of platforms with different strategies in the swarms stabilize.

Multiplayer games can be regarded as a superposition of multiple two-player games or an expansion of traditional two-player games with the characteristic of “multiplayer interaction” embedded into the payoff^98^. Based on the second conceptualization, on a community network with scale $N$, $k_{i} = k_{i}^{C} + k_{i}^{D}$ denotes the degree of $i$, where $k_{i}^{C}$ and $k_{i}^{D}$ are the numbers of individuals holding *C* and *D* strategies, respectively, among the neighbours of $i$ in a certain round of the game.

1. If $i$ selects collaboration strategy *C*, the total contribution of all players in the multiplayer PGGs composed of $i$ and its neighbours would be $k_{i}^{C} c + c$, where $c$ is the amount of resources contributed by a single player to the swarms, $r(k_{i}^{C} c + c)$ denotes the total output multiplied by the multiplication factor $r$, and ${{r(k_{i}^{C} c + c)} \mathord{\left/ {\vphantom {{r(k_{i}^{C} c + c)} {(k_{i} + 1}}} \right. \kern-\nulldelimiterspace} {(k_{i} + 1}})$ represents the payoff of each individual. Since $c$ is the cost of $i$, the net payoff of $i$ is ${{r(k_{i}^{C} c + c)} \mathord{\left/ {\vphantom {{r(k_{i}^{C} c + c)} {(k_{i} + 1}}} \right. \kern-\nulldelimiterspace} {(k_{i} + 1}}) - c$.
2. If $i$ chooses defection strategy *D*, the total contribution of all players in the multiplayer PGGs composed of $i$ and its neighbours would be $k_{i}^{C} c$, the total output would be $rk_{i}^{C} c$, and the payoff for each individual would be ${{rk_{i}^{C} c} \mathord{\left/ {\vphantom {{rk_{i}^{C} c} {(k_{i} + 1)}}} \right. \kern-\nulldelimiterspace} {(k_{i} + 1)}}$. Since $i$ contributes nothing, its net payoff is ${{rk_{i}^{C} c} \mathord{\left/ {\vphantom {{rk_{i}^{C} c} {(k_{i} + 1)}}} \right. \kern-\nulldelimiterspace} {(k_{i} + 1)}}$. Let $a_{{k_{i}^{C} }}$ and $b_{{k_{i}^{C} }}$ be the payoffs of individuals who select strategies *C* and *D*, respectively; then,
  17 $$a_{{k_{i}^{C} }} = {{r(k_{i}^{C} c + c)} \mathord{\left/ {\vphantom {{r(k_{i}^{C} c + c)} {(k_{i} + 1}}} \right. \kern-\nulldelimiterspace} {(k_{i} + 1}}) - c$$
  18 $$b_{{k_{i}^{C} }} = {{rk_{i}^{C} c} \mathord{\left/ {\vphantom {{rk_{i}^{C} c} {(k_{i} + 1)}}} \right. \kern-\nulldelimiterspace} {(k_{i} + 1)}}$$

The payoff matrix is shown in Table 2.

**Table 2:** Payoff matrix of multiplayer public goods evolutionary game.

	$k_{i}$	$\cdots$	$k_{i}^{C}$	$\cdots$	1	0
*C*	$rc - c$	$\cdots$	${{r(k_{i}^{C} c + c)} \mathord{\left/ {\vphantom {{r(k_{i}^{C} c + c)} {(k_{i} + 1}}} \right. \kern-\nulldelimiterspace} {(k_{i} + 1}}) - c$	$\cdots$	${{2rc} \mathord{\left/ {\vphantom {{2rc} {(k_{i} + 1)}}} \right. \kern-\nulldelimiterspace} {(k_{i} + 1)}} - c$	${{rc} \mathord{\left/ {\vphantom {{rc} {(k_{i} + 1)}}} \right. \kern-\nulldelimiterspace} {(k_{i} + 1)}} - c$
*D*	${{rk_{i} c} \mathord{\left/ {\vphantom {{rk_{i} c} {(k_{i} + 1)}}} \right. \kern-\nulldelimiterspace} {(k_{i} + 1)}}$	$\cdots$	${{rk_{i}^{C} c} \mathord{\left/ {\vphantom {{rk_{i}^{C} c} {(k_{i} + 1)}}} \right. \kern-\nulldelimiterspace} {(k_{i} + 1)}}$	$\cdots$	${{rc} \mathord{\left/ {\vphantom {{rc} {(k_{i} + 1)}}} \right. \kern-\nulldelimiterspace} {(k_{i} + 1)}}$	$0$

Based on Eqs. (17) and (18), given the same network structure with $k_{i}^{C}$ and $k_{i}^{D}$ remaining constant, the payoff for individual $i$ to choose strategy *D* would always be higher than the payoff brought by selecting strategy *C*. Therefore, from the individual perspective, $i$ should prefer to choose strategy *D* to gain greater benefits, e.g., battlefield survivability, by “exploiting” strategy *C* holders among its neighbours through “free-riding”; at the swarm level, the more resources are contributed by individuals to the swarm, the better the overall combat effectiveness. There is an uncompromising contradiction between individual selfishness and the overall demands of the swarms. Hence, increasing the proportion of collaboration strategy holders in the swarm while avoiding the tragedy of the commons is an important problem in the research on and practical application of unmanned swarm technology.

### Dynamic processes of collaborative evolution in unmanned swarms

Unconditional imitation^88^, replicator dynamics^30^, and the Fermi rule^107^ are the current typical strategy update rules in evolutionary games. Among them, the Fermi rule emphasizes comparing the payoff to an individual with the payoffs to its neighbours. Driven by this rule, the probability of individual $i$ switching its strategy in strategy set $\{ C,{\kern 1pt} {\kern 1pt} D\}$ is as follows:

19 $$P_{{S_{i} \in \{ C,{\kern 1pt} {\kern 1pt} D\} }} = \frac{1}{{1 + e^{{\omega (F_{i} - \overline{{F_{{k_{i} }} }} )}} }}$$

where $\omega \in [0{\kern 1pt} ,{\kern 1pt} {\kern 1pt} {\kern 1pt} {\kern 1pt} 1]$ is the selection intensity that can enlarge or reduce the influence of $F_{i} - \overline{{F_{{k_{i} }} }}$ on the strategy update probability, and the reality test shows that a weaker selection intensity ($\omega < < 1$) promotes collaboration^110,111^. $F_{i} \in \{ a_{{k_{i}^{C} }} ,{\kern 1pt} {\kern 1pt} {\kern 1pt} b_{{k_{i}^{C} }} \}$ denotes the payoff to individual $i$, and $\overline{{F_{{k_{i} }} }}$ stands for the average payoff to its $k_{i}$ neighbours. Let $\Delta = F_{i} - \overline{{F_{{k_{i} }} }}$. If $\Delta = 0$, $P_{{S_{i} \in \{ C,{\kern 1pt} {\kern 1pt} D\} }} = {1 \mathord{\left/ {\vphantom {1 2}} \right. \kern-\nulldelimiterspace} 2}$, and the unmanned platforms hold the same preference for strategies *C* and *D*; if $\Delta > 0$ (that is, $F_{i}$ is higher than $\overline{{F_{{k_{i} }} }}$), the unmanned platforms are more likely to maintain the current strategy; and if $\Delta < 0$ (that is, $F_{i}$ is lower than $\overline{{F_{{k_{i} }} }}$), then $P_{{S_{i} \in \{ C,{\kern 1pt} {\kern 1pt} D\} }} > {1 \mathord{\left/ {\vphantom {1 2}} \right. \kern-\nulldelimiterspace} 2}$, and individuals are more inclined to switch the current strategy to another strategy in the strategy set $\{ C,{\kern 1pt} {\kern 1pt} D\}$.

The dynamic processes of collaborative evolution in unmanned swarms on community networks were abstracted into the following four steps.

1. According to the community network generation algorithm, a multicluster unmanned swarm network with scale $N$ and number of communities $M$ is generated.
2. Random strategy distribution is implemented on $N$ network nodes, with the holders of strategies *C* and *D* accounting for approximately 50% each.
3. Individual $i$ forms a game group $G$ with all its neighbours with direct network connections, the game is conducted according to the framework of a multiplayer public goods evolutionary game, and $F_{i}$ and $\overline{{F_{{k_{i} }} }}$ are calculated.
4. After each game round, individual $i$ evaluates the payoff of the current strategy and then updates the strategy according to the Fermi rule.

**Figure 5:**
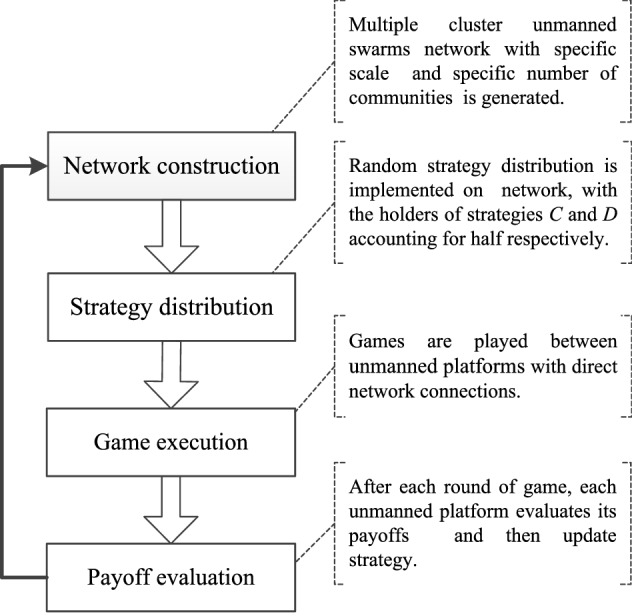
The dynamic processes of collaborative evolution in unmanned swarms.

The process is illustrated in Fig. 5.

The above steps were repeated until the proportion of individuals holding a certain type of strategy in the entire swarm reached a stable state.

Multi-cluster unmanned swarm collaboration with community structure is essentially an evolutionary game process with multiple participants and multiple rounds of iteration. How to build an unmanned swarm cooperation evolution model based on the evolutionary game framework of multiple public goods; analyse the swarm scale, network degree distribution, and the impact of key parameters such as cost and multiplication factor on the level of swarm cooperation; and explore the emergence mechanism of unmanned swarm cooperation are crucial and urgent problems in the research and practical application of unmanned swarm technology.

## Analysis of mechanisms of collaborative evolution

Based on the community network construction algorithm in “Unmanned swarms in community networks” and the framework and dynamic processes of the multiplayer public goods evolutionary game in “Unmanned swarm collaboration model based on multiplayer public goods evolutionary game”, we simulated and analysed the influence of swarm size $N$, average degree $\overline{k}$, relationship between intra-community attachment and inter-community attachment ${m \mathord{\left/ {\vphantom {m n}} \right. \kern-\nulldelimiterspace} n}$, cost $c$ and multiplication factor $r$ on collaborative behaviours in unmanned swarm combat.

### Influence of swarm size and network degree on collaboration

In unmanned swarm operations, swarm scale is an important consideration. If the scale is too large, the combat cost (human, material, and financial resources) increases, while if the scale is too small, the expected operational effectiveness may not be achieved. Therefore, it is necessary to find the mechanism of the influence of swarm scale on cooperation to guide combat design.

**Figure 6:**
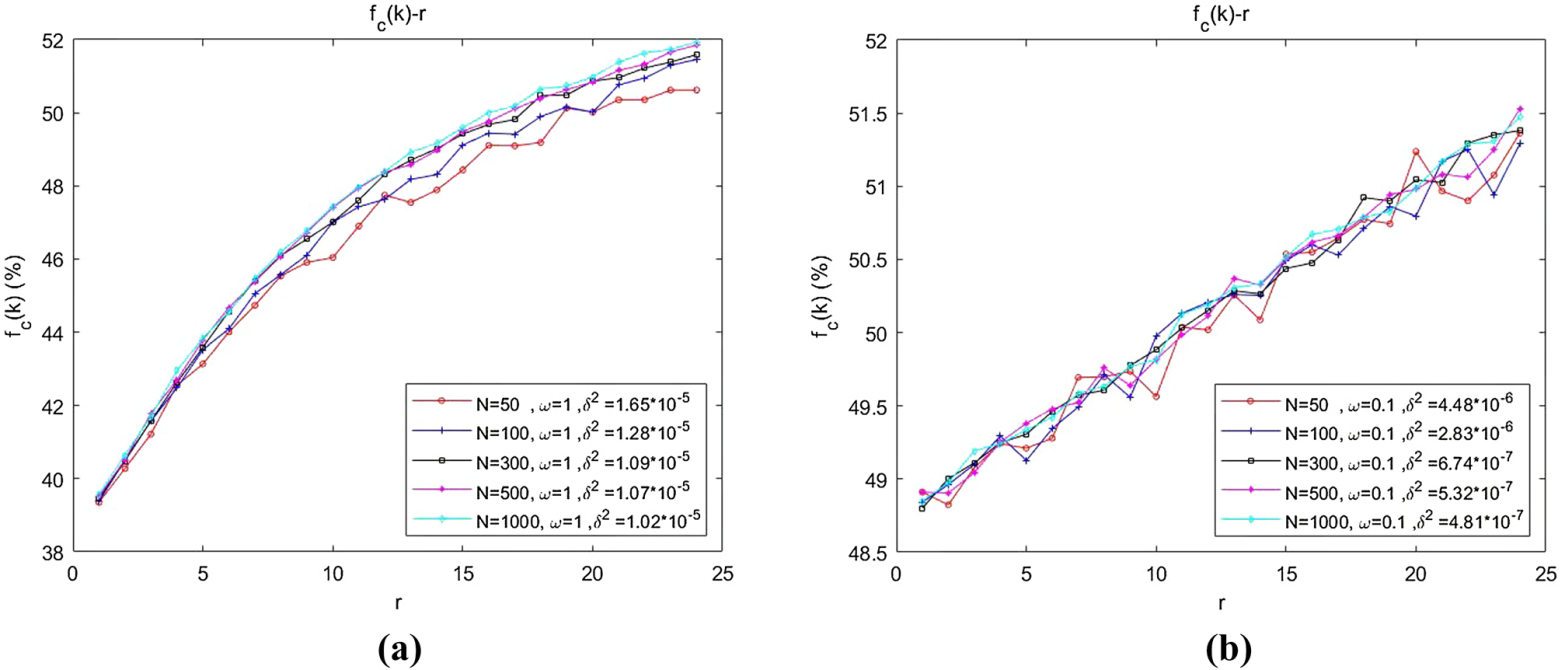
(**a**) Dependence of the emergence of collaboration on swarm size with strong selection; (**b**) dependence of the emergence of collaboration on swarm size with weak selection.

Let *N* = 50, 100, and 300 and $\overline{k} \approx 5$. The curve of the relationship between swarm abundance $f_{C} (k)$ and multiplication factor $r$ was simulated and mapped under weak selection ($\omega = 0.1$) and strong selection ($\omega = 1$), as shown in Fig. 6. The simulation settings were as follows: each data point was operated 100 times (10 implementations of network topology corresponded to 10 distributions of initial policies) and then averaged; for each operation, cooperators and defectors were randomly distributed in equal proportions on the network. After 10,000 steps of evolution, the results of 2000 steps were averaged. Unless otherwise specified, the subsequent simulation settings were the same.

As shown in Fig. 6, when the selection intensity and average degree remained constant, the swarm abundance curves under different swarm sizes were basically consistent. Furthermore, given a fixed selection intensity, the influence of swarm size on the stability of swarm abundance was investigated. The slope of each segment connecting adjacent data points was calculated, and the variance $\delta^{2}$ of the slope was calculated to characterize the stability of swarm abundance (the smoothness of the curve). As shown in Fig. 6a, $\delta^{2} \left| {_{{N{ = 10}00}} } \right. < \delta^{2} \left| {_{{N{ = 5}00}} } \right. < \delta^{2} \left| {_{{N{ = }300}} } \right. < \delta^{2} \left| {_{{N{ = 1}00}} } \right. < \delta^{2} \left| {_{{N{ = 5}0}} } \right.$, and the inequation is still valid in Fig. 6b. Hence, the larger the swarm size is, the more stable the swarm abundance.

We try to qualitatively analyse the above simulation results. Since each data point is set in the simulation, the average is calculated after 100 operations. For each operation, after 10,000 evolution time steps, the results of 2000 steps are averaged. Therefore, the value of each data point in the figure is a result in a mathematical and statistical sense. When the swarm scale is relatively small, the randomness of the results is relatively large, which is reflected in the jump between data points on the graph; that is, the trend between data points is poor. When the scale is relatively large, the randomness of the results decreases, and the trend between data points will be more obvious, which shows that the smoothness of the curve increases.

#### Conclusion 1

The emergence of collaboration in unmanned swarms on community networks depends nonsignificantly on swarm size; however, the smaller the swarm size is, the more unstably the swarm abundance varies with the multiplication factor.

Because the topology of an unmanned swarm is a scale-free complex network, it is of guiding value for the construction of a swarm network to investigate the impact of the network average on the level of cooperation. Furthermore, it is of particular relevance to investigate the strategy distribution on nodes with different degrees for targeted regulation of swarm collaborative behaviour.

**Figure 7:**
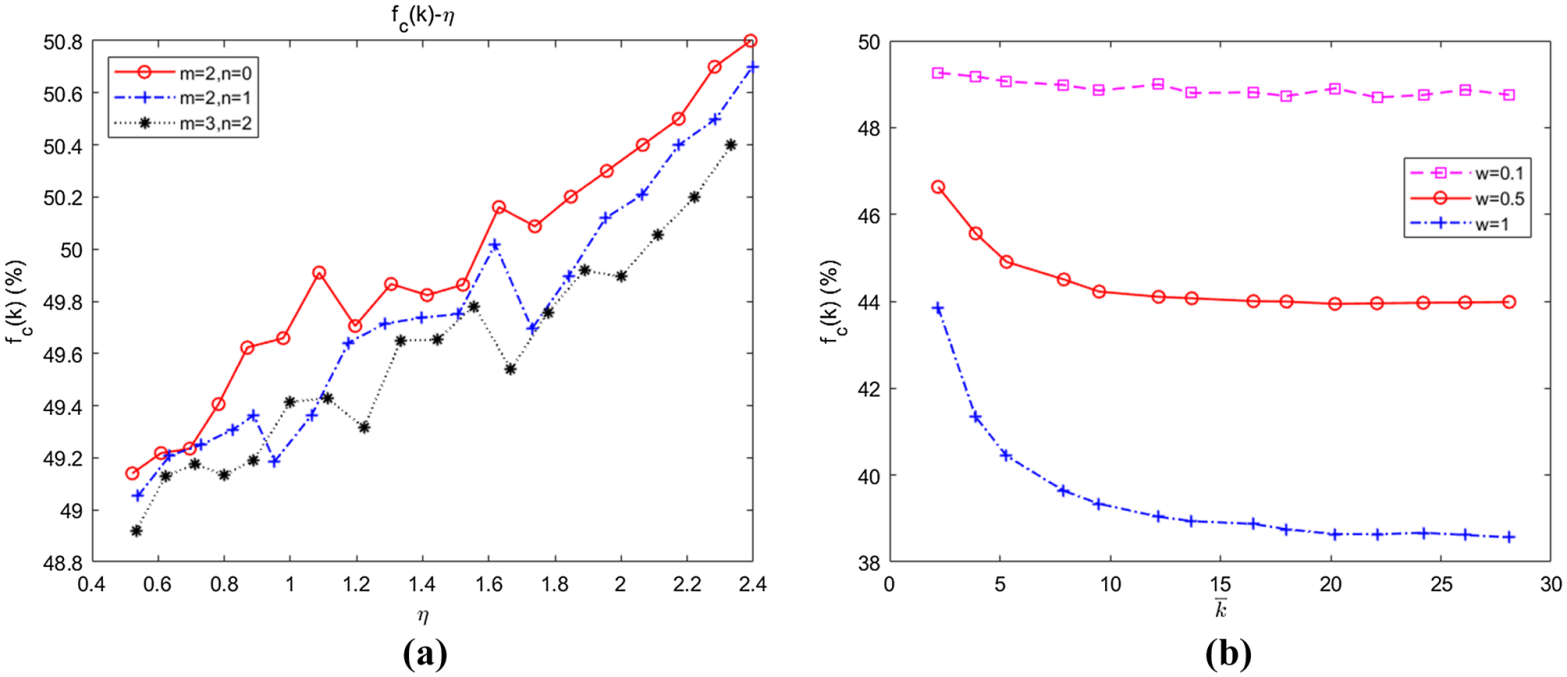
(**a**) Dependence of the emergence of collaboration on $\eta$; (**b**) dependence of the emergence of collaboration on average degree.

Through basic mathematical substitution and rearrangement of Eqs. (17) and (18), we obtained $a_{{k_{i}^{C} }} = b_{{k_{i}^{C} }} + [{r \mathord{\left/ {\vphantom {r {(\overline{k} + 1)}}} \right. \kern-\nulldelimiterspace} {(\overline{k} + 1)}} - 1] \cdot c$. Therefore, the coefficient ${r \mathord{\left/ {\vphantom {r {(\overline{k} + 1)}}} \right. \kern-\nulldelimiterspace} {(\overline{k} + 1)}}$ was of great importance to individuals’ selection of strategies. Let $\eta { = }{r \mathord{\left/ {\vphantom {r {(\overline{k} + 1)}}} \right. \kern-\nulldelimiterspace} {(\overline{k} + 1)}}$. The curve of the relationship between swarm abundance and the coefficient $\eta$ under different values of $m$ and $n$ was obtained, as shown in Fig. 7a. On this basis, $\overline{k}$ was isolated, and the relationship between swarm abundance and average degree was further examined, as shown in Fig. 7b.

As shown in Fig. 7a, given the same $\eta$, with the increase in average degree $\overline{k}$ (according to the process of generating community networks described in “Network construction”, the average degree of nodes $\overline{k} \approx 2(m + n)$), even if the multiplication factor $r$ were large, collaboration on the community network would be suppressed, and $f_{C} \left| {_{{\overline{k} \approx 4}} } \right. > f_{C} \left| {_{{\overline{k} \approx 6}} } \right. > f_{C} \left| {_{{\overline{k} \approx 10}} } \right.$. As shown in Fig. 7b, as $\overline{k}$ further increased, the collaboration in the swarms was inhibited; especially under strong selection, collaboration in the swarms collapsed sharply, with the collaborative level dropping below 40% when $\overline{k} > 5$. The probable reason was that the increased average degree resulted in closer links between nodes, which made it easier for defectors to “exploit” the cooperators by means of free-riding to gain greater benefits and to better survive.

#### Conclusion 2

The increase in average degree decreases the average abundance of swarms on community networks; that is, the close connection between nodes restrains the emergence of collaborative behaviours in unmanned swarms.

**Figure 8:**
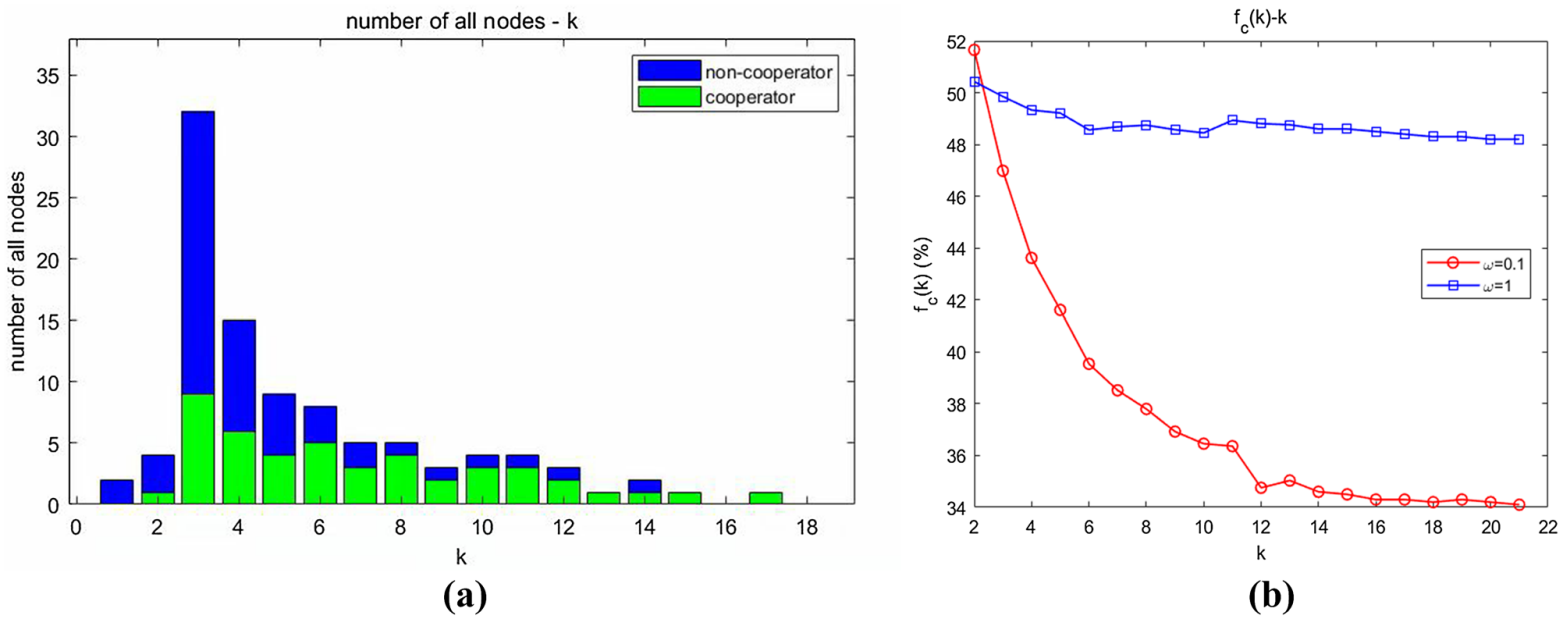
Degree distribution and dependence of the emergence of collaboration on degree. (**a**) Degree distribution in a single calculation; (**b**) dependence of the emergence of collaboration on degree.

The scale-free nature of the community network constructed in this study is demonstrated in “Analysis of network characteristics”. Therefore, investigation of the distribution of strategies among nodes differing in the degree of network connectivity is of great value to elucidating the emergence of collaboration. Figure 8a shows the degree distribution, and Fig. 8b shows the dependence of the emergence of collaboration on degree. The simulation parameters were set as follows: $N = 100$, $m = 2$, $n = 1$, and $r = 5$.

Figure 8a shows the degree distribution in a single calculation, where the green parts denote the numbers of cooperators, presenting a significant power-law distribution pattern. Figure 8b shows the overall statistical results. Under either weak selection or strong selection, $f_{C} (k)$ is a monotonically decreasing function of $k$. Especially under weak selection, $f_{C} (2)\left| {_{\omega = 0.1} } \right. \approx 0.52$ and $f_{C} (12)\left| {_{\omega = 0.1} } \right. \approx 0.35$. In addition, with the further increase in $k$, $f_{C} (k)$ drastically declines.

#### Conclusion 3

Most (in the tiny minority) nodes with a high degree of connectivity on the community network hold strategy *D*, and many nodes with a low degree of connectivity are more likely to be occupied by individuals selecting strategy *C*.

Nodes with a high degree were able to select strategy *D* “at will” because there was a high probability that they would find a neighbour holding strategy *C* for “exploitation”. However, a low-degree node was not able to be so empowered because it was connected to a high-degree individual holding strategy *D*, and the probability of finding a neighbour holding strategy *C* was extremely small. Hence, strategy *C* was its best choice, which could at least prevent its own payoff from being zero. In summary, a large number of low-degree nodes were occupied by individuals holding strategy *C*, and all high-degree nodes became free riders by exploiting neighbouring individuals holding strategy *C*.

#### Insight 1

Based on the above Conclusions 1–3, in unmanned swarm operations, the swarm scale should be reasonably determined based on specific military requirements, such as combat tasks and combat styles. A small swarm scale can reduce the difficulty of equipment support, but may restrict the exertion of combat effectiveness. A large swarm scale can occupy advantages in attack and defense, but has high requirements for equipment support. Therefore, the determination of swarm scale is necessary to strike a balance between equipment investment cost and combat effectiveness; during the construction of swarm network, the number of network links should be reduced as much as possible on the premise of ensuring the core connectivity requirements. On the one hand, it ensures the level of cooperation within the swarm, on the other hand, it reduces the difficulty of our network construction and improves the difficulty of enemy communication interference; If the battlefield electromagnetic and communication conditions permit, the swarm can be controlled mainly by swarm self-organization and supplemented by human forced intervention. The human intervention focuses on the strategy selection of hub nodes (generous nodes) in the swarm network to improve the overall cooperation level.

### Influence of inner-community attachment and inter-community attachment on collaboration

**Figure 9:**
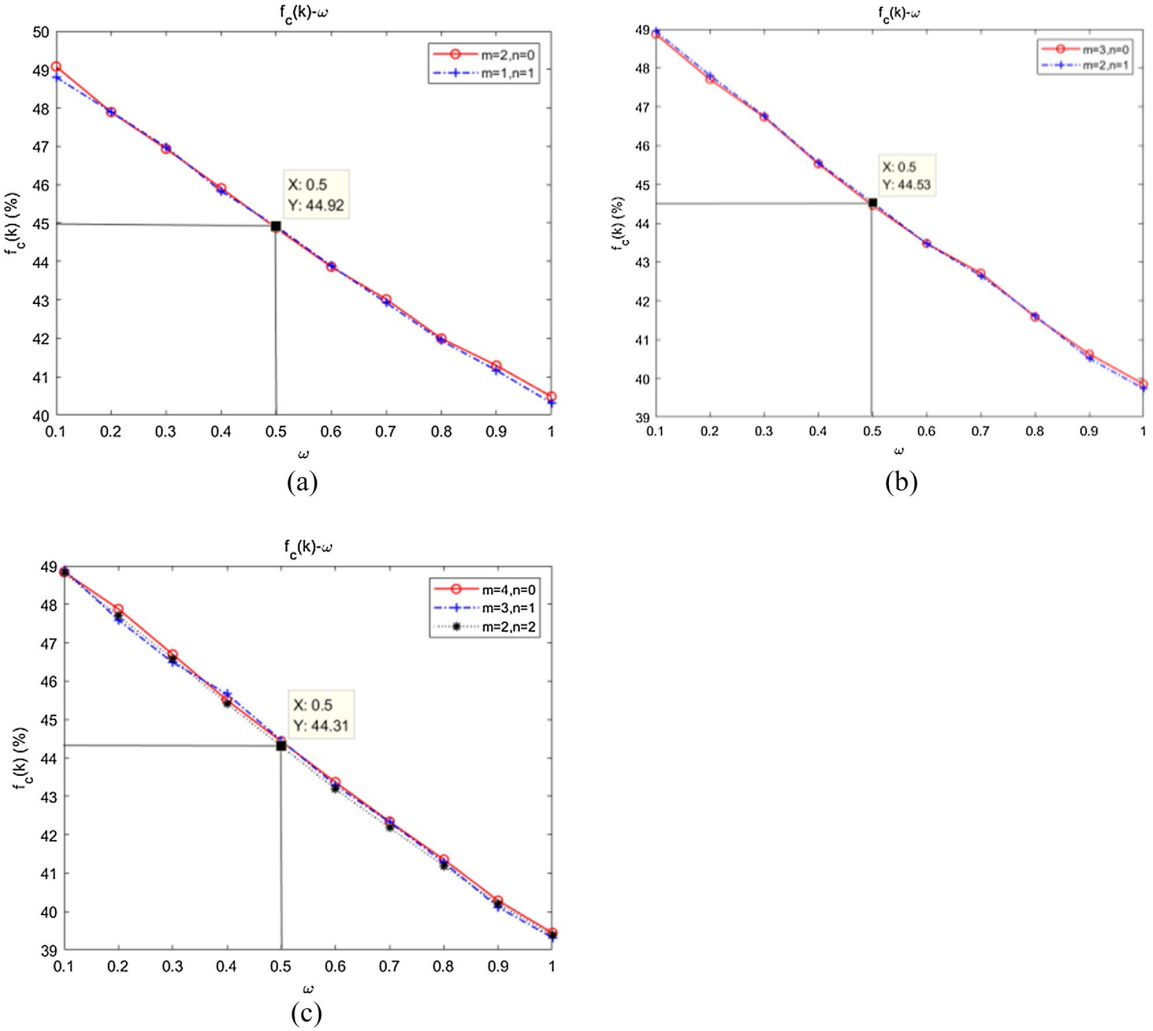
Dependence of the emergence of collaboration on intra-community attachment and inter-community attachment. (**a**) $\overline{k} \approx 4$; (**b**) $\overline{k} \approx 6$; (**c**) $\overline{k} \approx 8$.

In the construction of a swarm network, in addition to the parameter of attention distribution, the relative relationship between internal connections and external connections is also an important consideration because even if the two networks have the same average degree, if the relative relationship between internal/external connections is different, they will show different network characteristics. Based on the obtained pattern of influence of network degree on swarm collaboration, we further partitioned network degree and analysed the relationship between the relative degrees of intra-community attachment and inter-community attachment on the level of collaboration. Figure 9 shows the simulation results with $M = 5$ and $N = 100$.

As shown in Fig. 9a, $f_{C} (\overline{k} \approx 4)\left| {_{{m = 2,{\kern 1pt} {\kern 1pt} {\kern 1pt} n = 0,{\kern 1pt} {\kern 1pt} \omega \in [0.1,{\kern 1pt} {\kern 1pt} {\kern 1pt} {\kern 1pt} 1]}} } \right. \approx f_{C} (\overline{k} \approx 4)\left| {_{{m = 1,{\kern 1pt} {\kern 1pt} {\kern 1pt} n = 1,{\kern 1pt} {\kern 1pt} {\kern 1pt} \omega \in [0.1,{\kern 1pt} {\kern 1pt} {\kern 1pt} {\kern 1pt} 1]{\kern 1pt} }} } \right.$. In Fig. 9b, $f_{C} (\overline{k} \approx 6)\left| {_{{m = 3,{\kern 1pt} {\kern 1pt} {\kern 1pt} n = 0,{\kern 1pt} {\kern 1pt} \omega \in [0.1,{\kern 1pt} {\kern 1pt} {\kern 1pt} {\kern 1pt} 1]}} } \right. \approx f_{C} (\overline{k} \approx 6)\left| {_{{m = 2,{\kern 1pt} {\kern 1pt} {\kern 1pt} n = 1,{\kern 1pt} {\kern 1pt} {\kern 1pt} \omega \in [0.1,{\kern 1pt} {\kern 1pt} {\kern 1pt} {\kern 1pt} 1]{\kern 1pt} }} } \right.$, and in Fig. 9c, $f_{C} (\overline{k} \approx 8)\left| {_{{m = 4,{\kern 1pt} {\kern 1pt} {\kern 1pt} n = 0,{\kern 1pt} {\kern 1pt} \omega \in [0.1,{\kern 1pt} {\kern 1pt} {\kern 1pt} {\kern 1pt} 1]}} } \right. \approx f_{C} (\overline{k} \approx 8)\left| {_{{m = 3,{\kern 1pt} {\kern 1pt} {\kern 1pt} n = 1,{\kern 1pt} {\kern 1pt} {\kern 1pt} \omega \in [0.1,{\kern 1pt} {\kern 1pt} {\kern 1pt} {\kern 1pt} 1]{\kern 1pt} }} } \right.$. Therefore, with the average degree $\overline{k}$ remaining constant, the collaboration level was not significantly strengthened or weakened by increasing or decreasing intra-community attachment $m$ or inter-community attachment $n$, that is, by increasing or reducing the value of ${m \mathord{\left/ {\vphantom {m n}} \right. \kern-\nulldelimiterspace} n}$. By horizontally comparing Fig. 9a–c, it was found that the collaborative level weakened with increasing $\overline{k}$ (when $\omega = 0.5$, $f_{C} (4) \approx 0.4492 > f_{C} (6) \approx 0.4453 > f_{C} (8) \approx 0.4431$), which also verified the results in Fig. 7.

#### Conclusion 4

With a constant average degree of community network connectivity, changes in the relative degrees of intra-community attachment and inter-community attachment will not affect collaboration.

**Figure 10:**
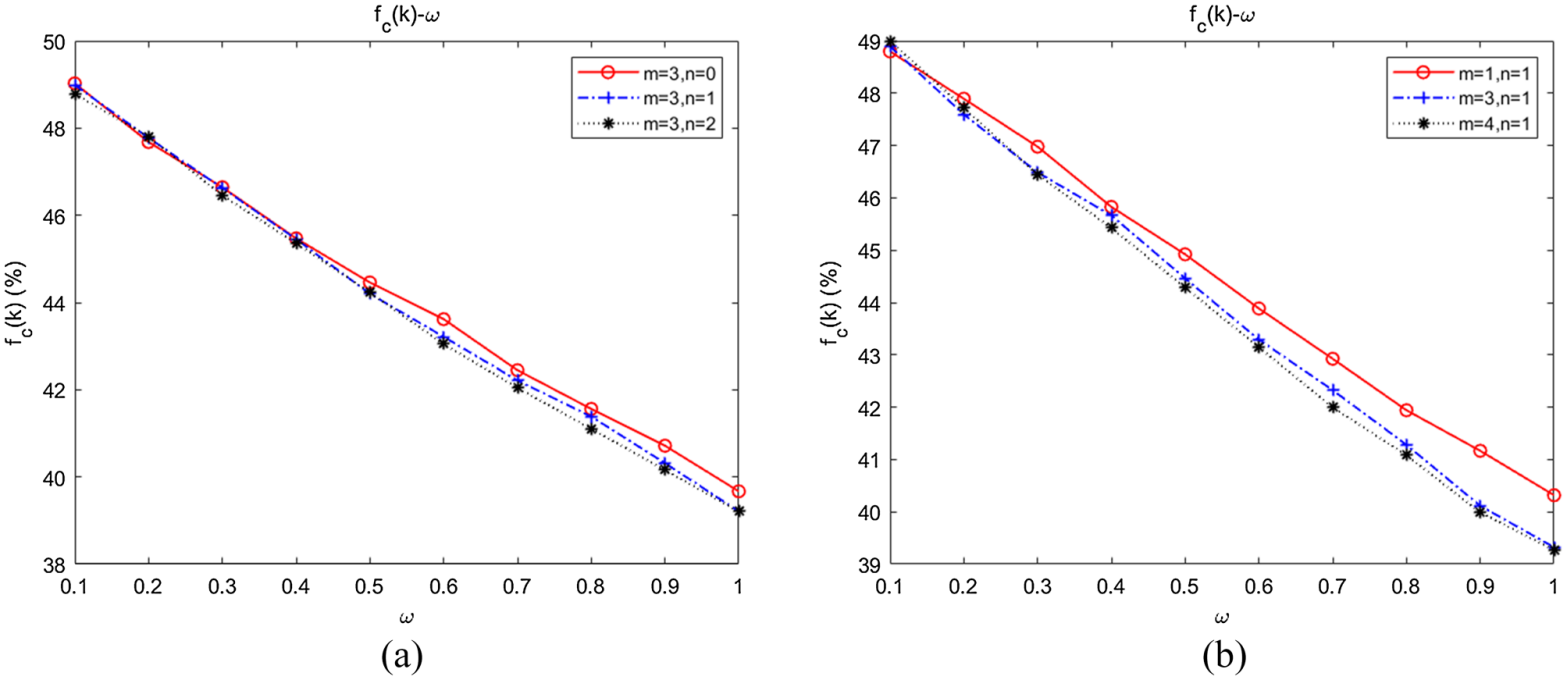
Dependence of the emergence of collaboration on the relative degrees of *m* and *n*. (**a**) The influence of fixed *n*, *m* on collaboration; (**b**) the influence of fixed *m*, *n* on collaboration.

With fixed $m$ and $n$, we further simulated the influence of changes in $m$ and $n$ on collaboration, the results of which are shown in Fig. 10a,b, respectively.

When $m = 3$, we fitted the average abundance curves of the swarms when $n = 0,1,2$. As shown in Fig. 10a, $f_{C} (k)\left| {_{{m = 3,{\kern 1pt} {\kern 1pt} n = 0}} } \right. > f_{C} (k)\left| {_{{m = 3,{\kern 1pt} {\kern 1pt} n = 1}} } \right. > f_{C} (k)\left| {_{{m = 3,{\kern 1pt} {\kern 1pt} n = 2}} } \right.$, which indicated that collaboration would be inhibited by increasing inter-community attachment when intra-community attachment remained constant. When $n = 1$, the average abundance curves of the swarms when $m = 1,3,4$ were fitted. As shown in Fig. 10b, $f_{C} (k)\left| {_{{n = 1,{\kern 1pt} {\kern 1pt} m = 1}} } \right. > f_{C} (k)\left| {_{{n = 1,{\kern 1pt} {\kern 1pt} m = 3}} } \right. > f_{C} (k)\left| {_{{n = 1,{\kern 1pt} {\kern 1pt} m = 4}} } \right.$, implying that collaboration would be restrained as well by strengthening intra-community attachment with inter-community attachment remaining unchanged.

#### Conclusion 5

Collaboration in unmanned swarms on community networks will be weakened by either increasing inter-community attachment with intra-community attachment remaining constant or increasing intra-community attachment with inter-community attachment unchanged.

Network heterogeneity and the direct connectivity between high-degree nodes are the core driving forces of collaboration^103^. Hence, the reasons for the results described above were qualitatively analysed from the following three aspects.

1. According to the data analysis/simulation, when the average degree of network connectivity was low, the initial fixed node was always the *hub* node^98^ with the greatest degree in its community, while as *m* or *n* increased ($\overline{k}$ increased), other nodes would compete for the *hub* position, which weakened the heterogeneity of the network to some extent.
2. With increasing *m* or *n,* the node would have a greater number of neighbours, but the uncollaborative free riders among the neighbours would also increase at the same time, thereby weakening the emergence of collaboration.
3. Given a fixed average degree $\overline{k}$, the best structure was that in which five scale-free networks were connected through five inter-community links (that is, *n* = 0), and all new nodes were only attached within the community. This contributed to the formation of loop structures, which have been demonstrated to greatly facilitate collaboration^53^.

#### Insight 2

Based on Conclusions 4–5, based on the existing swarm network, according to the task and the requirements for the flexibility, survivability, and robustness of the combat network, the communication relationship and network structure inside and outside the community should be optimized on the premise of keeping the average degree of the network unchanged as far as possible. In the operational stage dominated by a single operational cluster (such as intelligence investigation, firepower strike), the communication links between clusters can be reduced and the internal communication connections of cluster can be strengthened. In the operational stage requiring the collaborative participation of multiple clusters, the internal communication links of cluster can be reduced and the communication connections between cluster can be guaranteed.At the same time, on the premise of ensuring the core connectivity requirements, the number of internal or external links of the community network should be reduced as much as possible to promote the emergence of cooperative behaviour.

### Dependence of collaboration on cost and the multiplication factor

It is of strong practical guiding value to explore the influence of operational costs (e.g., communications, intelligence, and firepower) and the multiplication factor on collaboration in unmanned swarms. In this section, we combine theoretical derivation with data simulation to explore how the level of collaboration varies with two types of parameters to provide decision support for actual combat.

First, we examined the impact of cost on the collaborative level. In real combat, we seek to exchange the lowest cost investment for the optimal swarm coordination effect and finally achieve the maximum combat effectiveness. In contrast, if the cost is too high, we will fail to achieve the combat purpose, in which “the gain outweighs the loss”. Generally, let $c = 1.0$ represent the total amount of basic resources, and the general value range of $c$ is $c \in [1.0,{\kern 1pt} {\kern 1pt} {\kern 1pt} {\kern 1pt} 2.0]$. When $c = {\kern 1pt} 2.0$, the actual resource consumption is doubled. If $c > {\kern 1pt} 2.0$, from a practical point of view, the cost of investing resources is too high, which has lost its operational relevance. Therefore, we make $c \in [1.0,{\kern 1pt} {\kern 1pt} {\kern 1pt} {\kern 1pt} 2.5]$, which not only covers the general value space but also considers the unexpected situation.

**Figure 11:**
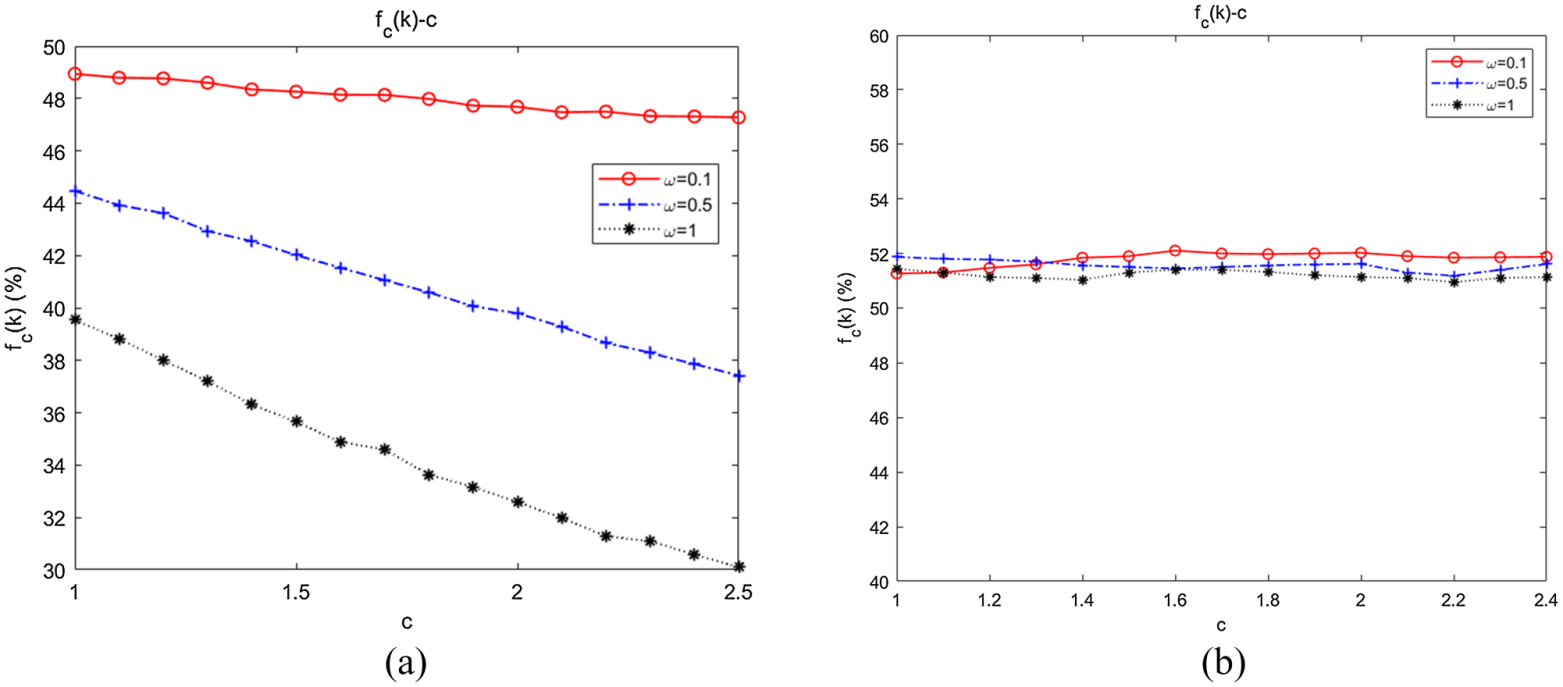
Relationship between average abundance and cost. (**a**) Defection strategy dominates; (**b**) collaboration strategy dominates.

Figure 11 shows how the collaborative level $f_{C}$ varies with $c$ when $m = 2$ and ${\kern 1pt} n = 1$.

As shown in Fig. 11a, the initial condition was that the defection strategy dominated ($f_{C} < 0.5$). With increasing cost $c$, $f_{C}$ monotonically decreased. The simulation results also verified the theoretical derivation mentioned above. Since $a_{{k_{i}^{C} }} = b_{{k_{i}^{C} }} + [{r \mathord{\left/ {\vphantom {r {(\overline{k} + 1)}}} \right. \kern-\nulldelimiterspace} {(\overline{k} + 1)}} - 1] \cdot c$, when the defection strategy dominated, $a_{{k_{i}^{C} }} < b_{{k_{i}^{C} }}$; that is, $[{r \mathord{\left/ {\vphantom {r {(\overline{k} + 1)}}} \right. \kern-\nulldelimiterspace} {(\overline{k} + 1)}} - 1] \cdot c < 0$. Therefore, with increasing $c$, $a_{{k_{i}^{C} }}$ would be further decreased, and collaboration would be further inhibited. Given the same cost, $f_{C} (k)\left| {_{\omega = 0.1} } \right. > f_{C} (k)\left| {_{\omega = 0.5} } \right. > f_{C} (k)\left| {_{\omega = 1} } \right.$. Hence, the smaller $\omega$ is, the greater the average abundance $f_{C}$. In addition, as $\omega$ increases, the influence of $c$ on $f_{C}$ strengthens: $\Delta < f_{C} (\omega = 0.1) > \left| {_{{c \in [1.0,{\kern 1pt} {\kern 1pt} {\kern 1pt} {\kern 1pt} 2.5]}} } \right. \approx 0.020$, $\Delta < f_{C} (\omega = 1) > \left| {_{{c \in [1.0,{\kern 1pt} {\kern 1pt} {\kern 1pt} {\kern 1pt} 2.5]}} } \right. \approx 0.094$.

In Fig. 11b, the collaboration strategy was dominant ($f_{C} > 0.5$). Based on Eqs. (17) and (18), when the collaboration strategy dominates, increasing $c$ increases $a_{{k_{i}^{C} }}$, but the benefits to defectors $b_{{k_{i}^{C} }}$ increase as well. Since $[{r \mathord{\left/ {\vphantom {r {(\overline{k} + 1)}}} \right. \kern-\nulldelimiterspace} {(\overline{k} + 1)}} - 1] \cdot c > 0$, $a_{{k_{i}^{C} }}$ increases more significantly than $b_{{k_{i}^{C} }}$; that is, increasing $c$ promotes collaboration.

However, at the same time, we should pay attention to the collateral effect which means that when an individual changes strategies, the most direct impact is the change that will bring his own benefits, but in addition, it will affect the strategy choice of his neighbours. For a *D* strategy holder $i_{D}$, it can be seen from Eq. (18) that for each additional cooperator $j_{C}$ among its neighbours, it can obtain the benefit ${{rc} \mathord{\left/ {\vphantom {{rc} {(k + 1)}}} \right. \kern-\nulldelimiterspace} {(k + 1)}}$ by “exploiting” the cooperator, and ${{rc} \mathord{\left/ {\vphantom {{rc} {(k + 1)}}} \right. \kern-\nulldelimiterspace} {(k + 1)}}$ is greater than the benefit increment $[{r \mathord{\left/ {\vphantom {r {(k + 1)}}} \right. \kern-\nulldelimiterspace} {(k + 1)}} - 1] \cdot c$ brought by strategy conversion of $i_{D}$ (from strategy *D* to strategy *C*). Therefore, $i_{D}$ is more inclined to maintain the existing *D* strategy instead of converting to strategy *C*. In other words, the strategy transformation of $j_{C}$ (from strategy *D* to strategy *C*) has a collateral effect on the strategy choice of $i_{D}$, so that $i_{D}$, who was supposed to change to strategy *C*, abandons the strategy transformation and adheres to strategy *D*. Therefore, the collateral effect inhibits cooperation; this is, in Fig. 11b, the increase in cost will basically maintain the cooperation level near the initial value.

#### Conclusion 6

When the defection strategy dominates, increasing cost will decrease the level of collaboration, especially when the selection intensity is relatively high; when the collaboration strategy dominates, the increase in cost will basically maintain the cooperation level near the initial value.

**Figure 12:**
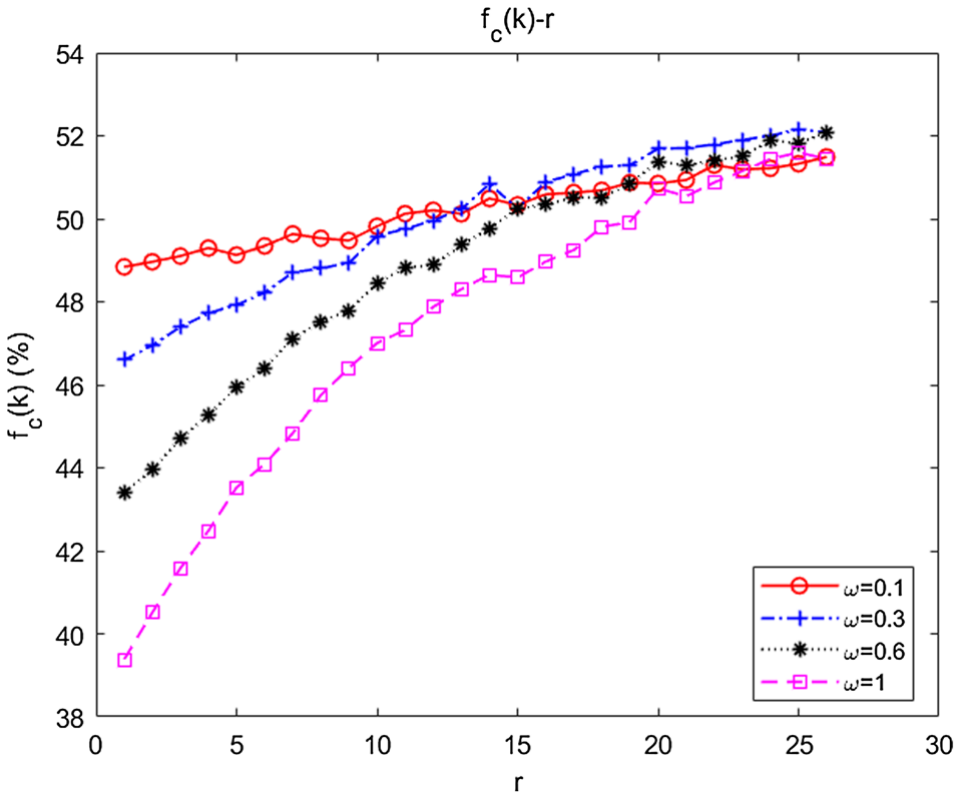
Relationship between average abundance and the multiplication factor.

The multiplication factor $r$ determines the “appreciation rate” of individual resources. The appreciation of resources is reflected in the overall efficiency of “1 + 1 > 2” brought by swarm coordination. An excessively small multiplication factor cannot promote the transformation of an unmanned platform to a cooperation strategy, and an excessively large multiplication factor has no practical relevance. Figure 12 shows the average abundance with respect to the multiplication factor when $r \in [1,{\kern 1pt} {\kern 1pt} {\kern 1pt} {\kern 1pt} 6]$, $m = 2$, and $n = 1$.

As shown in Fig. 12, with the increase in the multiplication factor $r$, $f_{C}$ increases monotonically, indicating that due to the increased multiplication factor, a large number of collaborative behaviours emerge in the swarms, and the “free-riding” behaviour is inhibited; nevertheless, as $r$ increases, the influence of $r$ on the promotion of $f_{C}$ gradually declines. When $\omega = 1$, $\Delta < f_{C} (k) > \left| {_{{{\kern 1pt} r \in [1,{\kern 1pt} {\kern 1pt} {\kern 1pt} {\kern 1pt} 10]}} } \right. \approx 0.074$, and $\Delta < f_{C} (k) > \left| {_{{r \in [10,{\kern 1pt} {\kern 1pt} {\kern 1pt} {\kern 1pt} 25]}} } \right. \approx 0.041$.

#### Conclusion 7

An increase in the multiplication factor contributes to the emergence of collaboration, but the greater the multiplication factor is, the less significantly it promotes collaboration.

**Figure 13:**
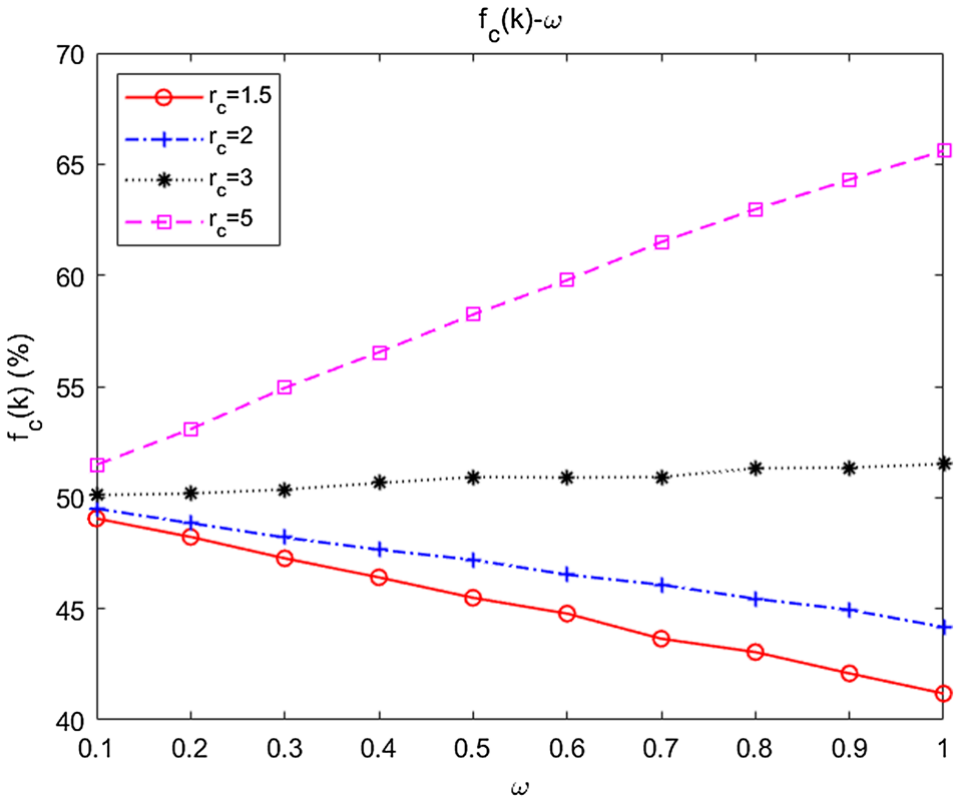
Relationship between average abundance and intensity under different values of $r_{C}$.

However, the means of increasing the level of collaboration by adjusting the multiplication factor is of only theoretical value but not of practical relevance when the multiplication factor is too high ($r > 5$). As stated in Conclusion 7, the greater the multiplication factor is, the less significantly it promotes collaboration because increasing the multiplication factor increases the payoffs of both cooperators and defectors. Therefore, we tried to separate the multiplication factor $r_{C}$ of cooperators from the multiplication factor $r_{D}$ of defectors. By only increasing $r_{C}$($r_{D}$ remained unchanged), we fitted the curves showing the effects of varying $r_{C}$ on average abundance (Fig. 13). When $r_{C} { = }3$, the average abundance is approximately equal to 0.5 (as shown by the black curve in the figure), which indicates that the proportions of cooperators and defectors in the swarms are basically balanced. As $r_{C}$ further increases, when $r_{C} { = }5$, the average abundance is greater than 0.5 at $\omega = 0.1$, and then as the selection intensity becomes stronger, the average abundance exceeds 0.65, which is a substantial increase. Moreover, as $r_{C}$ increases, when the dominant strategy changes, strong selection would be more conducive to the emergence of collaboration.

The increase in the multiplication factor $r_{C}$ meant that free riders who adopted defection strategies would no longer gain the same payoffs as the cooperators. The decrease in payoff would directly increase the probability of defectors updating their strategies $P_{D \to C}$, thereby causing more units to collaborate.

#### Conclusion 8

With the multiplication factor of cooperators separated from that of defectors, increasing the multiplication factor of cooperators alone can greatly increase the average abundance of the swarms; when the collaboration strategy dominates, in contrast to the traditional model with “collaboration facilitated by weak selection”^89^, strong selection will be more conducive to the emergence of collaboration.

**Figure 14:**
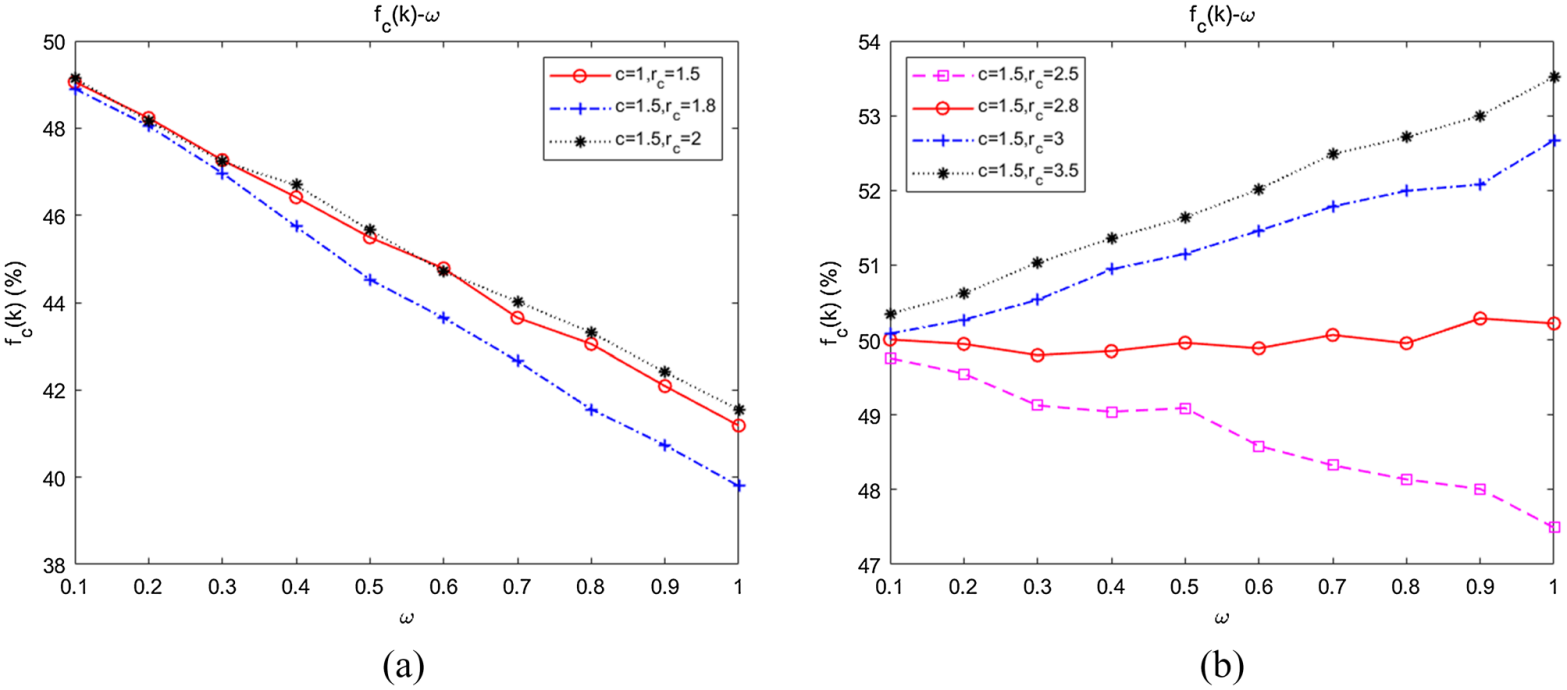
(**a**) Effects of cost and the multiplication factor on average abundance when the defection strategy dominates; (**b**) effects of cost and the multiplication factor on average abundance when the collaboration strategy dominates.

The ideal scenario in actual control is to simultaneously increase the multiplication factor $r_{C}$ and reduce the costs to cooperators. However, for specific missions on the battlefield, to ensure the effectiveness of combat, the cost will increase rather than decrease. Nevertheless, according to Conclusion 6, when the defection strategy dominates, the increase in cost will reduce the collaborative level, which counteracts the increase in $r_{C}$. Hence, it is necessary to comprehensively consider the situation where $r_{C}$ and $c$ increase at the same time. Figure 14a shows the variation in the collaborative level with $r_{C}$ and $c$ increasing at the same time when the defection strategy dominates. With $c = 1$ and $r_{C} = 1.5$ as the benchmark (as shown by the red data points), when the cost rises by 50% (from $c = 1$ to $c = 1.5$) and the multiplication factor increases by 33% from $r_{C} = 1.5$ to $r_{C} = 2$ (as shown by the black data points), the negative effects of $c$ on collaboration are offset, and the emergence of swarm collaboration can also be promoted.

According to Conclusion 6, when the collaboration strategy dominates, increasing cost improves the level of collaboration, but not very significantly. Here, $r_{C}$ and $r_{D}$ are regulated and controlled separately. Only $r_{C}$ increases ($r_{D}$ remains constant). As shown in Fig. 14b, the collaborative level is tremendously improved when the multiplication factor increases to 3 and the cost to 1.5 (as shown by the blue data points), which is inconsistent with Conclusion 6. As c increases, the payoffs to cooperators and defectors increase at the same time. However, since $r_{C} > r_{D}$, based on Eqs. (17) and (18), ${{r_{C} {\kern 1pt} {\kern 1pt} k_{c} {\kern 1pt} {\kern 1pt} c} \mathord{\left/ {\vphantom {{r_{C} {\kern 1pt} {\kern 1pt} k_{c} {\kern 1pt} {\kern 1pt} c} {k + 1}}} \right. \kern-\nulldelimiterspace} {k + 1}} > {{r_{D} {\kern 1pt} {\kern 1pt} k_{c} {\kern 1pt} {\kern 1pt} c} \mathord{\left/ {\vphantom {{r_{D} {\kern 1pt} {\kern 1pt} k_{c} {\kern 1pt} {\kern 1pt} c} {k + 1}}} \right. \kern-\nulldelimiterspace} {k + 1}}$, and the cooperators gain an extra portion of the payoff $({{r_{C} } \mathord{\left/ {\vphantom {{r_{C} } {k + 1}}} \right. \kern-\nulldelimiterspace} {k + 1}} - 1){\kern 1pt} {\kern 1pt} c$. Therefore, the emergence of collaborative behaviour was apparently improved over that in Fig. 11b.

#### Conclusion 9

When the defection strategy dominates, regulation of $r_{C}$ not only offsets the negative influence of cost on collaboration but also contributes to the emergence of collaborative behaviours; when the collaboration strategy dominates, comprehensive regulation of $r_{C}$ and $c$ can remarkably improve the collaboration level.

#### Insight 3

Based on simulation Conclusions 6–9, in the actual control, the multiplication factor of cooperators in the unmanned swarm should be increased as much as possible and the multiplication factor of non-cooperators should be restrained. For example, with the help of management means, for each combat unit of the swarm, the investment cost (such as the total amount of bombs dropped) of its previous operations can be accumulated. In the subsequent operations, those with higher investment cost shall be given more material and ammunition supply, or higher priority of material and ammunition supply; Under the initial conditions dominated by non-cooperators in the swarm, reduce, at least maintain the cost of a single operation as much as possible. For example, with the help of advanced technology, improve the reliability and survivability of the combat platform, and improve the strike accuracy and damage power of unit ammunition.

The above simulation was completed based on the NetLogo 6.1.1 platform. The images and videos in the “Supporting Materials” show the configuration of the platform interface, the generation of the initial network, and the process of the evolutionary game.

## Conclusion

The greatest advantage of an unmanned swarm lies in the autonomous collaboration among its units. When manual control fails because a single platform is damaged or communication is blocked, the unmanned swarm is still able to operate in an orderly manner. In view of the collaborative evolution of unmanned swarms, we analysed relevant military demands in this study and reviewed pioneering studies worldwide. On this basis, a dynamic evolutionary game model was established to simulate and analyse the influence of swarm size, network connectivity degree distribution, cost, and the multiplication factor on collaborative behaviours in unmanned swarms. In addition, we proposed reasonable suggestions for coordinated management and control in swarm combat. The conclusions reached enable related theories to be applied in practical applications.

In this study, the information network was assumed to be static. However, limited communication conditions and the destruction of nodes may lead to the dynamic reconstruction of the network topology in actual combat. The follow-up study will focus on how to investigate the coevolution of strategy and structure and explore their collaborative evolutionary mechanisms under dynamic topology.

## Supplementary Information

Supplementary Information.
