# Supplementary material for: A game-based approach for designing a collaborative evolution mechanism for unmanned swarms on community networks
Source: Sci Rep. 2022 Nov 7;12:18892. doi: 10.1038/s41598-022-22365-z (PMC9640601; doi:10.1038/s41598-022-22365-z)
Supplement: Supplementary file 1 — Supplementary Information. [file 41598_2022_22365_MOESM1_ESM.zip › Supporting material/2.í╢An Approach to Coordinated Control of Structured Unmanned Swarms based on Evolutionary Gameí╖.pdf]

An Approach to Coordinated Control of Structured Unmanned Swarm based on Evolutionary Game

1st Minggang Yu
Institute of Command and Control Engineering; Institute of Communication Engineering
Army Engineering University of PLA
Nanjing, China
0000-0002-2732-3219
yuminggang8989@163.com

2st Ming He
Institute of Command and Control Engineering
Army Engineering University of PLA
Nanjing, China
0000-0002-1127-9073

3st Dongge Zhang
Institute of Command and Control Engineering
Army Engineering University of PLA
Nanjing, China
DongGeZhang@hotmail.com

4st Ling Luo
Institute of Command and Control Engineering
Army Engineering University of PLA
Nanjing, China
luoling@163.com

5st Jintao Liu
Institute of Command and Control Engineering
Army Engineering University of PLA
Nanjing, China
liu_jintao@126.com

6st Legui Zhang
Institute of Command and Control Engineering
Army Engineering University of PLA
Nanjing, China
329674406@qq.com

***Abstract*—To solve the problem of autonomous collaboration of unmanned cluster in military operations, an approach to coordinated control of structured unmanned swarm based on evolutionary game is proposed. It starts with the requirement analysis of task collaboration in unmanned cluster; and the collaboration model based on evolutionary game theory is established; Then the evolutionary dynamics and equilibrium is constructed by theoretical derivation, furthermore, the formation conditions of autonomous collaboration is obtained; Finally the strategy evolution process of swarm on regular lattice and general topology is simulated via case study, which proves the rationality and feasibility of the proposed model and method. The conclusion of this paper provides a preliminary and meaningful exploration for the transformation from theories to practical applications.**

***Keywords*—unmanned swarm, coordinated control, evolutionary game, evolutionary dynamics**

I. Introduction

With the continuous advancing of the third wave of artificial intelligence, "group evolutionary intelligence" developed from "single agent autonomous intelligence" has become one of the important characteristics of the new generation of artificial intelligence. Especially in the military field, unmanned swarm (unmanned vehicle cluster[1-3], unmanned boat cluster[4-6], unmanned aerial vehicle cluster[7-10]) operations have received unprecedented attention over the past two years. The U.S. military has listed unmanned swarm operations as a "subversive technology" that can change the rules of war.

There are mainly two kinds of control modes of unmanned swarm: centralized control and autonomous collaboration. In the complex electromagnetic environment of the battlefield, especially when the swarm enters the depth of the enemy's position, the communication failure is a common real risk[11]. In such cases, the centralized control mode fails, and the unmanned swarm must make effective response on the spot according to the external situation and achieve self-management and self-coordination, so as to complete the established military operations.

The autonomous collaboration of unmanned swarm requires cooperation between unmanned units with different functions. For example, in the fire attack task, the cooperation between the logistic unit and the fire unit is needed; in the intelligence reconnaissance task, the cooperation between the intelligence unit and the information fusion unit is essential. In fact, the phenomenon of division of labor and cooperation has existed in the biological world for a long time. For example, when the lions cooperate in hunting, they will spontaneously take different actions. Generally, two lions will attack a group of prey from both wings, making them run forward because of panic. At the same time, another one or two lions will rush to the middle to attack the prey. This hunting method is very effective. The characteristic of the above examples are that for a common task, it can only be completed through the cooperation of two different roles, and no single individual can complete the task independently. When dealing with such coordination control problems, an important task is how to planning strategy distribution on the connection network topology, so that individuals can be evenly distributed around the individuals with complementary strategies. For example, in the task of fire attack, it is necessary to make the units with complementary functions (ammunition support and fire attack) distributed as close as possible, so that the neighbors can complement each other and complete the task efficiently.

Evolutionary game theory[12-14] provides an effective mathematical tool and research framework for the study of interaction and cooperation among multiple individuals. Evolutionary game theory combines "equilibrium" in economics with "adaptability" in biology to depict the process that individuals adapt to the external environment through learning or imitation under boundary rationality and asymmetric information, and finally tend to an evolutionarily stable state (ESS)[15]. In recent years, many

Fund support：National Natural Science Foundation of China (71901217)；National Social Science Foundation（19BXW117）；National Key R&D Program of China（2018YFC0806900）；China Postdoctoral Science Foundation funded Project（2018M633757）

978-1-7281-8025-0/20/$31.00 ©2020 IEEE

Authorized licensed use limited to: HUNAN UNIVERSITY. Downloaded on January 13,2021 at 13:39:37 UTC from IEEE Xplore. Restrictions apply.

scholars use evolutionary game theory to study the mechanism of cooperation promotion. Professor Nowak of Harvard University, as a representative, expanded the "comprehensive evolution theory" and summarized five rules[16] to promote the emergence of cooperation. Evolutionary game theory provides an important mathematical framework for the study of cooperative control.

In the issue of division and cooperation of the unmanned swarm, the control goal is to make adjacent units in complementary strategy state, so as to complete the cooperation task more efficiently. Therefore, when a unit plays games with its neighbors, if they hold different strategies, the gains should be higher than holding the same strategy. Therefore, when controlling the evolution of swarm, we need to choose the game type, whose Nash equilibrium is to take the opposite strategy to its neighbor for each unit, in such a situation, the whole swarm can obtain higher benefits. Snowdrift game (or Hawk-dove game, Chicken game) [17] exactly meets the above requirements. When the above games are launched among the unmanned units in the swarm, the evolutionary stability strategy is to take the opposite strategy with each other, so this kind of model provides a good theoretical framework for analyzing the division and cooperation of unmanned swarm.

At present, there are two main research directions to solve the problem of division and cooperation with evolutionary game theory: one is to study the evolutionary stability state of well-mixed population and the dominant condition of cooperation based on Markov stochastic process[18-19], and the other is to study the evolutionary dynamics process and cooperation mechanism of spatial structured population such as complex network based on graph theory[20-21].

For the former, the representative researches are as follows: the team of Professor Du Jinming from Peking University obtained the dominant conditions of two-party game strategies[22] and the influence of threshold value on the formation of multi-party snowdrift game cooperation under the aspiration driven dynamics and well-mixed population through theoretical derivation and simulation. Furthermore, the team extended the theoretical results to the double threshold value and deduced the ratio of benefit to cost (*b/c*) of snowdrift game under the double threshold[24-25]. In addition, there are also researchers who have studied the mechanism of punishment to promote the evolution of cooperation in snowdrift game[26]. However, the "well-mixed" assumes the full connection between individuals of population, without considering the impact of structure on cooperation in swarm. In the real battlefield environment, unmanned platforms form a specific network structure through physical/information links. Therefore, how to analyze the evolution mechanism of swarm cooperation based on a specific network structure is an inevitable way to achieve cluster cooperation control.

For the latter, the team of Professor Nowak from Harvard University theoretically deduced the evolution of population in spatial structure such as circle, random graph and scale-free network, and creatively proposed the relationship between the ratio *b/c* and the network average degree *k*. They pointed out that the smaller the network connectivity is, the more conducive the cooperation in natural selection is[27]. Then, they use the pair approximation theory to theoretically deduce the cooperation phenomenon on the regular lattice, and obtain the boundary conditions for the generation and expansion of cooperation[20, 28]. On the basis of the above achievements, further comparative analysis is made on the differences between homogeneous and heterogeneous networks in promoting cooperative behavior, and simulation results show that weak connection can better promote cooperative behavior on heterogeneous networks[29]. At the same time, other researchers study the dynamic process of multi-party game on the graph, and simulation results show that spatial structured population can promote the occurrence of cooperation better compared with unstructured population[21]. In recent two years, the team of Nowak has applied the evolution dynamics of cooperation in spatial structure to social network, analyzed the critical conditions of cooperation behavior in human society[30], initially explored the trade-off between the evolution convergence probability and the evolution convergence time[31], and extend the cooperative evolution on structural population to weighted graph[32]. Other representative studies include literature[33]-[34] on the specific model of multiplayer snowdrift game, the relationship curves between the ratio *b/c* and cooperation level on the well-mixed population and the structured population is given respectively, and the significant differences between the homogeneous/heterogeneous network and the unstructured population in the promotion of cooperation is compared.

The above achievements on cooperation have high theoretical and application value. However, there are still two shortcomings when solving the problem of coordinated control of unmanned swarm. First, the existing achievements focus on the critical conditions for the generation of cooperation behavior, however, the issue of coordinated control which is to pursue the utility maximizing of swarm is not fully considered. Although the generation of cooperation behavior and the maximization of the overall utility of swarm are similar in mechanism[35], there are essential differences in the way of realization. Second, there are no achievements on military field. At present, we can see the application of snowdrift game (or Hawk-dove game, Chicken game) in environmental pollution[36], public opinion dissemination[37], cultural evolution [38], etc. Due to the particularity of the military field and the newly emerging of unmanned swarm operations, there are few results to follow.

In our previous work, we derived the average abundance function and dominant condition of the cooperative strategy in well-mixed swarm based on evolutionary game theory[39]. On this basis, this study will extend the hypothesis of well-mixed to the spatial structure. First, we model the issue of division and cooperation of the unmanned swarm based on evolutionary game. Then, we give the differential equation of evolutionary dynamics on the spatial structure, and theoretically deduce the equilibrium solution and approximate solution while considering subtle disturbance. Next, taking the fire strike scenario as an example, we simulate the cooperative evolution of swarm on regular lattice and general topology respectively. Finally, some reasonable suggestions are given to realize the coordinated control in the unmanned swarm operation based on the theoretical derivation and simulation results.

II Model Construction

Cooperation of unmanned swarm is a game process of

Authorized licensed use limited to: HUNAN UNIVERSITY. Downloaded on January 13,2021 at 13:39:37 UTC from IEEE Xplore. Restrictions apply.

multi-party and multi-round. Therefore, we use evolutionary game to model this issue. The mapping between the concepts of cooperation in unmanned swarm and evolutionary game is listed in TABLE I.

TABLE I MAPPING BETWEEN CONCEPTS

Cooperation in unmanned swarm	Evolutionary game
**Unmanned swarm**	Structured population
**Single combat unit**	Individual
**Single combat unit as research object**	Focal individual
**Corresponding behaviors displayed by a function unit (e.g., ammunition support)**	Strategy *A*
**Corresponding behaviors displayed by a function unit (e.g., fire attack)**	Strategy *B*
**Total rewards that units ultimately obtain after each round of game**	Pay-offs
**Pay-off based strategy transformation between units**	Game
**Dynamic change of proportion and strategy distribution of units in swarm in multi-round game**	Evolutionary
**The proportion and strategy distribution are stable and the game ends after multi-round game**	Evolutionary stable state

A. Game model of swarm evolution

In the evolutionary game model of unmanned swarm, a single unmanned unit acts as an individual player in the game. The swarm composed of multiple units has a common goal and all units have to complete the common goal. A single unit can choose different behaviors as its strategy. At each round game, one unit and its "neighbors" (other unmanned units with physical connection and logical connection with it) interact according to its own and its neighbors' strategies, and then it obtains certain benefits (payoff). The rational unit with independent decision-making capability updates its strategies according to its payoff and update mechanism. With the multi-round strategy updating, the swarm evolves continuously, which makes the control over swarm reach the target state (such as consistency, synchronization, cooperation, etc.). In the above process, the choice of game mode, the design of payoff calculation and the determination of strategy updating mechanism are the key elements, which determine the direction of swarm evolution and the realization of control objectives.

The evolutionary game takes place in a structure swarm with *N* scale. Each unmanned unit is defined as a quadruple:

$$\text{Unit}_i = \{\text{State}_i, \text{Transition}_i, \text{Interaction}_i, \text{Fitness}_i\}$$

i) State, the current strategy of the unmanned unit and the network connection with its neighbors. The state of swarm is defined as the proportion of units with various strategies, and the swarm structure.

ii) Transition, strategy adjustment based on payoff, and dynamic reconfiguration of topology. The transition of swarm is defined as the adjustment process of swarm state.

iii) Interaction, the process of information transmission and strategy game between unmanned units relied on topological structure.

iv) Fitness, the benefit from interaction between unit and its neighbors in each round of game, which reflect the adaptability of the unit to the environment(e.g., battlefield).

The interaction between units can be characterized by graph theory. Let $G=(N,L)$, where $i \in N$ is the node-set composed of unmanned unit and $L = N \times N$ is the edge set. Characterize directed edges with ordered pairs (i,j), and if unit i can receive the state information of unit j, then i is a neighbor of j. The neighbor set of i is defined as $k_i = \{j \in N | (i,j) \in L\}$, and if $(i,j) \in L$ then $(j,i) \in L$ when G is an undirected graph.

Each round of game is also called an evolution step $t(t \in \mathbb{Z})$. Unit i calculates its fitness according to the function $\{F_i(\cdot)\}_{i \in N}$, and updates its strategy accordingly. There are two common strategy update mechanisms: imitation dynamics and aspiration-driven dynamics[40]. For the former, the individual imitates the strategy of its neighbor owning higher payoff through pair-wise comparison[41], while for the latter, if the unit's own aspiration (expectation level) is not satisfied, the unit adjusts its strategy[42]. Aspiration-driven dynamics focuses on the comparison of its own payoff and aspiration level, and then makes a new round of decision, generally less considering the impact of environment. In addition, there are other strategy update mechanisms such as replicator dynamic[43] and Moran process[44]. The selection of strategy update mechanism needs to consider practical requirements, not only to achieve the goal of swarm control, but also to follow the practical constraints of the actual scene.

B. Mathematical description of swarm cooperation

Set swarm size as N and $\text{Unit}_i = \{S_i, T_i, I_i, F_i\}$. State space consists of two different strategies $S_i \in \{A, B\}$. Units implement strategy update (i.e. T_i) with a certain probability according to payoff. The dynamic adjustment of network topology in interaction is not considered in our work, so I_i is the preset static topology. Let $I_i = \{j \in N | c_{ij} = 1\}$, where c_{ij} is the element of adjacency matrix $C = \left[c_{ij}\right] \in R^{N \times N}$, and $c_{ij} = 1$ means that Unit_i can obtain the information of Unit_j. F_i is the benefit of game, which is not only the result of interaction I_i, but also the basis of transformation T_i.

In the issue of coordinated control of unmanned swarm, the goal is to make the adjacent units adopt different (i.e. complementary in function) strategies, which means that the payoff of holding the same strategy should be lower than holding different strategies respectively, when mapping to the game theory. Snowdrift game (or Hawk-dove game, Chicken game) just belong to this kind of game. The scenario described in snowdrift game[33] is that it snows heavily, the road is blocked by snowdrift, and two opposing drivers are trapped at both ends of the snowdrift at the same time. They have two strategies to choose from: cooperation or betrayal. Cooperation means getting out of the car to shovel snow, while betrayal means staying in the car and doing nothing, hoping that another person will shovel snow. If the two choose to cooperate, the workload will be halved for each person; if the two betray, they can only pass until the snow melts. If the benefit of smooth passing is b and the cost of snow shoveling is c, then the payoff matrix is as follows:

Authorized licensed use limited to: HUNAN UNIVERSITY. Downloaded on January 13,2021 at 13:39:37 UTC from IEEE Xplore. Restrictions apply.

$$\begin{matrix} & A & B \\ A & \begin{pmatrix} b-c/2 & b-c \\ b & 0 \end{pmatrix} \\ B & & \end{matrix} \quad (1)$$

According to the classic game theory, when $b > c$, the Nash equilibrium is to choose the opposite strategy: if the other party chooses to cooperate, it will choose to betray, if the other party chooses to betray, it will choose to cooperate. In fact, whether it's snowdrift game, hawk-dove game or chicken game, the premise for them to achieve the above equilibrium has the following common characteristics: $R < T$ and $S > P$, where R、T、S、P is the specific payoff in the general payoff matrix of game theory as shown below.

$$\begin{matrix} & A & B \\ A & \begin{pmatrix} R & S \\ T & P \end{pmatrix} \\ B & & \end{matrix} \quad (2)$$

If the payoff of Unit_i in the game with its neighbor Unit_j （$j \in I_i$） is F_{ij} and the total number of its neighbors is k_i, then its fitness can be defined as the mean value of all payoffs with its neighbors:

$$F_i = \frac{\sum_{j \in I_i} c_{ij} \cdot F_{ij}}{k_i} \quad (3)$$

The unmanned unit uses the imitation dynamic to update its strategy, that is to compare its own fitness with the average fitness of its neighbors（$\overline{F_j}$）, and updates the strategy in $\{A, B\}$ with a certain probability, which is as follows:

$$P_{S_i \in \{A,\, B\}} = \frac{1}{1 + e^{\omega(F_i - \overline{F_j})}}, \quad j \in I_i \quad (4)$$

where $\omega \in [0, 1]$ is the selection intensity, which can enlarge or reduce the influence of $F_i - \overline{F_j}$ on the strategy update probability, and the weak selection intensity （$\omega \ll 1$） can promote cooperation[45]. Let $\Delta = F_i - \overline{F_j}$, if Δ=0, then $P_{S_i \in \{A,\, B\}} = 1/2$, which means that units have the same preference for strategies *A* and *B*; if Δ>0(i.e., the pay-off F_i is higher than $\overline{F_j}$), then $P_{S_i \in \{A,\, B\}} < 1/2$, which means units are more inclined to maintain the current strategy; if Δ<0(i.e., pay-off F_i is lower than $\overline{F_j}$), then $P_{S_i \in \{A,\, B\}} > 1/2$, which means units prefer to update from the current strategy to another strategy in strategy space $\{A, B\}$.

III Theortical Analysis

In this part, we will analyze the control effect of the evolutionary game model on the coordinated control of unmanned swarm through strict theoretical derivation.

A. Dynamics of swarm evolution

In an N-scale unmanned swarm, each unmanned unit occupies a vertex of the network topology. In the initial state, each unit randomly selects one as the initial strategy in the strategy space $\{A, B\}$. Units play games with all their neighbors according to formula (2), and determines the average payoff of this round of game according to formula (3). We try to analyze the proportion change of units with strategy *A* and *B* in the whole swarm during the evolution process, find the evolution stable state, and then obtain the conditions for the formation of swarm cooperation.

P_A and P_B are defined as the proportion of units holding *A* and *B* strategies in the whole swarm. P_{AA}, P_{AB}, P_{BA} and P_{BB} are the proportion of strategy pair AA, AB, BA and BB, each of which is composed of two adjacent units. $q_{Y/X}$ is defined as the conditional probability of finding a unit with strategy Y （$Y \in \{A, B\}$） in its neighbors, when given a unit with strategy X （$X \in \{A, B\}$）. Therefore, the following forms are established:

$$\begin{cases} P_A + P_B = 1 \\ q_{A/X} + q_{B/X} = 1 \\ P_{XY} = q_{Y/X} \times P_X \\ P_{AB} = P_{BA} \end{cases} \quad (5)$$

It is not difficult to find that the state (strategy composition and topological structure) of the whole swarm can be described by two variables: P_A and P_{AA}.

In each round of game, the focal unit play games with all its neighbors. If the number of units holding strategy *A* and *B* are k_A and k_B respectively in the focal unit's neighbors, and $k_A + k_B = k$, then the payoffs of the focal unit, which holds strategy *A* and *B* are established respectively:

$$F_A = \frac{k_A \cdot R + k_B \cdot S}{k} = q_{A/A} \cdot R + q_{B/A} \cdot S \quad (6)$$

$$F_B = \frac{k_A \cdot T + k_B \cdot P}{k} = q_{A/B} \cdot T + q_{B/B} \cdot P \quad (7)$$

Next, we calculate the probability of changing of P_A and P_{AA} in each round of game, which describes the dynamic evolution process of strategies holding by each unit in the swarm. For a unit with *B* strategy, the probability of switching its strategy to *A* is:

$$\text{Prob}(\vartriangle_{P_A} = \frac{1}{N}) = P_B \cdot \sum_{k_A + k_B = k} C_k^{k_A} q_{A/B}^{k_A} q_{B/B}^{k_B} \cdot \frac{1}{1 + e^{\omega(F_B - \bar{F})}} \quad (8)$$

where, P_B is the probability of randomly selecting a unit holding strategy *B* from the swarm; $\sum_{k_A + k_B = k} C_k^{k_A} q_{A/B}^{k_A} q_{B/B}^{k_B}$ is the probability that the structure of unit's neighbor is $k_A + k_B = k$, and the unit holds strategy *B*. In addition, $\frac{1}{1 + e^{\omega(F_B - \bar{F})}}$ represents the strategy update probability.

The updating of strategies will cause the changing of strategy pair in swarm. Because the average degree of swarm is k, the scale of strategy pair in swarm is $kN/2$. We can get the probability that the number of strategy pair AA increases by k_A (i.e., P_{AA} increases by $k_A \Big/ (\frac{kN}{2})$) is:

$$\text{Prob}(\vartriangle_{P_{AA}} = \frac{2k_A}{kN}) = P_B \cdot C_k^{k_A} q_{A/B}^{k_A} q_{B/B}^{k_B} \cdot \frac{1}{1 + e^{\omega(F_B - \bar{F})}} \quad (9)$$

Similarly, for a unit holding strategy *A*, the probability of switching its strategy to *B* is:

Authorized licensed use limited to: HUNAN UNIVERSITY. Downloaded on January 13,2021 at 13:39:37 UTC from IEEE Xplore. Restrictions apply.

$$\mathrm{Prob}(\Delta_{P_A} = -\frac{1}{N}) = P_A \cdot \sum_{k_A+k_B=k} C_k^{k_A} q_{A/A}^{k_A} q_{B/A}^{k_B} \cdot \frac{1}{1+e^{\omega(F_A-\bar{F})}} \quad (10)$$

The probability of strategy pair AA reducing by k_A is:

$$\mathrm{Prob}(\Delta_{P_{AA}} = -\frac{2k_A}{kN}) = P_A \cdot C_k^{k_A} q_{A/A}^{k_A} q_{B/A}^{k_B} \cdot \frac{1}{1+e^{\omega(F_A-\bar{F})}} \quad (11)$$

According to the above strategy updating probability and strategy pair changing probability, as the evolution process advances, the proportion of units holding strategies A and B varies, and the number of strategy pair AA , AB , BA , BB dynamically adjusts. When the evolution advances to a certain time, the proportion of strategy and the number of strategy pairs will not change any more, in such situation, the evolution of the swarm tends to be stable. By analyzing the evolution parameters of the swarm when it is stable and designing a reasonable control mechanism, we can achieve the goal of coordinated control of the swarm.

B. Formation conditions of swarm cooperation

When the swarm size is large enough to satisfy $k \ll N \ll 1/\omega$, the derivative of P_A and P_{AA} with respect to time can be approximately as follows:

$$P_A^{'} = \frac{1}{N} \cdot \mathrm{Prob}(\Delta_{P_A} = \frac{1}{N}) + (\frac{-1}{N}) \cdot \mathrm{Prob}(\Delta_{P_A} = \frac{-1}{N}) \quad (12)$$

$$P_{AA}^{'} = \sum_{k_A=0}^{k} \frac{2k_A}{kN} \mathrm{Prob}(\Delta_{P_{AA}} = \frac{2k_A}{kN}) + \sum_{k_A=0}^{k} \frac{-2k_A}{kN} \mathrm{Prob}(\Delta_{P_{AA}} = \frac{-2k_A}{kN}) \quad (13)$$

The first-order Taylor expansion is carried out for equations (12) and (13) respectively, as follows:

$$P_A^{'} = \frac{P_B - P_A}{2N} + \omega \times \frac{\partial P_A}{\partial \omega}\Big|_{\omega=0} + o(\omega^2) \quad (14)$$

where,

$$\frac{\partial P_A}{\partial \omega}\Big|_{\omega=0} = \frac{1}{4N}[\bar{F}(P_B - P_A) - T \cdot P_{AB} - P \cdot P_{BB} + R \cdot P_{AA} + S \cdot P_{BA}] \quad (15)$$

$$P_{AA}^{'} = \frac{P_{AB} - P_{AA}}{N} + \omega \times \frac{\partial P_{AA}}{\partial \omega}\Big|_{\omega=0} + o(\omega^2) \quad (16)$$

where,

$$\frac{\partial P_{AA}}{\partial \omega}\Big|_{\omega=0} = \frac{1}{2N}[\bar{F}(P_{AB} - P_{AA}) - T \cdot P_{AB} q_{A/B} \frac{k-1}{k} - T \cdot P_{AB}\frac{1}{k} - P \cdot P_{AB} q_{B/B}\frac{k-1}{k} + R \cdot P_{AA} q_{A/A}\frac{k-1}{k} + R \cdot P_{AA}\frac{1}{k} + S \cdot P_{AA} q_{B/A}\frac{k-1}{k}] \quad (17)$$

Let $P_A^{'}$ and $P_{AA}^{'}$ be zero respectively. When ω=0 , the equilibrium solution of evolution stability is obtained as follows:

$$\begin{cases} P_A^* = P_B^* = 1/2 \\ P_{AA}^* = P_{AB}^* = P_{BA}^* = P_{BB}^* = 1/4 \end{cases} \quad (18)$$

The evolution result is that the number of units holding strategy A and B is equal, and given a unit holding strategy X （ $X \in \{A, B\}$ ） , the conditional probability of finding a unit holding strategy Y （ $Y \in \{A, B\}$ ） in its neighbors is the same. Therefore, the units with different strategies are evenly distributed in the spatial topology.

Under the condition of weak selection, the equilibrium should still exist according to the perturbation theory. There is a perturbation term $\Gamma_{XY}(X, Y \in \{A, B\})$ such that:

$$\begin{cases} P_{AA}^* = 1/4 + \omega \times \Gamma_{AA},\ P_{AB}^* = 1/4 + \omega \times \Gamma_{AB} \\ P_{BA}^* = 1/4 + \omega \times \Gamma_{BA},\ P_{BB}^* = 1/4 + \omega \times \Gamma_{BB} \end{cases} \quad (19)$$

Take equation (19) into equation (14), and get:

$$P_A^{'} = \frac{P_B - P_A}{2N} + \omega \times \frac{1}{4N}[\bar{F}(P_B - P_A) - \frac{1}{4}(R + S - T - P)] + o(\omega^2) \quad (20)$$

If the formula (20) is zero and the higher order term $o(\omega^2)$ of ω is ignored when $\omega \ll 1$, we can get $R + S = T + P$, so $P_A^* = P_B^* = 1/2$ is approximately established.

In addition, it should be noted that in the issue of coordinated control of unmanned swarm, it only requires the two adjacent units to choose different strategies when the game reaches equilibrium, but the strategies are symmetrical, that is to say, there is no need to limit the differences of the strategies themselves. In order to characterize the indifference of strategies themselves, that is, the symmetry of payoff matrix, we specify $R = P$ and $S = T$.

Therefore, as long as the preconditions of $R < T \cap S > P$, $R + S = T + P$, $R = P \cap S = T$ are satisfied in the design of game mechanism, the unmanned units in the adjacent position on the network topology can hold different strategies to achieve cooperation and complete the common task efficiently.

IV CASE SIMULATION

In this section, the coordinated control of swarm in fire attack scenario on regular lattice and general topology are simulated according to the swarm evolution dynamics mentioned in section 2 to verify the rationality of the model and the correctness of theoretical analysis, and provide decision support for the realization of unmanned swarm cooperation.

In the task of swarm fire attack, each combat unit has two functions: ammunition support and fire strike, and the unit can switch between the two functions according to the battlefield situation. It is necessary to make the two kinds of units with complementary functions distributed in the neighborhood as close as possible, so as to cooperate closely to complete the fire attack task.

A. Cooperative evolution on regular lattice

Unmanned swarm is represented as $\{\mathrm{Unit}_i | i \in N\}$, and each unit is represented as $\mathrm{Unit}_i = \{S_i, T_i, I_i, F_i\}$, where $S_i \in \{A, B\}$, A is ammunition support, and B represents fire strike; Unit_i implements strategy transformation T_i according to probability based on payoff, which is determined by formula (4); Unit_i conducts game interaction I_i on the regular lattice, which is determined by formula (6)-(11); and Unit_i calculates its fitness F_i according to formula (3). Set the swarm size N=25 , the selection intensity ω=0.01 , the game takes place in von Neumann neighborhood[46]: only four adjacent units of each unit on regular lattice are considered.

The initial strategy distribution is random, each unit adopts strategy A or B randomly, and the units holding strategy A and B account for nearly 50% of the total. The initial strategy distribution of the swarm is shown in Figure 1, where each grid represents a unit, the white grid

Authorized licensed use limited to: HUNAN UNIVERSITY. Downloaded on January 13,2021 at 13:39:37 UTC from IEEE Xplore. Restrictions apply.

represents units holding strategy *A*, and the gray grid represents units holding strategy *B*.

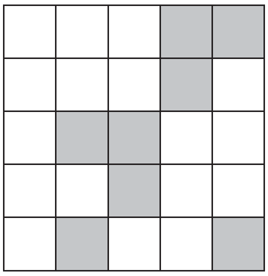

Fig. 1. Initial Strategy Distribution on Regular Lattice

According to the results of theoretical analysis in section 2, on the premise of satisfing $R < \mathrm{T} \cap S > P$, $R + S = T + P$, $R = P \cap S = T$, the payoff matrix is given as follows:

$$\begin{array}{c} \\ A \\ B \end{array}\begin{array}{c} \begin{array}{cc} A & B \end{array} \\ \begin{pmatrix} 1 & 10 \\ 10 & 1 \end{pmatrix} \end{array}$$

The above matrix values reflect the high benefits of choosing the opposite strategy with neighbors. Meanwhile, it can ensure the symmetry and indifference of strategy itself from the payoff perspective.

The evolution process is shown in Figure 2, where Figure 2(a) shows the strategy distribution after the first round of game, and Figure 2(b) shows the final evolution result after several rounds of game iteration and the swarm reaches the evolution stable state.

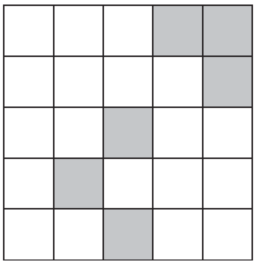

(a) Strategy distribution after the first round of game

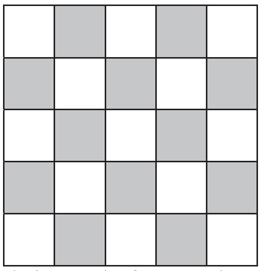

(b) Final evolution result after several rounds of game

Fig. 2. Strategy Evolution Pattern on Regular Graph

The state of the swarm eventually evolves to an optimal distribution, with the units with different strategies evenly distribution in regular lattice, so as to form functional complementarity and cooperation, and efficiently complete the fire attack task.

B. Cooperative evolution on general topology

In this section, we extend the cooperation evolution on regular lattice to general topology. In this case, the connection between units is no longer completely regular. The swarm scale N=25 and the selection intensity ω=0.01 are still considered. The initial strategy distribution is shown in Figure 3.

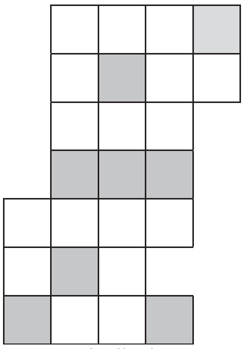

Fig. 3. Initial Strategy Distribution on General Topology

The evolution results are shown in Figure 4.

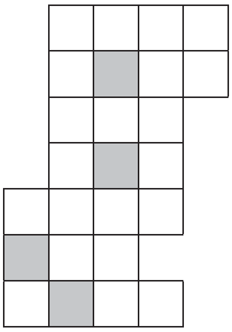

(a) Strategy distribution after the first round of game

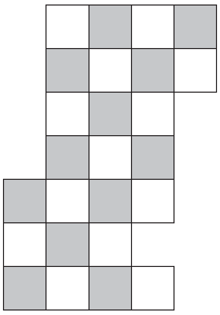

(b) Final evolution result in evolution stable state

Fig. 4. Strategy Evolution Pattern on General Topology

Figure 4(a) shows the strategy distribution after the first round of game, and figure 4(b) shows the final evolution result. The results show that the proposed model can also achieve the goal of coordinated control in general topology.

The above models and methods have domain universality. They are not only suitable for solving the issue of coordinated control in swarm fire attack, but also can be extended to general military, biological and social fields. By designing appropriate game mode and mechanism to meet the payoff constraints, the swarm can eventually achieve independent evolution and cooperation.

V CONCLUTION

Aiming at the problem of coordinated control of unmanned swarm, this research establishes a coordinated control model based on the evolutionary game theory, and theoretically deduces the evolutionary dynamics process and the conditions for the formation of cooperation. Finally, taking the scenario of swarm fire attack as an example, the strategy evolution processes of swarm on regular lattice and general topology are simulated. The simulation results confirm the correctness of theoretical derivation and the rationality of the model. In the actual unmanned swarm coordinated control, by designing appropriate evolutionary game mechanism and payoff parameters, the goal of swarm

Authorized licensed use limited to: HUNAN UNIVERSITY. Downloaded on January 13,2021 at 13:39:37 UTC from IEEE Xplore. Restrictions apply.

cooperation can be achieved. The conclusion of this paper provides a preliminary and meaningful exploration for the transformation of relevant theories into practical applications.

This paper assumes that the swarm structure is static topology and the game type is classic 2×2 game. However, the dynamic change of network topology and multi-dimensional switch between multi strategies are realistic needs in actual combat. How to extend the theoretical methods to the multi strategy game on the dynamic network to realize more accurate and effective coordinated control of swarm is the next research direction.

REFERENCES

[1] Xu P, Dherbomez G, Hery E, et al. System architecture of a driverless electric car in the grand cooperative driving challenge[J]. IEEE Intelligent Transportation Systems Magazine, 2018, 10(1): 47-59.

[2] Liang H, Qiang H, Feng Z. Research on Capability Characteristics Modeling and Cooperative Fire Strike Planning for Unmanned Ground Vehicles[C]//Proceeding of the 2nd International Conference on Artificial Intelligence and Big Data (ICAIBD). IEEE, 2019: 136-141.

[3] Zoto J, Musci M A, Khaliq A, et al. Automatic Path Planning for Unmanned Ground Vehicle Using UAV Imagery[C]//International Conference on Robotics in Alpe-Adria Danube Region. Springer, Cham, 2019: 223-230.

[4] Ma Y, Hu M, Yan X. Multi-objective path planning for unmanned surface vehicle with currents effects[J]. ISA transactions, 2018, 75: 137-156.

[5] Song R, Liu Y, Bucknall R. Smoothed A* algorithm for practical unmanned surface vehicle path planning[J]. Applied Ocean Research, 2019, 83: 9-20.

[6] Zhan Z, Zhou Q, Li B. Research on the key technologies of unmanned cluster to sea combat[C]//Global Intelligence Industry Conference (GIIC 2018). International Society for Optics and Photonics, 2018, 10835: 108351K.

[7] Giles K, Giammarco K. A mission-based architecture for swarm unmanned systems[J]. Systems Engineering, 2019, 22(3): 271-281.

[8] Khawaja W, Guvenc I, Matolak D W, et al. A Survey of Air-to-Ground Propagation Channel Modeling for Unmanned Aerial Vehicles[J]. IEEE Communications Surveys and Tutorials, 2019, 21(3): 2361-2391.

[9] Shakeri R, Algaradi M A, Badawy A, et al. Design Challenges of Multi-UAV Systems in Cyber-Physical Applications: A Comprehensive Survey and Future Directions[J]. IEEE Communications Surveys and Tutorials, 2019, 21(4): 3340-3385.

[10] Dolicanin E, Fetahovic I, Tuba E, et al. Unmanned combat aerial vehicle path planning by brain storm optimization algorithm[J]. Studies in Informatics and Control, 2018, 27(1): 15-24.

[11] Fan J, Li D, Li R, et al. Analysis on MAV/UAV cooperative combat based on complex network[J]. Defence Technology, 2019.

[12] Smith J M, Price G R. The logic of animal conflict[J]. Nature,1973, 246(5427):15-18.

[13] Nowak M A, Sigmund K. Evolution of indirect reciprocity[J]. Nature, 2005, 437(6685): 1291-1298.

[14] Nowak M A. Evolving cooperation[J]. Journal of theoretical biology, 2012, 299(0): 1-8.

[15] Takesue H, Ozawa A, Morikawa S. Evolution of favoritism and group fairness in a co-evolving three-person ultimatum game[J]. Europhysics Letters, 2017, 118(4): 48002.

[16] Nowak M A. Five rules for the evolution of cooperation[J]. Science, 2006, 314(5805): 1560-1563.

[17] Gore J, Youk H, Van Oudenaarden A, et al. Snowdrift game dynamics and facultative cheating in yeast[J]. Nature, 2009, 459(7244): 253-256.

[18] Su Q, McAvoy A, Wang L, et al. Evolutionary dynamics with stochastic game transitions[J]. arXiv preprint arXiv:1905.10269, 2019.

[19] Hindersin L, Wu B, Traulsen A, et al. Computation and simulation of evolutionary Game Dynamics in Finite populations[J]. Scientific reports, 2019, 9(1): 6946.

[20] Nowak M A, Tarnita C E, Antal T, et al. Evolutionary dynamics in structured populations[J]. Philosophical Transactions of the Royal Society B, 2010, 365(1537): 19-30.

[21] Pena J, Wu B, Arranz J, et al. Evolutionary Games of Multiplayer Cooperation on Graphs[J]. PLOS Computational Biology, 2016, 12(8).

[22] Du J, Wu B, Wang L, et al. Aspiration dynamics in structured population acts as if in a well-mixed one[J]. Scientific Reports, 2015, 5(1): 8014-8014.

[23] Du J M, Wu B, Altrock P M, et al. Aspiration dynamics of multi-player games in finite populations[J]. Journal of the Royal Society Interface, 2014, 11(94): 20140077.

[24] Du J M, Wu B, Wang L. Evolutionary game dynamics of multi-agent cooperation driven by self-learning [C]//2013 9th Asian Control Conference (ASCC). IEEE, 2013: 1-6.

[25] Sui X, Cong R, Li K, et al. Evolutionary dynamics of N-person snowdrift game[J]. Physics Letters A, 2015, 379(45-46): 2922-2934.

[26] Jiang L, Perc M, Szolnoki A, et al. If Cooperation Is Likely Punish Mildly: Insights from Economic Experiments Based on the Snowdrift Game[J]. PLOS ONE, 2013, 8(5).

[27] Ohtsuki H, Hauert C, Lieberman E, et al. A simple rule for the evolution of cooperation on graphs and social networks[J]. Nature,2006,441(7092): 502-505

[28] Fu F, Nowak M A, Hauert C. Invasion and expansion of cooperators in lattice populations: Prisoner's dilemma vs. snowdrift games[J]. Journal of Theoretical Biology, 2010, 266(3):358-366.

[29] Maciejewski W, Fu F, Hauert C. Evolutionary game dynamics in populations with heterogenous structures[J]. PLoS computational biology, 2014, 10(4).

[30] Fotouhi B, Momeni N, Allen B, et al. Evolution of cooperation on large networks with community structure[J]. Journal of the Royal Society Interface, 2019, 16(152): 20180677-20180677.

[31] Josef T, Andreas P, Krishnendu C, et al. Population structure determines the tradeoff between fixation probability and fixation time.[J]. Communications Biology, 2019, 2.

[32] Allen B, Lippner G, Nowak M A. Evolutionary games on isothermal graphs[J]. Nature communications, 2019, 10(1): 1-9.

[33] Marta D S, Flávio L P, Francisco C S, et al. Dynamics of N-person snowdrift games in structured populations[J]. Journal of Theoretical Biology, 2012, 315:81-86.

[34] Li P P, Ke J, Lin Z, et al. Cooperative behavior in evolutionary snowdrift games with the unconditional imitation rule on regular lattices [J]. Physical Review E, 2012, 85(2): 021111.

[35] György S, Gábor F. Evolutionary games on graphs[J]. 2007, 446(4-6):97-216.

[36] Wang X J, Xia K. Extended average abundance function of multi-player snowdrift evolutionary game under aspiration driven rule[J]. Systems Engineering Theory&Practice, 2019, 39(5): 1128-1137.

[37] Wang L, Tian Y, Du J M. Opinion dynamics in social networks[J]. SCIENTIA SINICA Information, 2018, 48(01): 8-28.

[38] Smith E A. Communication and collective action: language and the evolution of human cooperation[J]. Evolution & Human Behavior, 2010, 31(4): 231-245.

[39] Yu M G, Zhang D G, Kang K, et al. Cooperative Evolution Mechanism of Unmanned Cluster based on Multi-player Public Goods Game[J/OL]. Journal of Systems Engineering and Electronics, 1-10[2020-05-21]. http://kns.cnki.net/kcms/detail/11.2422.TN.20200518.1133.028.html.

[40] Xu K, Li K, Cong R, et al. Cooperation guided by the coexistence of imitation dynamics and aspiration dynamics in structured populations[J]. Europhysics Letters, 2017, 117(4): 48002.

[41] Wang X J, Gu C L, Lv S J, et al. Evolutionary game dynamics of combining the Moran and imitation processes[J]. Chinese Physics B, 2019, 28(2): 1-13.

[42] Chen Y S, Yang H X, Guo W Z. Aspiration-induced dormancy promotes cooperation in the spatial Prisoner's Dilemma games[J]. Physica A: Statistical Mechanics and its Applications, 2017, 469: 625-630.

[43] Taylor P D, Jonkrt L B. Evolutionary stable strategies and game dynamics[J]. Mathematical Biosciences, 1978, 40(1-2): 145-156.

[44] Nowak M A, Sigmund K. Evolitionary dynamics of biologival games[J]. Science, 2004, 303(5659): 793-799.

[45] Wu B, Altrock P M, Wang L, et al.. Universality of weak selection[J]. Physical Review E, 2010, 82(4): 046106.

[46] Su Q, Wang L, Stanley H E. Understanding spatial public goods games on three-layer networks. New Journal of Physics, 2018. 20(10): 103030.

Authorized licensed use limited to: HUNAN UNIVERSITY. Downloaded on January 13,2021 at 13:39:37 UTC from IEEE Xplore. Restrictions apply.
