# Supplementary material for: A game-based approach for designing a collaborative evolution mechanism for unmanned swarms on community networks
Source: Sci Rep. 2022 Nov 7;12:18892. doi: 10.1038/s41598-022-22365-z (PMC9640601; doi:10.1038/s41598-022-22365-z)
Supplement: Supplementary file 1 — Supplementary Information. [file 41598_2022_22365_MOESM1_ESM.zip › Supporting material/3.í╢Evolutionary mechanism of unmanned swarms cooperation based on evolutionary game of multiple public goodsú¿╗∙╙┌╢α╘¬╣1⁄2╣▓╞╖╤▌╗»▓⌐▐─╡─╬▐╚╦╝»╚║║╧╫≈╤▌╗»╗·╓╞ú⌐í╖.pdf]

文章编号:1001-506X(2020)12-2787-08 网址:www. sys-ele. com

基于多元公共品演化博弈的无人集群合作演化机制

禹明刚[1,2],张东戈[1],康 凯[1],朱卫星[1]
(1. 陆军工程大学指挥控制工程学院,江苏 南京 210007;
2. 陆军工程大学通信工程学院,江苏 南京 210007)

摘 要:针对控制规则失效下的无人集群自主协同问题,提出了一种基于多元公共品演化博弈的无人集群合作演化机制。首先分析无人集群自主协同需求,在愿景驱动规则下,构建多元公共品演化博弈模型。在此基础上,理论推导出模型的平均丰度函数,并仿真计算成本、收益系数、愿景水平三类参数分别对平均丰度的影响。最后,结合案例研究参数变化对集群合作行为演化的作用机理,并给出合理化建议,为相关理论向实际应用转化提供有意义的探索。

关键词:无人集群;合作演化;多元公共品演化博弈;愿景驱动
中图分类号:TP 391 **文献标志码**:A **DOI**:10.3969/j.issn.1001-506X.2020.12.15

Cooperative evolution mechanism of unmanned cluster based on multi-player public goods evolutionary game

YU Minggang[1,2], ZHANG Dongge[1], KANG Kai[1], ZHU Weixing[1]
(*1. Institute of Command and Control Engineering, Army Engineering University, Nanjing 210007, China;*
2. Institute of Communication Engineering, Army Engineering University, Nanjing 210007, China)

Abstract: To solve the problem of the autonomous collaboration of unmanned cluster under control rule failure, a cooperative evolution mechanism of unmanned cluster based on multi-player public goods evolutionary game is proposed. It starts with the requirement analysis of cooperative evolution in the unmanned cluster. And, the evolutionary game model of the multi-player public goods based on the aspiration driven rule is established. Then the average abundance function is constructed by theoretical derivation. Furthermore, the influence of cost, multiplication factor and aspiration level on the average abundance is simulated. Finally, the mechanism of the parameter change on the evolutionary cluster cooperation behavior is revealed via case study, and the rationalization proposals are suggested to provide a meaningful exploration for the transformation from theory to application.

Keywords: unmanned cluster; cooperative evolution; multi-player public goods evolutionary game; aspiration driven

0 引 言

美军认为,未来战争将是智能化战争,无人系统将是未来战争的主力军,智能化协同的无人系统集群作战是未来战争的主要形态[1]。

无人集群作战中,在通信良好的前提下,指控中心可以对集群实施集中式协同控制。然而,现代战场电磁环境复杂,通信不畅甚至通信失效的情况是常见的现实风险[2]。

收稿日期:2020—03—20;**修回日期**:2020—04—17;**网络优先出版日期**:2020—05—18。
网络优先出版地址:http://kns.cnki.net/kcms/detail/11.2422.TN.20200518.1133.028.html
基金项目:国家自然科学基金(71901217);国家社会科学基金(19BXW117)资助课题
引用格式:禹明刚,张东戈,康凯,等. 基于多元公共品演化博弈的无人集群合作演化机制[J]. 系统工程与电子技术,2020,42(12):2787-2794.
Reference format: YU M G, ZHANG D G, KANG K, et al. Cooperative evolution mechanism of unmanned cluster based on multi-player public goods evolutionary game[J]. Systems Engineering and Electronics, 2020, 42(12):2787-2794.

(C)1994-2020 China Academic Journal Electronic Publishing House. All rights reserved. http://www.cnki.net

此时,无人集群必须根据自我获取的态势,临时做出有效响应。做到自我管理、自主协同,以继续完成既定军事行动。

无人集群的自主协同,需要临时对通信、情报、火力等各类作战资源进行重新配置,以保障战场生存及完成任务的能力。然而资源重新配置中个体"偏私性"与集群整体需求,往往存在难以调和的矛盾。例如,在集群火力打击任务中,具备独立决策能力的理性作战单元为确保自身实现最大战场价值,会选择尽可能少地向集群贡献"保障型"弹药,从而确保自身具有尽可能多的"战斗型"弹药保有量;从集群层面看,每个作战单元向集群贡献的"保障型"弹药越多,则集群整体生存率越高,作战效能越大。这两者之间的选择将引发"公地悲剧"[3]。如何处理好公共资源,避免公地悲剧,是无人集群技术研究及现实应用中一项至关重要且亟待解决的难题。

演化博弈[4-11]将经济学的"均衡观"与生物学的"适应性"理念进行了很好的结合,刻画了在理性不完全、信息不对称、环境及预期存在偏差的条件下,群体通过学习、模仿、试错而不断适应外部环境的过程。Smith 等首次将 Von Neumann 开创的经典博弈论观点"大脑在利益冲突时做出理性决定"扩展为"根据自然选择而得出决定"[5],开创了演化博弈论的新纪元。近年来,众多学者利用演化博弈理论研究合作涌现促进机制,以哈佛大学 Nowak 教授为代表,对"综合进化论"进行扩展,总结了促进合作涌现的五大机制[6-8]。演化博弈论为研究合作演化提供了重要数学框架。

其中,公共物品演化博弈[3,12]以公共物品投资为背景,反映了投资者(合作者)与搭便车者(背叛者)随着时间推移,基于投资成本、收益系数、选择强度等众多参数进行策略博弈,使得群体中合作者和背叛者的占比发生动态变化,最终会趋于演化稳定状态。公共物品演化博弈为揭示合作演化机理、解决公地悲剧提供了基础理论支撑。对该博弈过程的研究重点是,计算经多轮博弈后合作者在集群中占比的期望值,即平均丰度,进而分析其与相关参数的函数关系,揭示不同参数对于演化行为的影响,最终实现管控的目的。

王先甲等[13]通过分析马尔可夫链的平稳分布,将多人演化博弈模型应用于雪堆演化博弈,得到多人雪堆演化博弈模型的扩展平均丰度函数,计算分析了相关参数对平均丰度的影响。杜金铭等[14]在 TARNITA[15]的研究基础上,通过严格数学推导得出两方演化博弈中策略占优条件不等式,仿真发现,弱选择强度下平均丰度值与愿景水平无关。上述对平均丰度值的研究有很高的理论价值和工程价值。

然而,在解决无人集群合作演化问题时,上述成果仍存在两点不足:一是尚未聚焦于公共物品博弈,虽然雪堆博弈与公共物品博弈在模型上有相似之处[16],但在博弈机制上存在本质区别;二是无人集群的合作演化是多个作战单元博弈的多重互动,演化结果不仅与自身策略选择相关,更取决于同一组中其他单元的策略,表现为多方博弈[17-18]的特征。目前,学术界已经掌握了公共物品博弈在多方参与下的支付矩阵[19],并仿真分析了不同选择强度[20-22]、不同上下门限取值[16]对协作水平的影响,但尚未发现关于多方公共物品博弈平均丰度函数的研究成果。本文的一项重要工作即是基于现有支付矩阵获取公共物品博弈的平均丰度解析表达式。

此外,广义上的演化博弈模型可简化为"马尔可夫链+策略"更新机制,平均丰度函数的确定与策略更新机制密切相关。策略更新有两种机制:模仿动态和愿景驱动动态[23]。对于前者,个体通过与其他个体的支付比较,决定是否模仿其他个体的策略[24];对于后者,若个体期望水平未得到满足,则个体调整其策略[15,19,25]。比较而言,愿景驱动动态侧重于将演化博弈收益与愿景水平的比较,进而做出新一轮决策,强化了个体主观愿望在博弈中的作用,一般较少考虑策略环境影响[26-27]。在复杂战场环境下,无人集群信息获取不完全、不对称、对策略环境预期存在偏差,这就要求集群系统能实现自我管理与自我协同。现有成果表明,无论在囚徒困境博弈还是公共物品博弈中,愿景驱动的动态机制相比于传统模仿动态,更能提高平均丰度值,进而促进合作[20,28-30]。

针对无人集群合作演化机制,本文基于多元公共品演化博弈模型,采用愿景驱动规则对无人集群合作演化建模,通过分析无吸收态的马尔可夫链的平稳分布,推导出模型的平均丰度函数。在此基础上,通过理论分析与数值计算相结合研究相关参数对平均丰度的影响。最后,结合案例具体研究了参数变化对集群合作行为演化的作用机理,据此给出有效避免"公地悲剧",促进无人集群合作行为产生的建议。

1 模型假设

无人集群自主协同是一个多方参与、多轮迭代的公共物品自主分配博弈过程。因此,用多元公共品演化博弈,对集群自主协同过程进行建模。首先,无人集群自主协同与多元公共品演化博弈相关概念的映射关系如表 1 所示。

表 1 概念映射

Table 1 Concept mapping

无人集群自主协同	多元公共品演化博弈
无人集群	混合均匀种群
自主协同所需公共资源(弹药、通信等)	公共品
参与自主协同的多个无人平台	多元
单个无人平台	个体
作为研究对象的单个无人平台	焦点个体
平台愿意向集群贡献资源的合作行为	策略 A
平台不愿向集群贡献资源,而选择"搭便车"的非合作行为	策略 B
不同策略下,集群回馈给平台的公共资源	收益
无人平台间基于收益的策略转换	博弈
多轮博弈中,采用不同策略的平台在集群中占比动态变化	演化
多轮博弈后,占比稳定,博弈终止	演化稳定

(C)1994-2020 China Academic Journal Electronic Publishing House. All rights reserved. http://www.cnki.net

1.1 多元公共品演化博弈框架

设多元演化博弈发生在一个数量为 N 的混合均匀种群中，每个个体在策略集$\{A,B\}$中进行策略更新。某时刻，若种群中选择策略 A 的个体(类型 A)数为 i，则选择策略 B 的个体(类型 B)数为 $N-i$，随着多轮演化过程的推进，类型 A 与类型 B 在种群中的占比将动态更新，并最终趋于稳定。

本文将多元演化博弈过程抽象为 3 个步骤。

步骤 1 在数量为 N 的种群中任取一个焦点个体 X (类型 A 或类型 B)，同时在余下的 $N-1$ 个个体中选取 $d-1$ 个个体，构成规模为 $d(d<N)$ 的一个组。若组中类型 A 的个体规模为 $k(0\leqslant k\leqslant d-1)$，则类型 B 的个体规模为 $d-k-1$。

步骤 2 焦点个体 X 在组内与其余 $d-1$ 个个体发生博弈，即各方分别在$\{A,B\}$中选择博弈策略。若 X 为类型 A 则其收益为 a_k，若是类型 B 则为 b_k。

步骤 3 每轮博弈结束后，焦点个体 X 评估其不同策略选择下的收益，然后依据模仿或愿景驱动机制更新策略。

上述过程重复推进，直到某类型在整个种群中的占比趋于稳定。k 的不同取值决定了焦点个体 X 的收益 a_k 与 b_k，多元演化博弈中单次博弈中 X 的收益矩阵如表 2 所示。

表 2 多元演化博弈中单次博弈收益矩阵

Table 2 Single game income matrix of multi-player evolutionary game

类型	个数				
	0	$d-1$	…	k	…
A	a_{d-1}	…	a_k	…	a_0
B	b_{d-1}	…	b_k	…	b_0

将多元演化博弈模型运用到公共物品博弈问题中，策略 A 为合作策略，即参与投资；策略 B 反之。当 X 选择策略 A，则组中个体总贡献量为 $kc+c$，乘以收益系数 r 后总产出为 $r(kc+c)$，每个个体收益为 $r(kc+c)/d$，由于 X 付出成本为 c，因而其净收益为 $r(kc+c)/d-c$；当 X 选择策略 B，则组中个体总贡献量为 kc，总产出为 rkc，每个个体收益为 rkc/d，由于 X 无成本付出，因而其净收益为 rkc/d。因此，a_k 与 b_k 的具体形式为

$$a_k=r(kc+c)/d-c \tag{1}$$

$$b_k=rkc/d \tag{2}$$

式中，成本 c 为个体向集群贡献的资源(公共品)；收益系数 r 为衡量个体向集群贡献资源后，集群反馈给个体的利益大小的系数。

1.2 博弈期望收益

由上述多元演化博弈模型可知，组中 d 个个体的类型是随机的，因此焦点个体 X 在组内与其余 $d-1$ 个个体发生博弈时，其遇到 k 个类型 A 个体和 $d-k-1$ 个类型 B 个体的概率满足超几何分布的数学约束[31]。以类型 A 的焦点个体 X 为例，其遇到 k 个类型 A 个体和 $d-k-1$ 个类型 B 个体的概率为

$$P_A(N,i;d,k)=\mathrm{C}_{i-1}^{k}\mathrm{C}_{N-i}^{d-k-1}/\mathrm{C}_{N-1}^{d-1} \tag{3}$$

式中，i 为种群 N 中类型 A 的数量；C_{i-1}^{k} 和 C_{N-i}^{d-k-1} 分别为选取类型 A、类型 B 的个体组合数；C_{N-1}^{d-1} 为参加选择的个体组合数。概率 $P_A(N,i;d,k)$ 对应于收益 a_k，一个类型 A 焦点个体 X 在一轮博弈中面临 d 种可能的相遇获得 d 种可能的收益。因此，类型 A 焦点个体 X 在某轮博弈中的期望收益为

$$\pi_A(i)=\sum_{k=0}^{d-1}\frac{\mathrm{C}_{i-1}^{k}\mathrm{C}_{N-i}^{d-k-1}}{\mathrm{C}_{N-1}^{d-1}}a_k \tag{4}$$

同理，$P_B(N,i;d,k)=\mathrm{C}_{i}^{k}\mathrm{C}_{N-i-1}^{d-k-1}/\mathrm{C}_{N-1}^{d-1}$，一个类型 B 焦点个体 X 在某轮博弈中的期望收益为

$$\pi_B(i)=\sum_{k=0}^{d-1}\frac{\mathrm{C}_{i}^{k}\mathrm{C}_{N-i-1}^{d-k-1}}{\mathrm{C}_{N-1}^{d-1}}b_k \tag{5}$$

1.3 愿景驱动规则

目前在演化博弈中主要存在无条件模仿[4,32]、复制动态[33]、费米规则[34]、莫兰过程[35-36]等几类典型策略更新规则，其中愿景驱动规则属于费米规则的特例，侧重于将演化博弈收益与愿景水平比较而进行新的决策，该规则强化了个体主观愿望在博弈中的作用，主观愿望是焦点个体的本质特征，反映了焦点个体对于博弈收益的期望(或“贪婪”程度)。在愿景驱动规则与复杂战场环境中，无人集群信息获取不完全、策略预期存在偏差的情况下，实现自我管理与自我协同的需求不谋而合。在该规则驱动下，焦点个体从类型 A 更新为类型 B 的概率为

$$P_{A\to B}=\frac{1}{1+\mathrm{e}^{\omega(\pi_A(i)-\alpha)}} \tag{6}$$

式中，α 为焦点个体愿景水平；ω 为选择强度，其可放大或缩小 $\pi_A-\alpha$ 对策略更新概率的影响。令 $\Delta=\pi_A-\alpha$，若 $\Delta=0$，则 $P_{A\to B}=1/2$，个体对策略 A、B 的偏好相同；若 $\Delta>0$ (即个体收益 π_A 高于愿景水平 α 时)，则 $P_{A\to B}<1/2$，此时个体更偏向于选择策略 A；若 $\Delta<0$(即个体收益 π_A 低于愿景水平 α 时)，则 $P_{A\to B}>1/2$，此时个体更偏向于选择策略 B。

同理，焦点个体从类型 B 更新为类型 A 的概率为

$$P_{B\to A}=\frac{1}{1+\mathrm{e}^{\omega(\pi_B(i)-\alpha)}} \tag{7}$$

基于愿景驱动的多元演化博弈模型中，类型 A 个体数量的动态变化有 3 种趋势：①$i\to i-1$；②$i\to i+1$；③$i\to i$。

根据式(6)和式(7)可得对应的转移概率，此概率刻画了种群中类型 A 个体的演化过程，该过程通常用无吸收态的马尔可夫链状态方程来描述：

$$P(i\to i-1)=T_i^-=\frac{i}{N}\cdot\frac{1}{1+\mathrm{e}^{\omega(\pi_A(i)-\alpha)}} \tag{8}$$

$$P(i\to i+1)=T_i^+=\frac{N-i}{N}\cdot\frac{1}{1+\mathrm{e}^{\omega(\pi_B(i)-\alpha)}} \tag{9}$$

$$P(i\to i)=1-T_i^--T_i^+ \tag{10}$$

(C)1994-2020 China Academic Journal Electronic Publishing House. All rights reserved. http://www.cnki.net

由于无吸收态的马尔可夫链在数学上存在一个平稳分布，因此基于上述状态方程可推导出多元演化博弈的平均丰度函数。

2 平均丰度函数

在目前的平均丰度研究中，大多基于采用数字模拟方式，得到的定义式并未给出严格的数学表达式。本节通过分析无吸收态马尔可夫链的平稳分布，推导出多元公共品演化博弈模型的平均丰度函数。

2.1 平均丰度

定义 1　平均丰度。集群中类型 A 个体比例 j/N 为随机变量，设 $v(j)$ 为该随机变量的概率分布，则易得集群中类型 A 个体比例的期望值，定义该期望值为集群中类型 A 的平均丰度。

由以上定义可知，平均丰度 $<X_A(j)>$ 定义式可表示为

$$<X_A(j)>=\sum_{j=0}^{N}\frac{j}{N}v(j) \tag{11}$$

平均丰度计算的关键是确定随机变量的概率分布 $v(j)$。对于无吸收态的马尔可夫链，$v(j)$ 即为马尔可夫链的平稳分布 $\varphi_j(j\in[0,N])$，而平稳分布 φ_j 满足细节平衡条件[37-38]：$\varphi_j T_j^+=\varphi_{j+1}T_{j+1}^-$。

2.2 函数推导

式(11)为定义式，无法直接运用到实际的计算中，下节将以细节平衡条件为切入点，理论推导出平均丰度展开式，以直观揭示平均丰度与各参数间的定量关系，为后续的特性分析即实际应用提供理论计算模型。

由细节平衡条件可知

$$\varphi_1=\frac{T_0^+}{T_1^-}\varphi_0 \tag{12}$$

$$\varphi_2=\frac{T_1^+}{T_2^-}\varphi_1=\frac{T_0^+T_1^+}{T_1^-T_2^-}\varphi_0 \tag{13}$$

$$\varphi_3=\frac{T_2^+}{T_3^-}\varphi_2=\frac{T_0^+T_1^+T_2^+}{T_1^-T_2^-T_3^-}\varphi_0 \tag{14}$$

对式(12)～式(14)进行归纳分析可得

$$\varphi_j=\frac{\prod_{i=0}^{j-1}T_i^+}{\prod_{i=1}^{j}T_i^-}\varphi_0=\prod_{i=0}^{j-1}h(i)\varphi_0,\ j\geqslant 1 \tag{15}$$

式中，$h(i)=T_i^+/T_{i+1}^-$，为类型占优函数。若 $h(i)>1$，即类型 A 增加的概率大于减小的概率，表示种群中类型 A 占优，反之类型 B 占优。

由于平稳分布 φ_j 满足 $\sum_{j=0}^{N}\varphi_j=1$，因此：

$$\sum_{j=0}^{N}\varphi_j=\varphi_0+\sum_{j=1}^{N}\prod_{i=0}^{j-1}h(i)\varphi_0=1 \tag{16}$$

由此解出 $\varphi_0=\dfrac{1}{1+\sum_{j=1}^{N}\prod_{i=0}^{j-1}h(i)}$，代入式(15)得

$$\varphi_j=\frac{\prod_{i=0}^{j-1}h(i)}{1+\sum_{j=1}^{N}\prod_{i=0}^{j-1}h(i)},\ j\geqslant 1 \tag{17}$$

将式(17)代入式(11)可得平均丰度展开式为

$$<X_A(j)>=\frac{1}{N}\sum_{j=1}^{N}j\frac{\prod_{i=0}^{j-1}h(i)}{1+\sum_{j=1}^{N}\prod_{i=0}^{j-1}h(i)}=\frac{1}{N}\frac{\sum_{j=1}^{N}j\prod_{i=0}^{j-1}h(i)}{1+\sum_{j=1}^{N}\prod_{i=0}^{j-1}h(i)} \tag{18}$$

$$h(i)=\frac{T_i^+}{T_{i+1}^-}=\frac{(N-i)(1+\mathrm{e}^{\omega(\pi_A(i+1)-\alpha)})}{(i+1)(1+\mathrm{e}^{\omega(\pi_B(i)-\alpha)})} \tag{19}$$

实际上，式(18)为愿景驱动规则下多元演化博弈的平均丰度通用表达式，其具体应用取决于 a_k 和 b_k，因此组合式(1)～式(4)、式(4)、式(5)、式(18)和式(19)就构建出了多元公共品演化博弈的平均丰度函数。

3 演化博弈分析

基于多元公共品博弈的平均丰度展开式，在已知种群数量 N、群组规模 d 情况下，分析成本 c、收益系数 r、愿景水平 α 对平均丰度的影响。选取基本参数 $N=100,d=15,c=1,r=1.3,\alpha=1$，当计算某个参数的影响时，其他参数保持不变。另外，为了反映不同选择强度下，相应参数对平均丰度影响程度的不同，特选取 ω 分别为 0、5、10、15、20。将上述参数代入式(18)进行计算并绘制出仿真曲线。

3.1 成本对平均丰度的影响

由式(1)和式(2)可知，增加 c 将增大 a_k、b_k，进而增大 $\pi_A(i)$、$\pi_B(i)$，导致 T_i^+、T_i^- 均减小。由于 $h(i)=T_i^+/T_{i+1}^-$，因此在其他参数保持不变，成本 c 增加的情况下，$h(i)$ 的变化情况难以确定，丰度变化情况无法确定。

选取成本区间 $c\in[0.6,1.5]$，绘制策略 A 的平均丰度 X_A 变化曲线如图 1 所示。

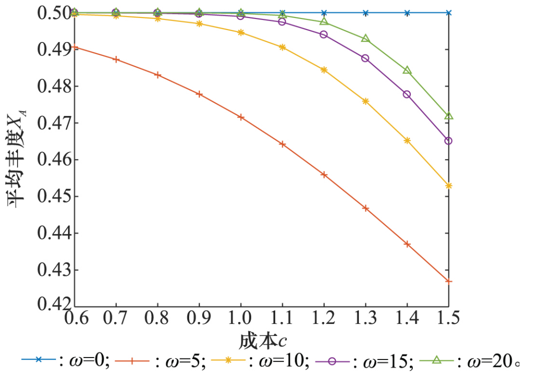

图 1　平均丰度与成本间的关系曲线

Fig. 1　Relationship curve between average abundance and cost

由图 1 可知，随着 c 的增加，X_A 呈单减趋势；$\omega=0$ 时，$X_A=0.5$(集群中的合作者与背叛者比例均衡)；$\omega\neq 0$ 时，同

(C)1994-2020 China Academic Journal Electronic Publishing House. All rights reserved.　http://www.cnki.net

样的成本下，ω 越大，X_A 越大；此外，随着 ω 减小，c 对 X_A 的影响增加：$\Delta<X_A(\omega=20)>\approx0.028$，$\Delta<X_A(\omega=5)>\approx0.063$。

结论 1　c 的增加会减小平均丰度，特别是当 ω 较小时较为显著。

3.2　收益系数对平均丰度的影响

增加收益系数 r，$h(i)$ 的变化情况难以确定，丰度变化情况无法确定。选取收益系数区间 $r\in[0.9,1.8]$，绘制策略 A 的平均丰度 X_A 变化曲线如图 2 所示。

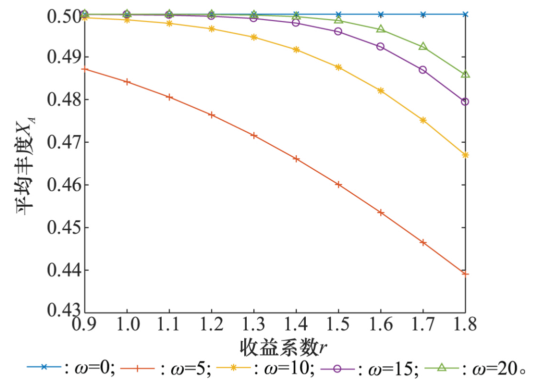

图 2　平均丰度与收益系数间的关系曲线

Fig. 2　Relationship curve between average abundance and benefit coefficient

由图 2 可知，随收益系数 r 的增加，X_A 呈单减趋势，这表明收益系数的增加，集群中“搭便车”现象大量出现，合作现象减弱，导致平均丰度下降；并且，随 $\omega(\omega\neq0)$ 减小，r 对 X_A 的影响增加：$\Delta<X_A(\omega=20)>\approx0.014$，$\Delta<X_A(\omega=5)>\approx0.047$。

结论 2　r 的增加会减小平均丰度，特别是当 ω 减小时较为显著。

3.3　愿景水平对平均丰度的影响

同样，在愿景水平 α 增加的情况下，$h(i)$ 和丰度变化情况无法确定。选取 $\alpha\in[0.9,1.4]$，绘制策略 A 的平均丰度 X_A 变化曲线如图 3 所示。

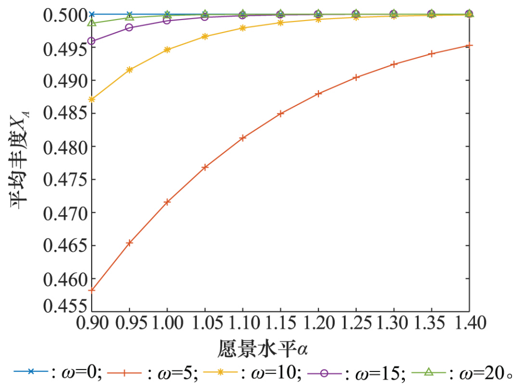

图 3　平均丰度与愿景水平间的关系曲线

Fig. 3　Relationship curve between average abundance and aspiration level

由图 3 可知，随着 α 的增加，X_A 呈增加趋势，这表明 α 的提高，使得非合作收益更难以达到其期望水平，策略更新概率 $P_{B\to A}$ 增加，更多的非合作者转变为合作者；并且，随 ω $(\omega\neq0)$ 减小，α 对 X_A 的影响增加：$\Delta<X_A(\omega=20)>\approx0.002$，$\Delta<X_A(\omega=5)>\approx0.037$。

结论 3　α 的增加会使平均丰度增加，特别是在 ω 较小时较为显著。

以上结论表明，参数 c、r、α 对平均丰度的曲线走势产生影响。其中 c、r 对平均丰度的影响表现为，当 c、r 增加时，平均丰度呈单调递减；而 α 对平均丰度的影响表现为，随 α 增加，平均丰度呈单调递增。上述结论为实际中对集群的合作演化调控提供了理论依据。

4　案例分析

通过对相关参数的调控为实战中无人集群的合作演化与自主控制提供可行建议。

受限于弹药装(挂)载能力，无人集群共同执行火力打击任务时，理性的单个平台将严格控制投资成本 c，以“搭便车”心态控制弹药发射(投放)量；在整个集群层面，则希望各平台提供尽可能多的弹药，以保证整体打击效能。解决该矛盾的关键是，集群如何在人工控制手段失效的前提下，通过自我调控，提升集群中合作者占比，保证火力打击任务的持续有效遂行。

与第 3 节一致，选取基本参数 $N=100$，$d=15$，$c=1$，$r=1.3$，$\alpha=1$，据此绘制出基本曲线如图 4 所示。由于 $X_A<0.5$，本案例为非占优案例，即较多无人作战平台选择非合作策略，尝试通过调控相关参数以促进合作现象的发生。

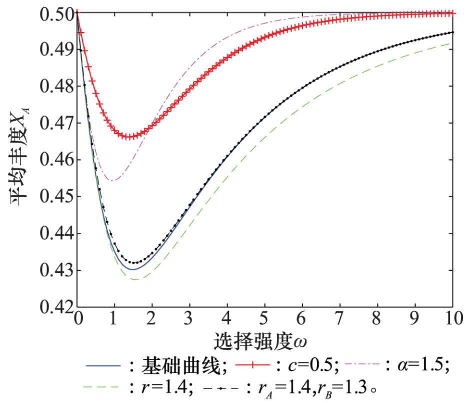

图 4　不同参数下平均丰度与选择强度间的关系曲线

Fig. 4　Relationship curve between average abundance and selection intensity under different parameters

由图 4 可知，减小成本 c 或增加愿景水平 α 都能提高合作单元的占比，而增加收益系数 r，平均丰度曲线将向下偏离基础曲线，这是由于同时增加合作单元和非合作单元的收益，将会使得“搭便车”现象更加严重。为此，本文尝试将合作单元的收益系数和非合作单元的收益系数分开，仅增加合作单元的收益系数 r_A，此时平均丰度曲线向上偏离基础曲线。

(C)1994-2020 China Academic Journal Electronic Publishing House. All rights reserved.　http://www.cnki.net

进一步，本文计算了不同 r_A 下的平均丰度曲线如图 5 所示。

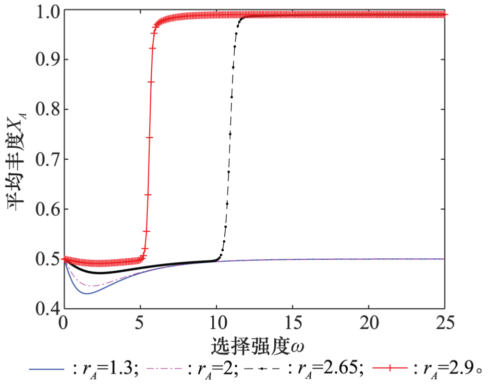

图 5　不同 r_A 下平均丰度与选择强度间的关系曲线

Fig. 5　Relationship curve between average abundance and selection intensity under different r_A

当 $r_A=2$ 时，平均丰度近似等于 0.5，这表明集群中合作单元与非合作单元的占比基本平衡。随着 r_A 的进一步增加，$r_A=2.65$ 时平均丰度在 $\omega\approx10$ 处将大于 0.5；而当 $r_A=2.9$ 时平均丰度在 $\omega\approx5$ 处大于 0.5。这表明：

（1）对 r_A 的调控可实现占优策略的转变，使得平均丰度大于 0.5；

（2）越低的选择强度对 r_A 的要求越苛刻，越高的选择强度对 r_A 的要求越宽松。

为了考察各参数调控结果的灵敏度，仿真了 c、α、r_A 的单位变化量与平均丰度的关系曲线。这里选取 ω 分别为 0、5、15 的仿真结果作为分析对象，如图 6 所示。

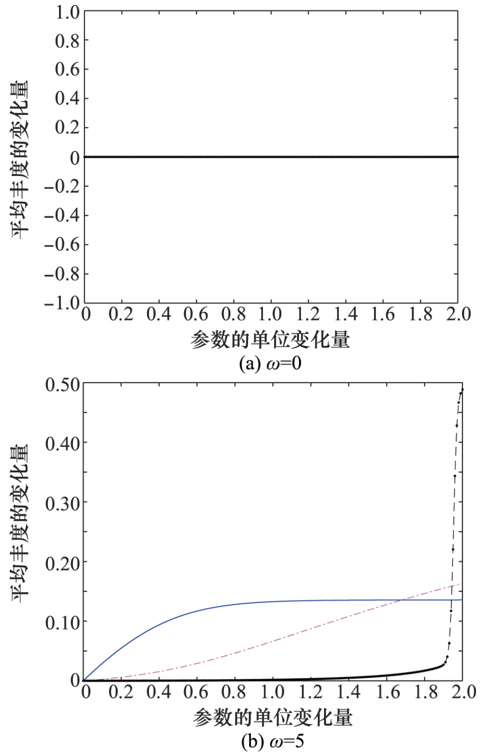

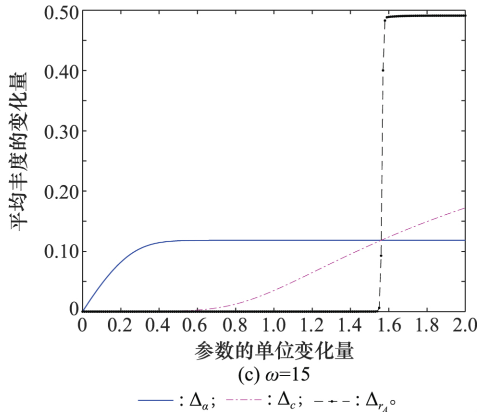

图 6　平均丰度与单位参数变化量间的关系

Fig. 6　Relationship beween average abundance and variation of unit parameters

由图 6(a)可知，当 $\omega=0$ 时，平均丰度 $X_A=0.5$，参数调控失效。

由图 6(b)和图 6(c)可知，$\omega\neq0$ 且 Δ 较小时（Δ 阈值与 ω 有关：$\Delta\approx1.70|_{\omega=5}$，$\Delta\approx1.53|_{\omega=15}$），改变 α、c 带来的平均丰度变化远大于 r_A。调控 α、c 较灵敏，调控 r_A 效果较差。

$\omega\neq0$ 且 Δ 较大时，对 r_A 的调控效果远大于 α、c。且 ω 越大，调整 r_A 越灵敏，即较小的单位变化量 Δ_{r_A} 引发较大幅度的平均丰度提升：$\Delta<X_A(\omega=5,\Delta_{r_A}=1.95)>\approx0.43$，$\Delta<X_A(\omega=20,\Delta_{r_A}=1.55)>\approx0.48$。

实际调控中，理想情况是同时增加合作单元的收益系数 r_A 和降低成本 c（对愿景水平 α 的调控仅具理论意义，实际中很难实施），然而针对战场上特定的使命任务，为保障作战效能的发挥，成本很难降低甚至会增加。因此，需要考虑 r_A、c 同时增加的情况。图 7 为 $\omega=1$ 时 r_A、c 同时增加时平均丰度的变化，c 增加了 50%，r_A 分别增加 69%和 73%。

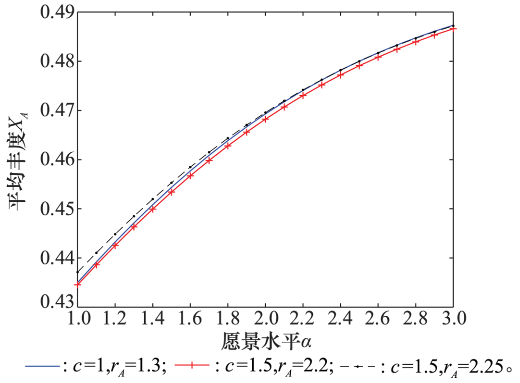

图 7　成本和收益系数同时增加时平均丰度的影响

Fig. 7　Influence of average abundance with the cost and benefit coefficient increasing simultaneously

由图 7 可知，只要 r_A 增加超过 73%，不仅可抵消成本 c 对平均丰度的不利影响，还可促进集群合作的发生。

然而，上述调控结果只能实现平均丰度有限范围的提升，并不能使平均丰度大于 0.5。根据图 4 和图 5 分析结

(C)1994-2020 China Academic Journal Electronic Publishing House. All rights reserved.　http://www.cnki.net

果，占优策略的转换（平均丰度的大幅提升）有赖于较大的选择强度 ω 和较大的单位变化量 Δ_{r_A}。为此，在 c 增加 50% 前提下，进一步增加 r_A，如图 8 所示，当 $r_A=2.52$ 且 $\omega\approx15$ 时，$X_A>0.5$；当 $r_A=2.65$ 且 $\omega\approx5$ 时，$X_A>0.5$。

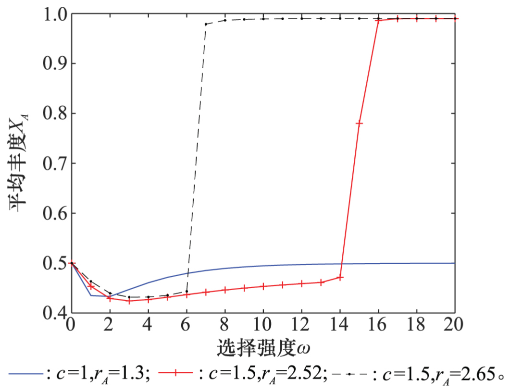

图 8　增加成本和收益系数实现占优策略转换

Fig. 8　Stragy alternation by increasing cost and benefit coefficient

收益系数 r_A 的增加意味着采取非合作策略的“搭便车”者将不再获取与合作者相同的收益回报，收益的降低将直接提升非合作者的策略更新概率 $P_{B\to A}$，从而使更多的单元趋向于合作。

在实际调控中，可以采取以下方法。

（1）尽可能提升合作者的收益系数 r_A。例如，借助先进管理手段，针对集群的每个作战单元，在任务执行前，可累计其历次行动的投资成本，在后续作战任务中，投资成本较高者将给予更多的物资弹药补给，或更高的物资弹药补给优先权。

（2）尽可能降低或维持单次行动的投资成本 c。例如，借助先进技术手段，提高作战平台的可靠性、生存能力，提高单位弹药的打击精度、毁伤威力等。

5　结　论

无人集群作战最大优势在于其单元自主性，在单一平台受损或通信受阻导致失去人为控制后，仍可有序协同作战。本文针对无人集群合作演化问题，建立了基于愿景驱动的多元公共品演化博弈模型，以“公地悲剧”为切入点，理论推导出模型的平均丰度函数，计算了 3 个参数（成本 c、收益系数 r、愿景水平 α）对平均丰度的影响。以无人集群火力打击为例，仿真发现，提高合作者的收益系数 r_A 和降低成本 c 可有效提升合作者的平均丰度，且在参数变化量较大时，r_A 不仅具备较高的调控灵敏度还可实现占优策略的转换。最后给出合理化建议，为相关理论向实际应用转化提供了初步和有意义的探索。

本文假设了集群中个体的无差异性，反映了在公共品演化博弈模型中愿景水平 α 的单一性，而在实际中，不同的个体（例如不同功能类型的作战单元）对愿景的需求可能不同。如何计算多种愿景共存下的平均丰度，并探究其合作演化机制，将是下一步的研究方向。

参考文献：

[1] BURMAOGLU S, SARITAS O. Changing characteristics of warfare and the future of military R&D[J]. Technological Forecasting and Social Change, 2017, 116: 151－161.

[2] FAN J R, LI D G, LI R P, et al. Analysis on MAV/UAV cooperative combat based on complex network[J]. Defence Technology, 2020, 16(1): 150－157.

[3] 全吉，储育青，王先甲．具有惩罚策略的公共物品博弈与合作演化[J]．系统工程理论与实践，2019，39(1)：141－149.
QUAN J, CHU Y Q, WANG X J. Public goods with punishment and the evolution of cooperation[J]. Systems Engineering-Theory & Practice, 2019, 39(1): 141－149.

[4] SMITH J M, PRICE G R. The logic of animal conflict[J]. Nature, 1973, 246(5427): 15－18.

[5] NOWAK M A. Evolving cooperation[J]. Journal of Theoretical Biology, 2012, 299: 1－8.

[6] NOWAK M A, SIGMUND K. Evolution of indirect reciprocity[J]. Nature, 2005, 437(6685): 1291－1298.

[7] LIEBERMAN E, HAUERT C, NOWAK M A. Evolutionary dynamics on graphs[J]. Nature, 2005, 433(7023): 312－316.

[8] NOWAK M A. Five rules for the evolution of cooperation[J]. Science, 2006, 314(5805): 1560－1563.

[9] 王先甲，全吉，刘伟兵．有限理性下的演化博弈与合作机制研究[J]．系统工程理论与实践，2011，31(1)：82－93.
WANG X J, QUAN J, LIU W B. Study on evolutionary games and cooperation mechanism within the framework of bounded rationality[J]. Systems Engineering-Theory & Practice, 2011, 31(1): 82－93.

[10] ZHANG L Y, YING L M, ZHOU J, et al. Fixation probabilities of evolutionary coordination games on two coupled populations[J]. Physical Review E, 2016, 94(3): 032307.

[11] TAKESUE H, OZAWA A, MORIKAWA S. Evolution of favoritism and group fairness in a co-evolving three-person ultimatum game[J]. Europhysics Letters, 2017, 118(4): 48002.

[12] WOLFF I. What are the equilibria in public-good experiments[J]. Economics Letters, 2017, 150: 83－85.

[13] 王先甲，夏可．多人雪堆演化博弈在愿景驱动规则下的扩展平均丰度函数[J]．系统工程理论与实践，2019，39(5)：1128－1137.
WANG X J, XIA K. Extended average abundance function of multi-player snowdrift evolutionary game under aspiration driven rule[J]. Systems Engineering-Theory & Practice, 2019, 39(5): 1128－1137.

[14] DU J M, WU B, WANG L. Aspiration dynamics in structured population acts as if in a well-mixed one[J]. Scientific Reports, 2015, 5: 1－7.

[15] TARNITA C E, OHTSUKI H, ANTAL T, et al. Strategy selection in structured populations[J]. Journal of Theoretical Biology, 2009, 259(3): 570－581.

[16] SUI X K, CONG R, LI K, et al. Evolutionary dynamics of N-person snowdrift game[J]. Physics Letters A, 2015, 379

(C)1994-2020 China Academic Journal Electronic Publishing House. All rights reserved.　http://www.cnki.net

(45－46)：2922－2934.
[17] PENA J, WU B, TRANLSEN A. Ordering structured populations in multiplayer cooperation games[J]. Journal of the Royal Society Interface, 2016, 13(114): 20150881.
[18] WU B, TRANLSEN A, GOKHALE C. Dynamic properties of evolutionary multi-player games in finite populations [J]. Games, 2013, 4(2): 182－199.
[19] DU J M, WU B, ALTROCK P M, et al. Aspiration dynamics of multi-player games in finite populations[J]. Journal of the Royal Society Interface, 2014, 11(94): 20140077.
[20] DU J M, WU B, WANG L. Aspiration dynamics and the sustainability of resources in the public goods dilemma[J]. Physics Letters A, 2016, 380(16): 1432－1436.
[21] DU J M, WU B, WANG L. Evolutionary game dynamics of multi-agent cooperation driven by self-learning[C] // Proc. of the 9th Asian Control Conference, 2013.
[22] WU B, ALTROCK P M, WANG L, et al. Universality of weak selection[J]. Physical Review E, 2010, 82(4): 046106.
[23] XU K Y, LI K, CONG R, et al. Cooperation guided by the coexistence of imitation dynamics and aspiration dynamics in structured populations[J]. Europhysics Letters, 2017, 117(4): 48002.
[24] WANG X J, GU C L, LV S J, et al. Evolutionary game dynamics of combining the Moran and imitation processes[J]. Chinese Physics B, 2019, 28(2): 1－13.
[25] CHEN Y S, YANG H X, GUO W Z. Aspiration-induced dormancy promotes cooperation in the spatial Prisoner's Dilemma games[J]. Physica A: Statistical Mechanics and its Applications, 2017, 469: 625－630.
[26] PLATKOWSKI T. Aspiration-based full cooperation in finite systems of players[J]. Applied Mathematics and Computation, 2015, 251: 46－54.
[27] **王先甲，刘佳. 具有外部性的合作博弈问题中的稳定的联盟结构**[J]. **系统工程理论与实践**, 2018, 38(5): 1173－1183.
WANG X J, LIU J. Stable coalition structures in cooperative game with externalities[J]. Systems Engineering-Theory & Practice, 2018, 38(5): 1173－1183.
[28] **王先甲，夏可. 愿景驱动、演化博弈与环境污染治理进路**[J]. **江汉论坛**, 2018, 7: 38－44.
WANG X J, XIA K. Aspiration driven, evolutionary game and environmental pollution control[J]. Jianghan Tribune, 2018, 7: 38－44.
[29] LIU X S, HE M F, KANG Y B, et al. Fixation of strategies with the Moran and Fermi processes in evolutionary games[J]. Physica A: Statistical Mechanics and its Applications, 2017, 484: 336－344.
[30] LIU X S, HE M F, KANG Y B, et al. Aspiration promotes cooperation in the prisoner's dilemma game with the imitation rule[J]. Physical Review E, 2016, 94(1): 012124.
[31] GRAHAM R L, KNUTH D E, PATASHNIK O. Concrete Mathematics[M]. 2nd ed. Massachusetts: Addison-Wesley Publishing Company, 1994.
[32] LI P P, KE J H, LIN Z Q, et al. Cooperative behavior in evolutionary snowdrift games with the unconditional imitation rule on regular lattices[J]. Physical Review E, 2012, 85(2): 021111.
[33] TAYLOR P D, JONKRT L B. Evolutionary stable strategies and game dynamics[J]. Mathematical Biosciences, 1978, 40(1－2): 145－156.
[34] SZABO G, TOKE C. Evolutionary prisoner's dilemma game on a square lattice[J]. Physical Review E, 1998, 58: 69－73.
[35] NOWAK M A, SIGMUND K. Evolitionary dynamics of biologival games[J]. Science, 2004, 303(5659): 793－799.
[36] **刘爱志. 基于演化博弈论的若干合作演化机制研究**[D]. **北京：北京科技大学**, 2018.
LIU A Z. Research on several evolutionary mechanisms of cooperation based on evolutionary game theory[D]. Beijing: Beijing University of Science and Technology, 2018.
[37] VAN K N G. Stochastic process in physics and chemistry[M]. North-Holland: Elsevier, 1992.
[38] GARDINER C W. Handbook of stochastic methods[M]. Berlin: Springer, 1985.

作者简介：

禹明刚(1986－)，**男，讲师，博士，主要研究方向为军事需求工程、体系工程、无人集群合作演化。**
E-mail：yuminggang8989@163.com
张东戈(1965－)，**通信作者，男，教授，博士研究生导师，硕士，主要研究方向为复杂军事系统分析、军事信息学、军事运筹学。**
E-mail：DongGeZhang@hotmail.com
康　凯(1986－)，**男，讲师，硕士，主要研究方向为复杂军事系统分析、集群作战。**
E-mail：36442181@qq.com
朱卫星(1978－)，**男，副教授，硕士研究生导师，博士，主要研究方向为复杂军事系统建模、评估。**
E-mail：329674406@qq.com

(C)1994-2020 China Academic Journal Electronic Publishing House. All rights reserved. http://www.cnki.net
