# Supplementary material for: A game-based approach for designing a collaborative evolution mechanism for unmanned swarms on community networks
Source: Sci Rep. 2022 Nov 7;12:18892. doi: 10.1038/s41598-022-22365-z (PMC9640601; doi:10.1038/s41598-022-22365-z)
Supplement: Supplementary file 1 — Supplementary Information. [file 41598_2022_22365_MOESM1_ESM.zip › Supporting material/4.í╢Strategy dominance condition of unmanned combat cluster based on multi-player public goods gameú¿╗∙╙┌╢α╘¬╣1⁄2╣▓╞╖╤▌╗»▓⌐▐─╡─╬▐╚╦╫≈╒╜╝»╚║▓▀┬╘╒╝╙┼╠⌡╝■ú⌐í╖.pdf]

第 43 卷　第 9 期　　系统工程与电子技术　　Vol. 43　No. 9
2021 年 9 月　　Systems Engineering and Electronics　　September 2021
文章编号：1001-506X(2021)09-2553-09　　网址：www. sys-ele. com

基于多元公共品演化博弈的无人作战集群策略占优条件

禹明刚[1,2]，何　明[1,*]，张东戈[1]，马子玉[1]，康　凯[1]
（1. 陆军工程大学指挥控制工程学院，江苏 南京 210007；2. 陆军工程大学通信工程学院，江苏 南京 210007）

摘　要：如何提高集群中合作者占比，是无人集群作战中自主协同的关键问题之一。基于多元公共品演化博弈框架，对无人作战集群策略占优条件进行了理论推导和特性分析。首先，给出无人集群自主协同需求，建立基于愿景驱动的多元演化博弈模型。然后，以平均丰度函数为出发点，理论推导多元演化博弈的合作策略占优条件。在此基础上，针对线性和带门限值的两类公共品博弈，仿真分析收益系数、愿景水平、门限值等参数对策略占优的影响，获取两类博弈模型的策略占优特性。最后，依据特性分析结果，给出实现无人集群合作策略占优的建议，为无人集群作战的机制设计提供决策支持。

关键词：无人集群作战；自主协同；多元公共品演化博弈；策略占优
中图分类号：TP 391　　**文献标志码**：A　　**DOI**：10. 12305/j. issn. 1001-506X. 2021. 09. 23

Strategy dominance condition of unmanned combat cluster based on multi-player public goods evolutionary game

YU Minggang[1,2], HE Ming[1,*], ZHANG Dongge[1], MA Ziyu[1], KANG Kai[1]
(*1. Institute of Command and Control Engineering, Army Engineering University, NanJing 210007, China;*
2. Institute of Communication Engineering, Army Engineering University, NanJing 210007, China)

Abstract: How to improve the proportion of cooperators is one of the key problems of autonomous cooperation in unmanned cluster operations. This research makes theoretical derivation and characteristic analysis on the strategy dominance condition of unmanned combat cluster based on multi-player public goods evolutionary game. It starts with the requirement analysis of autonomous collaboration in unmanned cluster firstly. And the evolutionary game model of multi-player public goods based on aspiration-driven dynamics is established. Then, the average abundance function and strategy dominance condition are constructed by theoretical derivation. Furthermore, the influence of multiplication factor, threshold and aspiration level on the strategy dominance of linear public goods game and threshold public goods game are simulated. Finally, the rationalization proposals to improve the proportion of cooperators and achieve strategy dominance are suggested according to the simulation results, and to provide decision-making support for the mechanism design of unmanned cluster operation.

Keywords: unmanned combat cluster; autonomous collaboration; multi-player public goods evolutionary game; strategy dominance

0　引　言

随着第 3 次人工智能浪潮的持续推进，由单体自主智能发展而来的群体演化智能，成为人工智能 2.0 的重要特征之一。尤其在军事领域，无人集群（陆战场无人车集群[1-3]、水面无人艇集群[4-6]、空域蜂群[7-10]）作战得到了

收稿日期：2020－07－20；修回日期：2021－02－24；网络优先出版日期：2021－05－26。
网络优先出版地址：http://kns. cnki. net/kcms/detail/11. 2422. TN. 20210525. 1518. 002. html
基金项目：国家自然科学基金（71901217）；军委科技委基础加强计划重点基础研究项目（2020-JCJQ-ZD-007）；国家重点研发计划（2018YFC0806900）；陆军工程大学军事理论创新工程项目（KYZYJQZL2101）资助课题
* 通讯作者.
引用格式：禹明刚，何明，张东戈，等. 基于多元公共品演化博弈的无人作战集群策略占优条件[J]. 系统工程与电子技术，2021，43(9)：2553-2561.
Reference format: YU M G, HE M, ZHANG D G, et al. Strategy dominance condition of unmanned combat cluster based on multi-player public goods evolutionary game[J]. Systems Engineering and Electronics, 2021, 43(9): 2553-2561.

(C)1994-2022 China Academic Journal Electronic Publishing House. All rights reserved.　http://www.cnki.net

前所未有的关注，美军已将无人集群作战列为一种能够改变作战规则的“颠覆性技术”。

目前，无人集群控制方式主要有集中控制和自主协同两类。前者依赖于地面站指令和无人机预编程，后者要求集群针对环境态势进行自主智能决策。在战场复杂电磁环境下，尤其当集群进入对方纵深之后，通信保障的展开面临极高难度，通信失效概率急剧上升[11]。此时，集中控制方式失效，无人集群必须依据对战双方情况、战场环境等，作出针对性的临机响应，依托集群内部的自组织、自协同，接续遂行军事任务。

在无人集群的自主协同进程中，资源的全局优化配置是必不可少的环节，且深刻影响自主协同效能的发挥。然而，在资源配置中，智能单元的个体利益诉求和集群全局作战需求，需要寻求一个平衡点。以集群火力打击任务为例，由于智能性的存在，每个打击单元均可独立决策，为了保证自身的战场生存能力，其将谨慎控制火力资源发射(投放)量。另一方面，在集群层面，单个打击单元提供的火力支持强度越大，越有利于集群整体作战效能的发挥。上述两者在需求上的矛盾性将催生公共资源悲剧的发生[12]。如何设计合理的集群自协同机制，避免矛盾冲突，无论是在集群控制基础领域还是现实演训/作战中，都是一项必须解决的难题。

集群自协同的本质在于解决个体间对立统一的关系，即求得收益的均衡。演化博弈理论[13-14]为解决集群自协同打开了一扇门。其中，公共物品演化博弈[15]为发掘集群的自组织机理、有效管控矛盾冲突奠定了一套理论框架。在该博弈过程中，研究如何提高合作者占比，并获取严格的合作策略占优条件，是解决公共资源悲剧，实现无人集群自主协同的重要前提。

哈佛大学 Nowak 教授团队[16-17]通过理论推导和模拟仿真，得到了基于模仿动态的多方博弈在弱选择强度下的策略占优条件。Antal 教授团队获取了两方博弈策略占优条件[18]，在此基础上，对 Nowak 结论进行拓展，得到了基于模仿动态的多方博弈在任何选择强度下的策略占优条件[19-20]。不同于 Nowak 教授团队，北京大学杜金铭团队将研究点从模仿动态[16,21]转向愿景驱动[22]策略更新机制下的策略占优条件研究。基于 TARNITA[23]的研究工作，借助于统计学及计算机仿真发现弱选择强度下平均丰度独立于愿景水平值这一规律[24]。进一步地，将理论成果拓展到多方博弈，获取了基于愿景驱动的多方博弈在弱选择强度下的策略占优条件[25-26]。

上述研究，为解决集群自组织自协同提供了很好的思路，打下了坚实基础。然而，在解决无人集群自主协同问题时，仍有两点需要引起注意：一是现有成果多面向一般性的演化博弈模型，尚未聚焦于公共物品博弈，而公共物品博弈恰恰是研究无人集群策略占优条件、解决公共资源悲剧的基础理论框架；二是尚未见军事应用研究成果，目前可见公共物品博弈在环境污染[27]、城市公共资源建设[28]、文化演进[29]等方面的应用，由于军事领域的特殊性及无人集群作战的新质性，鲜有成果可循。

前期，以集群的自主协同设计为目标牵引，通过理论推导，得到了多元公共品演化博弈的平均丰度函数，并仿真分析了相关参数对平均丰度的影响[30]。平均丰度是获取策略占优条件的根本依据，因此本研究在前期研究基础上，首先采用愿景驱动规则，基于多元演化博弈框架对无人集群合作演化建模。接下来，以平均丰度函数为出发点，理论推导多元演化博弈的合作策略占优条件。然后，针对线性及门限两类典型的公共物品博弈，给出策略占优条件并进行特性分析。最后，依据特性分析结果，给出避免公共资源悲剧，实现无人集群自主协同的建议。

1　军事需求分析

无人集群自主协同示意图如图 1 所示。

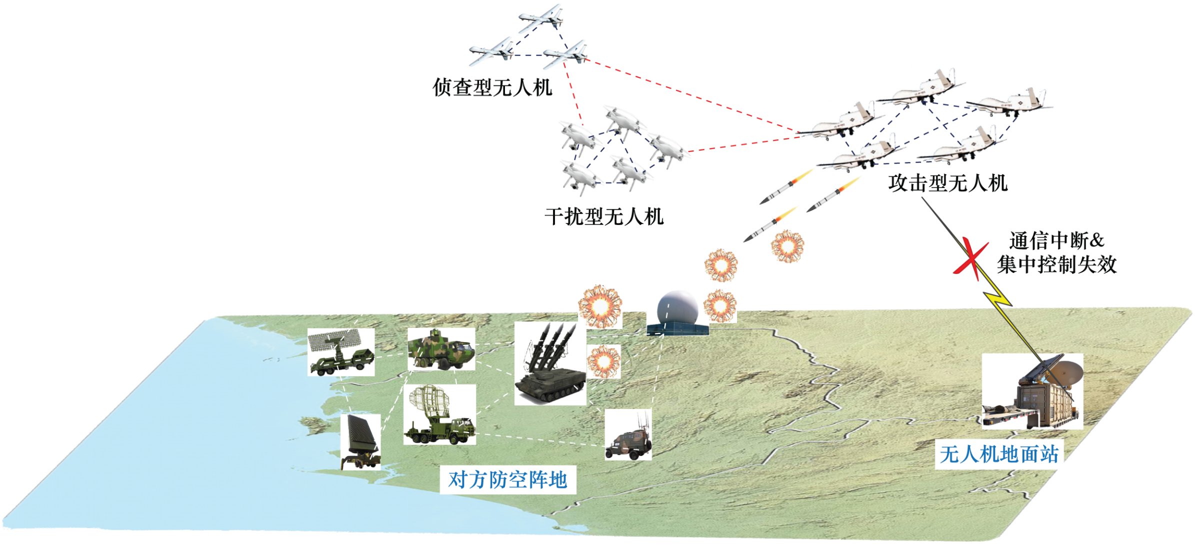

图 1　无人集群自主协同示意图

Fig. 1　Sketch map of autonomous cooperation of unmanned swarm

(C)1994-2022 China Academic Journal Electronic Publishing House. All rights reserved.　http://www.cnki.net

无人集群的自主协同，涉及 3 个关键问题：一是集群智能的涌现，二是信息网络的构建，三是协同机制的设计，三者共同构建起了无人集群自主协同行为产生的基础框架。三者之间的关系如图 2 所示（由于信息网络的构建不在本文讨论范围，因此做了淡化处理）。

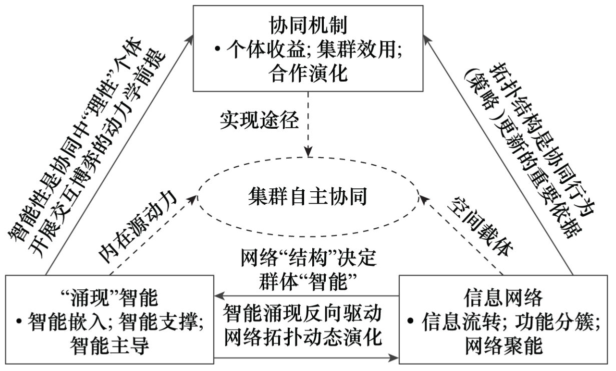

图 2　无人集群自主协同行为产生的基础框架

Fig. 2　Basic framework of autonomous cooperative behavior in unmanned swarm

其中，从个体到群体的智能涌现是集群自主性协同行为产生的内在源动力；信息网络是集群内部信息交互发生的空间，是自主协同行为的空间载体；协同机制则是集群自主协同得以实现的最终途径。下面分别就智能涌现和协同机制展开讨论。

1.1　智能涌现

智能性（包括个体的单体智能和群体涌现智能）是分布式自主控制方式对集群的必然要求。事实上，让无人集群按照预定方案执行军事任务，这一思路本身存在先天不足。复杂环境下，战场态势瞬息万变，若对单个无人平台实施微观管理，将导致通信等资源严重过载，即响应性的控制大量无人平台将超出人类目前的技术、认知、决策能力，大概率导致作战行动失败。因此，必须将更多的决策、行动权限前移给集群自治系统，使得无人平台能够独立协调自身决策以产生支持集群目标的行为。

同时，智能化作战制胜机理的内核即为智能、自主。美国国防科学委员会指出智能和自主能力是美军无人系统中的核心能力，并分析了智能和自主能力给无人机、无人地面系统、无人海上平台和无人太空系统带来的作战效益[31]。未来无人集群作战系统将具备更高的感知、分析、计划、决策和执行能力，并朝着战场态势自主感知、作战任务自主规划、作战行动自主实施，作战协同自主联动、作战效果自主评估的方向迈进。

目前来看，无人作战力量的发展路径也正是起始于人机互动的遥控式，经历人机结合的协作式，向人机共融的自主式方向发展[32−33]。可以预见，无人集群的智能涌现也将经历有人为主、无人为辅的智能嵌入到有人为辅、无人自主的智能支撑再到仿生自主、集群攻防的智能主导演进[34]。

1.2　协同机制

无人集群作战由于其体系的区域分布性，智能自主特性以及去中心化特性，集群内部必须基于信息网络，构建起有序的协同与合作，以确保良好的战场生存能力和任务完成能力。

具备智能性的单个无人平台在与其他平台的交互中，必然会计算评估其自身的能量、损耗、成本、行为代价等因素，以最大化其自身收益，此过程不可避免地伴随着个体间的竞争，导致个体收益与集群总效用最优上的偏离。因此，协同机制设计中的一类关键问题是如何保持个体收益与集群效用的一致。

良好的协同机制设计是破解个体收益与集群总效用间矛盾的关键。目前，在经典的多智能体系统（multi-agent system，MAS）理论[35]、复杂适应系统（complex adaptive systems，CAS）理论[36]和复杂网络理论[37]框架下，组分（个体）与系统（集体）在各自优化方向上的竞争和冲突问题，有待进一步深化研究。

不同于传统的优化问题，群体协同控制问题并非简单地可以通过选择某种行为，以提高所有个体的适应能力。更复杂的情况是，不同个体在交互过程中，由于其相互间直接的影响，各个个体利益的提高往往是相互冲突的。构建在多个体对立统一基础之上的博弈论恰恰为研究群体中多个体间的交互协同提供了一种有效的研究框架。

所有个体作为博弈的参与方，各种可选行为是博弈的策略集，前两者与各策略的对应收益一起构成博弈局势。个体通过评估周围个体及环境因素的影响，选择某种策略，并在重复博弈过程中，通过自适应学习来最大化自身及群体收益[38−39]。最终，借助于经典博弈的纳什均衡或演化博弈的演化稳定策略（evolutionary stable strategy，ESS）来揭示群体协同机理。

2　数学模型

首先，需要明确待解问题与理论框架间的关联关系，如表 1 所示。

表 1　概念映射关系

Table 1　Relationship between concepts

无人集群自主协同	多元公共品演化博弈
无人集群	混合均匀种群
自主协同所需公共资源（如弹药、通信等）	公共品
参与自主协同的多个无人平台	多元
单个无人平台	个体
作为研究对象的单个无人平台	焦点个体
平台愿意向集群贡献资源的合作行为	策略 A
平台不愿向集群贡献资源，而选择"搭便车"的非合作行为	策略 B
不同策略下，集群回馈给平台的公共资源	收益
无人平台间基于收益的策略转换	博弈
多轮博弈中，采用不同策略的平台在集群中占比动态变化	演化
多轮博弈后，占比稳定，博弈终止	演化稳定
演化稳定后，合作者（非合作者）在集群中占比的期望值	平均丰度
合作者在集群中平均丰度大于 0.5	合作策略占优

(C)1994-2022 China Academic Journal Electronic Publishing House. All rights reserved.　　http://www.cnki.net

2.1　多元演化博弈模型

本研究设定种群结构为混合均匀，种群规模为 N，每个个体均在有限策略集 $\{A,B\}$ 中实施选择和更新。随着演化进程的迭代滚动，A/B 类型的个体在种群 N 中数量（即比例）将实时调整，并最终平稳收敛到某一值附近，此时即为演化稳定。

将多元演化博弈过程提炼为 3 个主要环节，如图 3 所示。

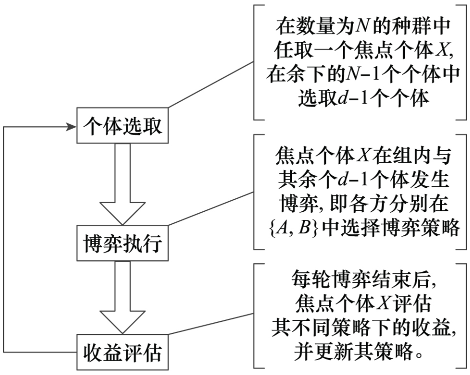

图 3　多元演化博弈过程

Fig. 3　Procedure of multiple evolutionary game

上述过程重复推进，直到演化稳定状态。依据超几何分布的数学意义[40]，A 类型焦点个体 X 在某轮博弈中的期望收益为

$$\pi_A(i) = \sum_{k=0}^{d-1} \frac{C_{i-1}^{k} C_{N-i}^{d-k-1}}{C_{N-1}^{d-1}} a_k \tag{1}$$

同理，$P_B(N,i;d,k)=C_i^k C_{N-i-1}^{d-k-1}/C_{N-1}^{d-1}$，一个 B 类型焦点个体 X 在某轮博弈中的期望收益为

$$\pi_B(i) = \sum_{k=0}^{d-1} \frac{C_{i}^{k} C_{N-i-1}^{d-k-1}}{C_{N-1}^{d-1}} b_k \tag{2}$$

具体推导过程可参见文献[30]，限于篇幅此处不再展开。

2.2　愿景驱动动态

演化博弈理论框架下，策略更新机制总体上可划分为两大分支：模仿动态[21]和愿景驱动动态[41-43]。现有成果表明，无论在囚徒困境博弈还是公共物品博弈中，愿景驱动的动态机制相比于传统模仿动态，更能提高平均丰度值，进而促进合作[44-45]。在愿景驱动规则下，焦点个体从 A 类型更新为 B 类型的概率为

$$P_{A\to B}=\frac{1}{1+\mathrm{e}^{\omega(\pi_A(i)-\alpha)}} \tag{3}$$

式中：参数 α 反映了个体 X 的愿景高低；ω 为调节系数，其可调节项 $\pi_A-\alpha$ 对 $P_{A\to B}$ 的决定程度。若 $\pi_A-\alpha=0$，即 $P_{A\to B}=1/2$，那么 i 对于两策略具有同等的倾向性；若 $\pi_A-\alpha>0$（即 i 的收益超出愿景值），那么 $P_{A\to B}<1/2$，此时 i 对于 A 策略具有更高的倾向性；若 $\pi_A-\alpha<0$（即 i 的收益不及愿景值），那么 $P_{A\to B}>1/2$，此时 i 对于 B 策略具有更高的倾向性。

同理，焦点个体从 B 类型更新为 A 类型的概率为

$$P_{B\to A}=\frac{1}{1+\mathrm{e}^{\omega(\pi_B(i)-\alpha)}} \tag{4}$$

在任意一种策略更新机制下，每一轮演化博弈进程里 A 类型个体的数量变动存在 3 种情况：① 数量减少 1 个，$i\to i-1$；② 数量增加 1 个，$i\to i+1$；③ 数量保持不变，$i\to i$。

根据式(3)和式(4)可得对应的转移概率：

$$P(i\to i-1)=T_i^-=\frac{i}{N}\frac{1}{1+\mathrm{e}^{\omega(\pi_A(i)-\alpha)}} \tag{5}$$

$$P(i\to i+1)=T_i^+=\frac{N-i}{N}\frac{1}{1+\mathrm{e}^{\omega(\pi_B(i)-\alpha)}} \tag{6}$$

$$P(i\to i)=1-T_i^- - T_i^+ \tag{7}$$

3　策略占优规则

本节首先给出平均丰度的定义，根据定义给出其数学表达式，进而基于平均丰度函数的一阶泰勒展开式，推导出合作策略占优规则。

3.1　平均丰度

定义 1　平均丰度

设集群中 A 型作战单元数量为 j，比例 j/N 为随机变量，令 $\nu(j)$ 为 j/N 的概率分布，则定义 j/N 的期望值为集群中 A 型作战单元的平均丰度。

由上述定义易知，合作策略 A 的平均丰度 $<X_A(j)>$ 可表示为

$$<X_A(j)>=\sum_{j=0}^{N}\frac{j}{N}v(j) \tag{8}$$

平均丰度计算的关键是确定随机变量的概率分布 $\nu(j)$。对于无吸收态的马尔可夫链，$\nu(j)$ 即为马尔可夫链的平稳分布 $\varphi_j\ (j\in[0,N])$，而 φ_j 满足细节平衡条件[46-48]：

$$\varphi_j T_j^+=\varphi_{j+1}T_{j+1}^- \tag{9}$$

对式(9)进行归纳分析易得

$$\varphi_j=\frac{\prod_{i=0}^{j-1}T_i^+}{\prod_{i=1}^{j}T_i^-}\varphi_0=\prod_{i=0}^{j-1}h(i)\varphi_0,\ j\geqslant 1 \tag{10}$$

式中：$h(i)=T_i^+/T_{i+1}^-$。由于平稳分布 φ_j 满足 $\sum_{j=0}^{N}\varphi_j=1$，因此：

$$\sum_{j=0}^{N}\varphi_j=\varphi_0+\sum_{j=1}^{N}\prod_{i=0}^{j-1}h(i)\varphi_0=1 \tag{11}$$

由此解出 $\varphi_0=\dfrac{1}{1+\sum_{j=1}^{N}\prod_{i=0}^{j-1}h(i)}$，带入式(10)，得

$$\varphi_j=\frac{\prod_{i=0}^{j-1}h(i)}{1+\sum_{j=1}^{N}\prod_{i=0}^{j-1}h(i)},\ j\geqslant 1 \tag{12}$$

将式(12)代入式(8)可得策略 A 的平均丰度展开式：

$$<X_A(j)>=\sum_{j=0}^{N}\frac{j}{N}\varphi_j=\sum_{j=1}^{N}\frac{j}{N}\frac{\prod_{i=0}^{j-1}h(i)}{1+\sum_{j=1}^{N}\prod_{i=0}^{j-1}h(i)} \tag{13}$$

其中，

$$h(i)=\frac{T_i^+}{T_{i+1}^-}=\frac{(N-i)(1+\mathrm{e}^{\omega(\pi_A(i+1)-\alpha)})}{(i+1)(1+\mathrm{e}^{\omega(\pi_B(i)-\alpha)})} \tag{14}$$

3.2　策略占优条件

定义 2　策略占优

无人集群作战中，当集群内部博弈达到演化稳定状态时，

(C)1994-2022 China Academic Journal Electronic Publishing House. All rights reserved.　http://www.cnki.net

若某策略的平均丰度值大于 0.5，则称该策略为占优策略。

因此，合作策略占优即为

$$<X_A(j)> = \sum_{j=0}^{N} \frac{j}{N}\varphi_j > \frac{1}{2} \tag{15}$$

对式(15)的 φ_j 做一阶泰勒展开：

$$\tilde{\omega}_j \approx \tilde{\omega}_j(\omega)|_{\omega=0} + \omega\left[\frac{\partial}{\partial \omega}\varphi_j\right]_{\omega=0} \tag{16}$$

$$\frac{\partial}{\partial \omega}\varphi_j = \frac{\left(\dfrac{\prod_{i=0}^{j-1} T_i^+}{\prod_{i=1}^{j} T_i^-}\right)'\left(1+\sum_{k=0}^{N-1}\dfrac{\prod_{i=0}^{k} T_i^+}{\prod_{i=1}^{k+1} T_i^-}\right)}{\left(1+\sum_{k=0}^{N-1}\dfrac{\prod_{i=0}^{k} T_i^+}{\prod_{i=1}^{k+1} T_i^-}\right)^2} - \frac{\left(\dfrac{\prod_{i=0}^{j-1} T_i^+}{\prod_{i=1}^{j} T_i^-}\right)\left(1+\sum_{k=0}^{N-1}\dfrac{\prod_{i=0}^{k} T_i^+}{\prod_{i=1}^{k+1} T_i^-}\right)'}{\left(1+\sum_{k=0}^{N-1}\dfrac{\prod_{i=0}^{k} T_i^+}{\prod_{i=1}^{k+1} T_i^-}\right)^2} \tag{17}$$

式中：

$$(T_i^+)' = \frac{N-i}{N}\frac{(e^{\omega[\pi_B(i)-\alpha]})[\alpha-\pi_B(i)]}{\{1+e^{\omega[\pi_B(i)-\alpha]}\}^2} \tag{18}$$

$$(T_i^-)' = \frac{i}{N}\frac{(e^{\omega[\pi_A(i)-\alpha]})[\alpha-\pi_A(i)]}{\{1+e^{\omega[\pi_A(i)-\alpha]}\}^2} \tag{19}$$

由于 $\omega \to 0$，因此：

$$(T_i^+)'|_{\omega=0} = \frac{N-i}{4N}[\alpha-\pi_B(i)] \tag{20}$$

$$(T_i^-)'|_{\omega=0} = \frac{i}{4N}[\alpha-\pi_A(i)] \tag{21}$$

$$\left.\left(\prod_{i=0}^{j-1} T_i^+\right)\right|_{\omega=0} = \prod_{i=0}^{j-1}\frac{N-i}{2N} = \frac{N!}{(N-j)!(2N)^j} \tag{22}$$

$$\left.\left(\prod_{i=1}^{j} T_i^-\right)\right|_{\omega=0} = \prod_{i=1}^{j}\frac{i}{2N} = \frac{j!}{(2N)^j} \tag{23}$$

$$\left.\left[\sum_{i=0}^{j-1}(T_i^+)\left(\prod_{k=0,k\neq i}^{j-1} T_k^+\right)\right]\right|_{\omega=0} = \frac{N!\sum_{i=0}^{j-1}[\alpha-\pi_B(i)]}{2(N-j)!(2N)^j} \tag{24}$$

$$\left.\left[\sum_{i=1}^{j}(T_i^-)\left(\prod_{k=1,k\neq i}^{j} T_k^-\right)\right]\right|_{\omega=0} = \frac{j!\sum_{i=1}^{j}[\alpha-\pi_A(i)]}{2(2N)^j} \tag{25}$$

将式(20)～式(25)代入式(16)，得

$$\frac{\partial}{\partial \omega}\varphi_j|_{\omega=0} = \frac{C_N^j}{2^{(2N+1)}}2^N\sum_{k=1}^{j}[\pi_A(k)-\pi_B(k-1)] - \frac{C_N^j}{2^{(2N+1)}}\sum_{k=1}^{N}C_N^k\sum_{i=1}^{k}[\pi_A(i)-\pi_B(i-1)] \tag{26}$$

将式(20)和式(21)代入式(26)得

$$\pi_A(i)-\pi_B(i-1) = \sum_{k=0}^{d-1}\frac{C_{i-1}^k C_{N-i}^{d-1-k}}{C_{N-1}^{d-1}}(a_k-b_k) \tag{27}$$

由于：

$$\sum_{j=0}^{N}\frac{j}{N}\varphi_j(0) = \sum_{j=0}^{N}\frac{j}{N}\frac{C_N^j}{2^N} = \frac{1}{2} \tag{28}$$

策略占优条件即等价于：

$$\sum_{j=0}^{N}\frac{j}{N}\omega\left[\frac{\partial\varphi_j}{\partial\omega}\right]_{\omega=0} > 0 \tag{29}$$

由数学推导易得

$$\sum_{i=1}^{N}C_{i-1}^k C_{N-i}^{d-1-k}\sum_{j=1}^{N}(2j-N)C_N^j = 2^{N-d}NC_{N-1}^{d-1}C_{d-1}^k \tag{30}$$

结合式(24)和式(25)，可得

$$\frac{\omega}{4(2^d)}\sum_{k=0}^{d-1}[C_{d-1}^k(a_k-b_k)] > 0 \tag{31}$$

因此，合作策略占优条件为

$$\sum_{k=0}^{d-1}[C_{d-1}^k(a_k-b_k)] > 0 \tag{32}$$

式(32)即为合作策略占优条件，该条件适用于任何多方双策略博弈。传统模仿动态的策略占优条件，完全取决于不同策略收益差的加和[19]，$\sum_{k=0}^{d-1}(a_k-b_k)>0$。在愿景驱动模式下，为策略收益差增加了额外权重，$\sum_{k=0}^{d-1}[C_{d-1}^k(a_k-b_k)]>0$，因此，集群中合作策略持有者数量 k 将变得尤为重要。由于组合数 C_{d-1}^k 的对称特性，当 k 取 d 的中间值(即集群中合作者与非合作者数量基本均等)时，C_{d-1}^k 对策略占优的影响程度最大。

本节通过严格数学推导，得出了多元演化博弈策略占优条件，为下一节两类公共物品博弈特性分析，提供了理论依据。

4　演化博弈分析

本节对线性和带门限值的两类公共品博弈进行分析，获取其策略收益，仿真策略占优特性，并最终为集群自主协同机制设计给出合理化建议。

4.1　线性公共品博弈

在线性公共品博弈中，当 X 选择合作策略 A，则群组可获得的总资源量为 $kc+c$，成本增值后的总获益为 $r(kc+c)$，因此易得单体获益为 $r(kc+c)/d$，然而因为 X 最初有 c 的投资，则 X 净获益可表征为 $r(kc+c)/d-c$。另一种情况，X 采取了 B 策略，对应地，上述几个参数分别变化为 kc、rkc、rkc/d、rkc/d。a_k 与 b_k 具体形式可表征为

$$a_k = \frac{r(kc+c)}{d} - c \tag{33}$$

$$b_k = \frac{rkc}{d} \tag{34}$$

收益矩阵如表 2 所示。

表 2　线性公共品博弈收益矩阵

Table 2　Pay-off matrix of liner public goods game

策略	$d-1$	…	k	…	1	0
A	$rc-c$	…	$r(kc+c)/d-c$	…	$2rc/d-c$	$rc/d-c$
B	$r(d-1)c/2$	…	rkc/d	…	rc/d	0

(C)1994-2022 China Academic Journal Electronic Publishing House. All rights reserved.　http://www.cnki.net

由于 $a_k - b_k = c(r/d-1)$，且一般假设 $1<r<d$ 成立，根据式(32)考察线性公共品博弈策略占优情况：

$$\sum_{k=0}^{d-1}[C_{d-1}^{k}(a_k-b_k)] = 2^{d-1}c\left(\frac{r}{d}-1\right) < 0 \quad (35)$$

因此，线性公共品博弈为非合作占优博弈，即演化均衡时，集群中合作策略为非占优策略，非合作者将占据主导。为分析该博弈策略占优特性，取 $\alpha=1$、$N=100$、$c=1$，仿真分析选择强度 ω、收益系数 r、愿景水平 α 对合作策略平均丰度 X_A 的影响，以期总结规律，为无人集群合作策略占优管控提供参考。ω、r 与 X_A 的关系曲线如图 4 所示。

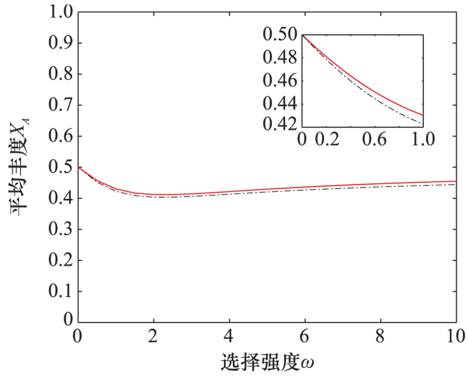

(a) 固定收益系数下的X_A

(a) X_A under fixed reward coefficient

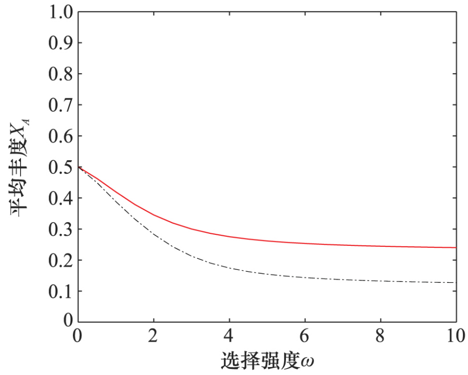

(b) 增加收益系数对X_A的影响

(b) Influence of increasing reward coefficient on X_A

——: d=10, r=2; -·-·-: d=20, r=2。

图 4　线性公共品博弈中选择强度、收益系数与 X_A 的关系

Fig. 4　Relationship between selection intensity, reward coefficient and X_A in linear public goods game

图 4(a)中，d 分别取 10 和 20，$r=2$。在选择强度 $\omega=0$ 时，平均丰度 $X_A==0.5$；在选择强度较小时(如图 4(a)小面板所示)，合作者的愿景难以满足，因此大量合作者转换策略，平均丰度出现下降趋势。随着选择强度增加，平均丰度略有提升，将逐渐稳定在 0.45 左右。图 4(b)中，d 分别取 10 和 20，$r=d/2$。相比于图 4(a)，图 4(b)中收益系数 r 的增加(r 由 2 分别增加到 5 和 10)，使得平均丰度产生递减，且 r 增加幅度越大平均丰度降幅越大：$X_A(\omega)|_{r=10}$ $X_A(\omega)|_{r=5}$，这是由于同时增加集群中合作单元和非合作单元的收益，将会使得“搭便车”现象更加严重，集群中大量作战单元转变为非合作者。

结论 1　线性公共物品博弈中，在成本 c、愿景水平 α 保持不变的情况下，合作者平均丰度将在弱选择强度($\omega\to 0$)和较小收益系数处保持相对较高水平。对于弱选择强度对合作的促进作用，已在生物遗传、分子进化、文化演进等领域得到现实验证[48-50]，尽管目前还不清楚其作用机理。

因此，在线性公共品博弈模型下的无人集群作战管控中，虽然合作为非占优策略，然而可通过为作战集群预设 ω 和 r 较低参数值，弱化两者对策略更新的影响，以最大限度提升集群中合作者平均丰度，促进集群合作的发生。

此外，还仿真了愿景水平 α 与 X_A 的关系曲线，ω 分别取 0、5、10、15、20，$c=1$，$r=1.1$。关系曲线如图 5 所示。

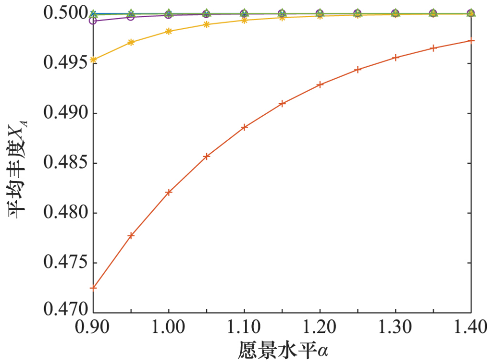

—×—: ω=0; —+—: ω=5; —*—: ω=10; —○—: ω=15; —△—: ω=20。

图 5　平均丰度与愿景水平间的关系曲线

Fig. 5　Relationship between average abundance and aspiration level

由图 5 可见，随愿景水平 α 的增加，X_A 呈增加趋势，这表明愿景水平的提高，使得非合作收益更难以达到其期望水平。由式(4)可知，策略更新概率 $P_{B\to A}$ 增加，更多的非合作者转变为合作者。当 α 足够高时，在任何选择强度下，$\lim_{\alpha\to\infty} X_A = 1/2$ 将成立。

结论 2　线性公共物品博弈中，在成本 c、收益系数 r 保持不变的情况下，合作者平均丰度将在较大愿景水平处保持相对较高水平。

因此，在线性公共品博弈模型下的无人集群作战管控中，可通过为集群预设较高 α 参数值，增加集群由非合作转变为合作策略的概率，以最大限度提升集群中合作者平均丰度，促进集群合作的发生。

4.2　门限公共品博弈

在带门限值的公共品博弈中，只有当群组中合作策略持有者总数量不低于门限值 m 时，个体才会获得收益。当 $k\geqslant m$，且 X 选择合作策略 A，则群组可获得的总资源量为 $kc+c$，成本增值后的总获益为 $r(kc+c)$，因此易得单体获益为 $r(kc+c)/d$。另一种情况，X 采取了 B 策略，对应地，上述几个参数分别变化为 kc、rkc、rkc/d、$rkc/d+c$。因此，a_k 与 b_k 具体形式为

$$a_k = \frac{r(kc+c)}{d} \quad (36)$$

$$b_k = \frac{rkc}{d} + c \quad (37)$$

收益矩阵如表 3 所示。

(C)1994-2022 China Academic Journal Electronic Publishing House. All rights reserved.　　http://www.cnki.net

表 3　门限公共品博弈收益矩阵

Table 3　Incme matrix of public goods game with threshold

策略	$d-1$	…	k	…	$m-1$	…	0
A	rc	…	$r(kc+c)/d$	…	mrc/d	…	0
B	$r(d-1)c/d+c$	…	$rkc/d+c$	…	0	…	0

因此：

$$\sum_{k=0}^{d-1}[C_{d-1}^{k}(a_k-b_k)]=\sum_{k=m}^{d-1}C_{d-1}^{k}\left(\frac{rc}{d}-c\right)+C_{d-1}^{m-1}\frac{mrc}{d} \tag{38}$$

不同于线性公共品博弈，式(38)并无明显的策略占优特征。接下来，试图通过计算与仿真，比较愿景驱动与模仿动态两类策略占优条件的严苛程度。

令 $\sum_{k=0}^{d-1}[C_{d-1}^{k}(a_k-b_k)]>0$，则由式(38)可进一步得到策略占优条件为

$$r>\frac{d\sum_{k=m}^{d-1}C_{d-1}^{k}}{\sum_{k=m}^{d-1}C_{d-1}^{k}+mC_{d-1}^{m-1}} \tag{39}$$

令 $t=\frac{d\sum_{k=m}^{d-1}C_{d-1}^{k}}{\sum_{k=m}^{d-1}C_{d-1}^{k}+mC_{d-1}^{m-1}}$，对其做等价变换：

$$t=\frac{d(\sum_{k=m}^{d-1}C_{d-1}^{k}+mC_{d-1}^{m-1})-dm\,C_{d-1}^{m-1}}{\sum_{k=m}^{d-1}C_{d-1}^{k}+mC_{d-1}^{m-1}}=$$

$$d-m\frac{(d-m)C_{d-1}^{m-1}+mC_{d-1}^{m-1}}{\sum_{k=m}^{d-1}C_{d-1}^{k}+mC_{d-1}^{m-1}}$$

则式(39)等价于：

$$r>d-m\frac{(d-m)C_{d-1}^{m-1}+mC_{d-1}^{m-1}}{\sum_{k=m}^{d-1}C_{d-1}^{k}+mC_{d-1}^{m-1}} \tag{40}$$

而模仿动态策略占优条件 $\sum_{k=0}^{d-1}(a_k-b_k)>0$，则由式(36)和式(37)可进一步得到：

$$r>d-m \tag{41}$$

比较式(40)与式(41)可以得到以下结论。

(1) 当 m 较大时，根据组合数性质，式(40)中，$(d-m)C_{d-1}^{m-1}>\sum_{k=m}^{d-1}C_{d-1}^{k}$ 成立。因此，相比于式(41)，式(40)更易得到满足，即愿景驱动比模仿动态的策略占优条件更宽松。也意味着在愿景驱动规则下，合作策略占优需要的回报更少(更低的收益系数 r)。

(2) 当 m 较小时，相反地，式(40)中，$(d-m)C_{d-1}^{m-1}<\sum_{k=m}^{d-1}C_{d-1}^{k}$ 成立。因此，相比于式(40)，式(41)更易得到满足，即愿景驱动比模仿动态的策略占优条件更苛刻。模仿动态比愿景驱动，更利于促进集群中合作现象的发生。

为分析愿景驱动下，门限公共品博弈策略占优特性，取 $\alpha=1$、$N=100$、$c=1$、$d=10$，仿真分析不同的门限值 m 及收益系数 r 对合作策略占优的影响。m、r 与 X_A 的关系曲线如图 6 所示。

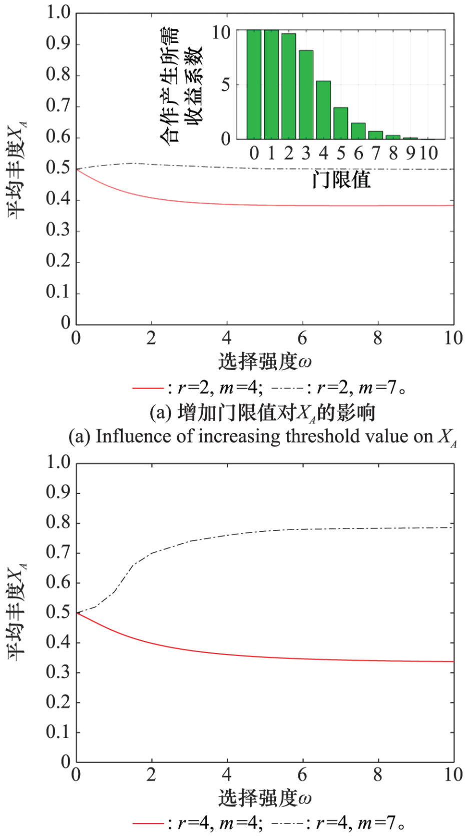

(a) 增加门限值对X_A的影响

(a) Influence of increasing threshold value on X_A

(b) 相同收益系数下门限值对X_A的影响

(b) Influence of threshold value on X_A under the

图 6　门限公共品博弈中门限值、收益系数与 X_A 的关系

Fig. 6　Relationshiop between threshold value, reward coefficient and X_A in public goods game with threshold

由图 6(a)可知，在 $r=2$ 时，当门限值 m 由 4 提升到 7，合作策略的平均丰度 X_A 不仅相应地完成了提升，而且实现了占优策略的转换(平均丰度由 $X_A|_{m=4}<0.5$ 转换为 $X_A|_m=7>0.5$，占优策略由 B 转换为 A)。图 6(a)右上角面板给出了几组门限值 m 与收益系数 r 间的关系，随门限值增加，合作策略占优时所需的收益系数越来越小(即所需的回报越来越少)。相比于图 6(a)，在图 6(b)中，m 仍然取 4 与 7，然而由于 r 由 2 增加为 4，因此在相同的选择强度和门限值下有 $X_A|_{m=4,r=4}>X_A|_{m=4,r=2}$ 和 $X_A|_{m=7,r=4}>X_A|_{m=7,r=2}$。

结论 3　门限公共物品博弈中，在成本 c、愿景水平 α 保持不变的情况下，较高的门限值能够促进合作，即使在较低的收益系数下；且在相同的门限值下，较高的收益系数更利于合作的产生。

因此，在门限公共品博弈模型下的无人集群作战管控中，可通过同时提高门限值 m 及收益系数 r，以发挥愿景驱动在促进集群合作中的优势，实现集群中合作策略占优目的。

在实际的无人集群管控中，依据第 2 节提出的无人集群演化博弈模型及愿景驱动动态，为无人集群预设自主协同规则 R_C。另外，针对具体作战场景，依据本研究所获结论 1 至结论 3，预设成本 c、愿景水平 α、收益系数 r、门限值

(C)1994-2022 China Academic Journal Electronic Publishing House. All rights reserved.　　http://www.cnki.net

m 等参数调整规则(r_1-r_3)。当地面控制站通信中断后,无人集群可根据预设规则临机作出有效响应,实现集群中合作策略的占优,以持续完成既定军事任务。

例如,在实际作战中,无人机的愿景水平 α 一般为定值,且弹药、通信等作战成本 c 难以进一步压缩。此时,无人集群可在 R_C 框架内依据 r_3 自动为协同进程设置较高的门限值 m 及收益系数 r,以此提升集群稳定时(对应于演化稳定的 ESS 状态)合作者的占比,促成集群中合作行为的涌现及合作策略占优的实现。

5 结 论

无人集群的自主协同是目前军事领域新质作战力量和颠覆性技术研究的焦点。自主协同中一个关键问题是,如何设计合理机制,提高作战集群中合作者比例,以保证集群的整体作战效能。本文首先建立了基于愿景驱动的多元演化博弈模型,然后理论推导出模型的平均丰度函数及策略占优条件,在此基础上,对线性和带门限值的两类公共品博弈进行数理推导并仿真分析选择强度 ω、收益系数 r、愿景水平 α 和门限值 m 对策略占优的影响,获取两类博弈模型的策略占优特性,为无人集群作战的机制设计提供辅助决策。

本研究中,假设了集群结构的混合均匀性,未考虑结构对策略占优特性的影响,而在现实战场环境中,作战平台通过物理/信息链接从而形成特定的网络结构。下一步课题组将引入复杂网络思想,计算特定网络结构下的无人集群演化博弈及策略占优特性。

参考文献

[1] XU P, DHERBOMEZ G, HERY E, et al. System architecture of a driverless electric car in the grand cooperative driving challenge[J]. IEEE Intelligent Transportation Systems Magazine, 2018, 10(1): 47-59.

[2] LIANG H, QIANG H, FENG Z. Research on capability characteristics modeling and cooperative fire strike planning for unmanned ground vehicles[C]//Proc. of the 2nd International Conference on Artificial Intelligence and Big Data, 2019: 136-141.

[3] ZOTO J, MUSCI M A, KHALIQ A, et al. Automatic path planning for unmanned ground vehicle using UAV imagery[C]//Proc. of the International Conference on Robotics in Alpe-Adria Danube Region,2019: 223-230.

[4] MA Y, HU M, YAN X. Multi-objective path planning for unmanned surface vehicle with currents effects[J]. ISA transactions, 2018, 75: 137-156.

[5] SONG R, LIU Y U, BUCKNALL R. Smoothed A* algorithm for practical unmanned surface vehicle path planning[J]. Applied Ocean Research, 2019, 83: 9-20.

[6] ZHOU Q, LI B F,ZHAN Z J. Research on the key technologies of unmanned cluster to sea combat[C]//Proc. of the Global Intelligence Industry Conference,2018.

[7] GILES K, GIAMMARCO K. A mission-based architecture for swarm unmanned systems[J]. Systems Engineering, 2019, 22(3): 271-281.

[8] KHAWAJA W, GUVENC I, MATOLAK D W, et al. A survey of air-to-ground propagation channel modeling for unmanned aerial vehicles[J]. IEEE Communications Surveys & Tutorials, 2019,21(3):2361-2391.

[9] SHAKERI R, Al-GARADI M A, BADAWY A, et al. Design challenges of multi-UAV systems in cyber-physical applications: a comprehensive survey, and future directions[J]. IEEE Communications Surveys & Tutorials, 2019,21(4):3340-3385.

[10] DOLICANIN E, FETAHOVIC I, TUBA E, et al. Unmanned combat aerial vehicle path planning by brain storm optimization algorithm[J]. Studies in Informatics and Control, 2018, 27(1): 15-24.

[11] FAN J R, LI D G, LI R P, et al. Analysis on MAV/UAV cooperative combat based on complex network[J]. Defense Technology, 2020, 16(1): 154-161.

[12] 全吉, 储育青, 王先甲. 具有惩罚策略的公共物品博弈与合作演化[J]. 系统工程理论与实践, 2019, 39(1): 141-149.
QUAN J, CHU Y Q, WANG X J. Public goods with punishment and the evolution of cooperation[J]. Systems Engineering-Theory & Practice, 2019, 39(1): 141-149.

[13] SMITH J M, PRICE G R. The logic of animal conflict[J]. Nature,1973, 246(5427):15-18.

[14] NOWAK M A, SIGMUND K. Evolution of indirect reciprocity[J]. Nature, 2005, 437(6685): 1291-1298.

[15] WOLFF I. What are the equilibria in public-good experiments?[J]. Economics Letters, 2017, 150:83-85.

[16] NOWAK M A, SASAKI A, TAYLOR C, et al. Emergence of cooperation and evolutionary stability in finite populations[J]. Nature, 2004, 428(6983): 646-650.

[17] SU Q, MCAVOY A, WANG L, et al. Evolutionary dynamics with stochastic game transitions[EB/OL]. [2020-07-15]. http://arxiv.org/abs/1905.10269.

[18] ANTAL T, NOWAK M A, TRAULSEN A. Strategy abundance in 2×2 games for arbitrary mutation rates[J]. Journal of Theoretical Biology, 2009, 257(2): 340-344.

[19] GOKHALE C S, TRAULSEN A. Evolutionary games in the multiverse[J]. Proceedings of the National Academy of Sciences, 2010, 107(12): 5500-5504.

[20] HINDERSIN L, WU B, TRAULSEN A, et al. Computation and simulation of evolutionary game dynamics in finite populations[J]. Scientific Reports, 2019, 9(1): 6946.

[21] WANG X J, GU C L, LV S J, et al. Evolutionary game dynamics of combining the Moran and imitation processes[J]. Chinese Physics B, 2019, 28(2): 94-105.

[22] ROCA C P, HELBING D. Emergence of social cohesion in a model society of greedy, mobile individuals[J]. Proceedings of the National Academy of Sciences, 2011, 108(28): 11370-11374.

[23] TARNITA C E, OHTSUKI H, ANTAL T, et al. Strategy selection in structured populations[J]. Journal of Theoretical Biology, 2009, 259(3): 570-581.

[24] DU J M, WU B, WANG L. Aspiration dynamics in structured population acts as if in a well-mixed one[J]. Scientific Reports,

(C)1994-2022 China Academic Journal Electronic Publishing House. All rights reserved. http://www.cnki.net

2015, 5(1): 8014.
[25] DU J M, WU B, ALTROCK P M, et al. Aspiration dynamics of multi-player games in finite populations[J]. Journal of the Royal Society Interface, 2014, 11(94): 20140077.
[26] DU J M, WU B, WANG L. Evolutionary game dynamics of multi-agent cooperation driven by self-learning[C]// Proc. of the 9th Asian Control Conference, 2013.
[27] 王先甲, 夏可. 愿景驱动、演化博弈与环境污染治理进路[J]. 江汉论坛, 2018(7): 38－44.
WANG X J, XIA K. Aspiration driven, evolutionary game and environmental pollution control[J]. Jianghan Tribune, 2018(7): 38－44.
[28] DU J M, WU B, WANG L. Aspiration dynamics and the sustainability of resources in the public goods dilemma[J]. Physica A: Statistical Mechanics & its Application, 2016, 380(16): 1432－1436.
[29] SMITH E A. Communication and collective action: language and the evolution of human cooperation[J]. Evolution & Human Behavior, 2010, 31(4): 231－245.
[30] 禹明刚, 张东戈, 康凯, 等. 基于多元公共品演化博弈的无人集群合作演化机制[J]. 系统工程与电子技术, 2020, 42(12): 2787－2794.
YU M G, ZHANG D G, KANG K, et al. Cooperative evolution mechanism of unmanned swarm within the framework of public good game[J]. Systems Engineering and Electronics, 2020, 42(12): 2787－2794.
[31] Department of Defence. Unmanned systems integrated Roadmap FY2017-2042[R]. Office of the Secretary of Defense, 2013.
[32] 胡晓峰, 荣明. 智能化作战研究值得关注的几个问题[J]. 指挥与控制学报, 2018, 4(3): 195－200.
HU X F, RONG M. Several important questions of intelligent warfare research[J]. Journal of Command and Control, 2018, 4(3): 195－200.
[33] 李林林, 张承龙, 卓志敏. 智能无人作战系统发展及关键技术[J]. 现代防御技术, 2020, 48(3): 37－44.
LI L L, ZHANG C L, ZHUO Z M. Development and key technology of intelligent unmanned combat system[J]. Modern Defence Technology, 2020, 48(3): 37－43.
[34] 杨进, 沈文科. 对陆军智能化建设的深层思考[J]. 国防科技, 2019, 40(4): 41－44.
YANG J, SHEN W K. Deep thinking on army intelligence construction[J]. National Defense Science and technology, 2019, 40(4): 41－44.
[35] GARRO A, MAX M, TUNDIS A, et al. Intelligent agents: multi-agent systems[J]. Encyclopedia of Bioinformatics and Computational Biology, 2019. DOI: 10.1016/B978－0－12－809633－8.20328－2.
[36] 约翰·霍兰. 隐秩序—适应性造就复杂性[D]. 周晓牧译. 上海: 上海科技教育出版社, 2000.
HOLLAND J. Hidden order—adaptability makes complexity[D]. ZHOU X M. Shanghai: Shanghai Science and technology education press, 2000.
[37] YANG Z S, WANG W, KIM D. On multi-path routing for reliable communications in failure interdependent complex networks[J]. Journal of Combinatorial Optimization, 2021, 41(1): 170－196.
[38] CHONG Y Z, YANG H, LIU Z X, et al. Stability of replicator dynamics with bounded continuously distributed time delay[J]. Mathematics. 2020, 8(431): 1－11.
[39] GAO S P, WU T, WANG L. Evolution of global cooperation and ethnocentrism in group-structured populations[J]. Physics Letters A, 2018, 382(31): 2027－2043.
[40] GRAHAM R L, KNUTH D E, PATASHNIK O. Concrete mathematics [M]. 2nd ed. Massachusetts: Addison-Wesley Publishing Company, 1994.
[41] CHEN Y S, YANG H X, GUO W Z. Aspiration-induced dormancy promotes cooperation in the spatial Prisoner's Dilemma games[J]. Physica A: Statistical Mechanics and its Applications, 2017, 469: 625－630.
[42] PLATKOWSKI T. Aspiration-based full cooperation in finite systems of players[J]. Applied Mathematics and Computation, 2015, 251: 46－54.
[43] 王先甲, 刘佳. 具有外部性的合作博弈问题中的稳定的联盟结构[J]. 系统工程理论与实践, 2018, 38(5): 1173－1183.
WANG X J, LIU J. Stable coalition structures in cooperative game with externalities[J]. Systems Engineering-Theory & Practice, 2018, 38(5): 1173－1183.
[44] LIU X S, HE M F, KANG Y B, et al. Fixation of strategies with the Moran and Fermi processes in evolutionary games[J]. Physica A: Statistical Mechanics and its Applications, 2017, 484: 336－344.
[45] LIU X S, HE M F, KANG Y B, et al. Aspiration promotes cooperation in the prisoner's dilemma game with the imitation rule[J]. Physical Review E, 2016, 94: 012124.
[46] VAN K N G. Stochastic process in physics and chemistry[M]. Netherlands: Elsevier, 1992.
[47] GARDINER C W. Handbook of stochastic methods[M]. Berlin: springer, 1985.
[48] KIMURA M. The neutral theory of molecular evolution[M]. Cambridge: Cambridge University Press, 1983.
[49] TRAULSEN A, SEMMANN D, SOMMERFELD R D, et al. Human strategy updating in evolutionary games[J]// Proceedings of the National Academy of Science of the USA, 2010, 107(7): 2962－2966.
[50] WU B, ALTROCK P M, WANG L, et al. Universality of weak selection[J]. Physical Review E, 2010, 82: 046106.

作者简介

禹明刚(1986—), 男, 副教授, 博士后, 主要研究方向为军事需求工程、体系工程、集群智能。
何　明(1978—), 男, 教授, 博士研究生导师, 博士, 主要研究方向为军事信息学、智能化指挥控制。
张东戈(1965—), 男, 教授, 博士, 主要研究方向为复杂军事系统分析、军事信息学、军事运筹学。
马子玉(1995—), 男, 硕士研究生, 主要研究方向为无人化指挥控制。
康　凯(1986—), 男, 讲师, 硕士, 主要研究方向为军事需求工程、智能化指挥控制。

(C)1994-2022 China Academic Journal Electronic Publishing House. All rights reserved.　http://www.cnki.net
