# Supplementary material for: A game-based approach for designing a collaborative evolution mechanism for unmanned swarms on community networks
Source: Sci Rep. 2022 Nov 7;12:18892. doi: 10.1038/s41598-022-22365-z (PMC9640601; doi:10.1038/s41598-022-22365-z)
Supplement: Supplementary file 1 — Supplementary Information. [file 41598_2022_22365_MOESM1_ESM.zip › Supporting material/5.í╢A Mechanism for Identifying and Suppressing the Emergent Flocking Behaviors of UAV Swarmsú¿╬▐╚╦╗·í░╖Σ╚║í▒╡─╖Σ╙╡╙┐╧╓╨╨╬¬╩╢▒≡╙δ╥╓╓╞╗·└φú⌐í╖.pdf]

第2期 电 子 学 报 Vol. 47 No. 2
2019年2月 ACTA ELECTRONICA SINICA Feb. 2019

无人机“蜂群”的蜂拥涌现行为识别与抑制机理

柳 强[1,2],何 明[1,3],刘锦涛[1],牛彦杰[1],黄 倩[4]

(1. 陆军工程大学指挥控制工程学院,江苏南京 210007; 2. 海军指挥学院作战实验室,江苏南京 210000;
3. 军事科学院系统工程研究院网络信息研究所,北京,100071;4. 河海大学计算机与信息学院,江苏南京,211100)

摘 要: 为了抵消无人机“蜂群”所具有的非对称作战优势,从反控制其协同飞行的角度出发,将“蜂群”描述为具有涌现性特征的复杂系统,剖析无人机“蜂群”蜂拥涌现行为的产生机理,首次建立基于f-散度的“蜂群”涌现性度量模型. 根据蜂拥控制算法所遵循的启发式规则提出“蜂群”蜂拥涌现行为的抑制机理,首次建立了干扰条件下蜂拥控制的失效判别模型. 通过仿真实验,分析和讨论了干扰强度、干扰时机对抑制“蜂群”蜂拥涌现行为的影响.

关键词: 无人机“蜂群”; 涌现行为; 蜂拥控制; f-散度

中图分类号: V279 **文献标识码:** A **文章编号:** 0372-2112(2019)02-0374-08

电子学报 URL: http://www.ejournal.org.cn **DOI:** 10.3969/j.issn.0372-2112.2019.02.017

A Mechanism for Identifying and Suppressing the Emergent Flocking Behaviors of UAV Swarms

LIU Qiang[1,2],HE Ming[1,3],LIU Jin-tao[1],NIU Yan-jie[1],HUANG Qian[4]

(1. *College of Command and Control Engineering*,*Army Engineering University of PLA*,*Jiangsu*,*Nanjing* 210007,*China*;
2. *Department of Combat laboratory*,*Naval Command College*,*Jiangsu*,*Nanjing* 210000,*China*;
3. *Institute of Network Information*,*Academy of Systems Engineering*,*Academy of Military Sciences*,*Beijing* 100071,*China*;
4. *College of Computer and Information*,*Hohai University*,*Jiangsu*,*Nanjing* 211100,*China*)

Abstract: In order to neutralize the advantage of asymmetric operations owned by UAV swarms,UAV swarms were investigated from the perspective of negativing the coordinated flight control for them. Firstly,UAV swarms are described as a complex system with the emergence. Secondly,based the mechanism for flocking behaviors generated from UAV swarms, f-divergence based quantitative model was established to measure the emergence of them. It was firstly proposed that the suppression principle of swarms' emergent behaviors in term of three heuristic rules followed by general flocking control algorithms. Moreover,it was firstly constructed that the failure judgment model of flocking control for swarms under man-made interference. Finally,simulation experiments showed that the interference intensity and opportunity played an effect on suppressing emergent behaviors of swarms.

Key words: UAV swarms; emergent behavior; flocking control; f-divergence

1 引言

无人机“蜂群”是受自然界群居动物生活习性启发而设计的一类集群机器人(Swarm Robotics)[1]. 它没有集中式控制结构,通过无人机之间的局部交互以及无人机与外部环境的相互作用,涌现出设计者所期望的群体行为,如鸟群的蜂拥聚集. 作为一门新型技术,无人机“蜂群”无疑是一把“利刃”. 攻击方采用价格低廉的无人平台谋取作战成本的非对称效益,凭借多个协同作战关键技术[2,3](如自主编队飞行、协同态势感知、集群突防与)对敌方目标实施分布式探测与杀伤. 同时,在数量上呈现压倒性优势的无人机“蜂群”拥有灵活性好、鲁棒性强的分布式作战网络,可使敌方探测系统和武器系统瞬间饱和. 目前,防御方尚无有效的防御对抗手段. 为此,本文对无人机“蜂群”反制理论与技术进行了初步探索,从反控制无人机“蜂群”协同飞行的角度

收稿日期: 2018-04-02; **修回日期:** 2018-06-13; **责任编辑:** 蓝红杰
基金项目: 江苏省自然科学基金(No. BK20150721,No. BK20161469);中国博士后基金(No. 2015M582786,No. 2016T91017);江苏省重点研发计划(No. BE2015728,No. BE2016904,No. BE2017616);江苏省科技基础设施建设计划(No. BM2014391);国家重点研发计划(No. 2016YFC0800606,No. 2016YFC0800310);中央高校基本科研业务费项(No. 2017B42214)

(C)1994-2022 China Academic Journal Electronic Publishing House. All rights reserved. http://www.cnki.net

出发，将无人机“蜂群”抽象成具有涌现性特征的复杂系统，分析无人机“蜂群”蜂拥涌现行为的产生机理，结合涌现行为源自个体的局部交互的本质，并提出相应的抑制机理.

此外，本文在蜂拥涌现行为识别与蜂拥涌现行为抑制两方面做出了创新性工作. 在蜂拥涌现行为识别方面，学术界目前尚未有统一的方法，部分方法在具体应用中也有一定的局限性，如识别前需要大量带有标记的训练样本[4,5]，度量指标变量需要系统内部的网络拓扑结构参数[6,7]. 为此，本文从防御方反制无人机“蜂群”的角度，站在复杂系统外部对涌现行为进行观察，凭借己方传感器获取的无标签观测数据，首次提出基于 f-散度的无人机“蜂群”涌现性度量方法. 在蜂拥涌现行为抑制方面，在事先无法获知无人机“蜂群”内部网络拓扑结构的前提下，从破坏“蜂群”内部通信网络的角度出发首次建立了干扰条件下无人机“蜂群”蜂拥控制的失效判别模型，为动态反映无人机“蜂群”群体飞行反控制效果提供了技术手段.

2　蜂拥涌现行为产生机理

如图 1 所示，“蜂群”的蜂拥行为是在个体之间内部经过多次迭代交互形成的，彼此之间交换飞行状态控制信息，如位置、速度、航向等. 同时，每个个体都采用相同的行为规则[8]：(1) 分离，与邻域内其它无人机避免发生碰撞；(2) 聚合，与邻域内其它无人机保持紧凑；(3) 匹配，与邻域内其它无人机运动速度基本保持一致. 在微观层面，各个“蜂群”无人机都基于自身的行为规则行事；而在宏观层面，整个“蜂群”在某个空间聚集的系统状态会直接反馈给微观层中的无人机.

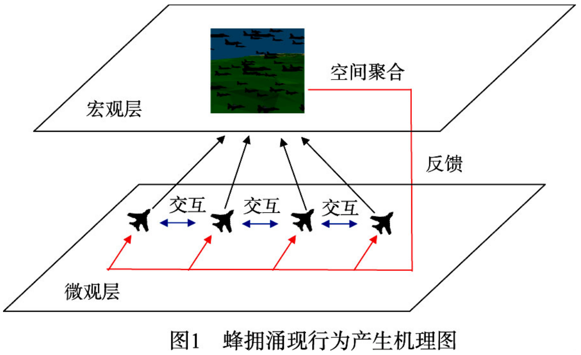

图1　蜂拥涌现行为产生机理图

3　蜂拥涌现行为识别

根据蜂拥涌现行为的产生机理，涌现行为并不可约分为微观层中的个体行为，在识别复杂系统的涌现现象之前，需要站在宏观的视角对复杂系统进行整体观察. 作为无人机“蜂群”的防御方，更多的是借助己方建立的分布式传感器网络对其进行探测、识别与跟踪，获取各“蜂群”无人机的位置、速度、航向等信息，需要将这些信息作为从宏观上理解和分析涌现行为的原始数据，计算系统在不同时刻的涌现程度，即涌现性度量，通过设定涌现行为识别门限，获知系统是否正处在产生涌现现象的阶段. 在该阶段，“蜂群”内部的无人机之间会进行局部交互(通信)，这为防御方积极采取反制措施建立了快速响应的时间窗口. 为此，蜂拥涌现行为识别是蜂拥涌现行为抑制的先决条件.

在概率论中，f-散度常作为衡量两个概率分布异同程度的指标函数. 对于无人机“蜂群”，f-散度同样可以用来比较不同时刻“蜂群”群体空间分布的差异，根据差异程度对蜂拥涌现行为进行宏观分析.

3.1　f-散度

如式(1)所示，给出了等人[8]提出的 f-散度度量公式.

$$D_f(P(x) \| Q(x)) = \int_\Omega f\left(\frac{p(x)}{q(x)}\right) q(x)\, \mathrm{d}\mu \qquad (1)$$

在工程应用中，常采用离散形式的 f-散度进行度量，即

$$D_f(P(x) \| Q(x)) = \sum_{i=1}^{N} q_i f\left(\frac{p_i}{q_i}\right) \qquad (2)$$

其中，p_i 和 q_i 分别为概率分布集合 $P=\{p_1,p_2,\cdots,p_N\}$ 和 $Q=\{q_1,q_2,\cdots,q_N\}$ 在对应概率空间 Ω 上的概率值，并满足

$$\begin{cases} \sum_{i=1}^{N} p_i = 1, & p_i \ge 0 \\ \sum_{i=1}^{N} q_i = 1, & q_i \ge 0 \end{cases} \qquad (3)$$

在常用的 5 种散度度量函数[10]中，Hellinger Distance 形式的度量函数具有收敛性好和灵敏度高的特点，故选用它作为 f-散度的可度量函数，即 Hel 散度，其函数形式如式(4)所示：

$$D_f(P(x) \| Q(x)) = 1 - \sum_{i=1}^{N} \sqrt{p_i q_i} \qquad (4)$$

3.2　三维核密度估计

对于无人机“蜂群”，采用 f-散度度量其涌现性的关键是估计其在三维观测样本空间内特定时刻的概率密度 $\hat{p}(x)$ 和 $\hat{q}(x)$，并将其转换为离散形式下对应的概率 $\hat{p}_i$ 和 $\hat{q}_i$. 在不利用观测样本数据先验信息，不对观测样本数据分布作任何假定的情况下，我们可以采用非参数估计的多元核密度估计(multi-variate kernel density estimation，MV-KDE)方法[11]估计 $\hat{p}(x)$ 和 $\hat{q}(x)$，其具体形式如式(5)所示：

$$\hat{f}_h(\boldsymbol{x}) = \frac{1}{N}\sum_{i=1}^{N} K\left(\frac{\boldsymbol{x}-\boldsymbol{X}_i}{h}\right) \qquad (5)$$

其中，$\hat{f}_h(\boldsymbol{x})$ 为所要估计的概率密度函数，$\boldsymbol{X}_1,\boldsymbol{X}_2,\cdots,\boldsymbol{X}_i,\cdots,\boldsymbol{X}_N$ 为 d 维随机变量样本集(即“蜂群”无人机的三维

(C)1994-2022 China Academic Journal Electronic Publishing House. All rights reserved.　　http://www.cnki.net

空间位置)，h 为平滑系数，N 为样本数量(即“蜂群”无人机的总数量)，$K(\cdot)$ 为核函数，采用三个一维核函数连乘的形式，即 $K(\frac{x-X_i}{h})=\prod_{k=1}^{3}\frac{1}{\sqrt{2\pi}h}\exp(-\frac{(x-X_{ik})^2}{2h^2})$.

如图 2 所示，将三维观测样本空间作离散化处理，并假定离散化后的 cell 区域内概率密度值为常值，再根据实际需要将观测空间划分为多个大小相同的观测子区域，由此换算成三维观测样本空间的联合概率分布，如 $P=\{p_1,p_2,\cdots,p_N\}$.

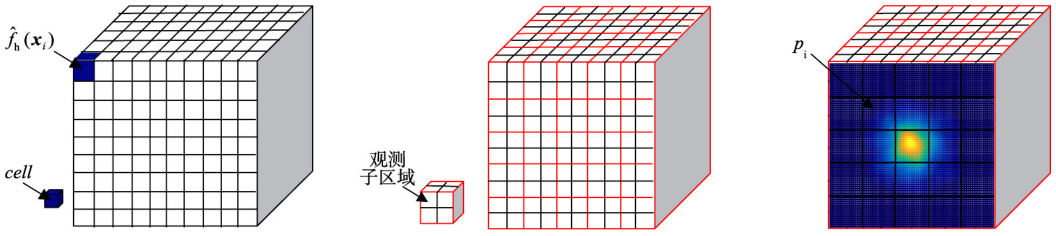

(a) 离散 (b) 分区 (c) 计算

图2 联合概率分布换算示意图

4 有序性度量

哈肯[12]曾在其创立的协同学理论中给出了关于有序性的定义：一个由大量子系统所构成的系统，通过子系统间的非线性相互作用与协作，形成了具有一定功能的自组织结构，该结构在宏观上呈现为有序的时间结构、空间结构或时空结构，则系统达到了新的有序状态. 对于无人机“蜂群”，为了保持在空间飞行的群聚形态，需要无人机个体与友邻无人机发生局部交互作用，在宏观上完成由无序时空结构到有序时空结构的过渡、转变，这说明无人机“蜂群”除了具有涌现性，还具有哈肯所说的有序性. 为此，我们采用序参量来反映无人机“蜂群”蜂拥涌现行为受到抑制前后的有序程度变化，以辅助分析防御方抑制敌方“蜂群”蜂拥涌现行为的效能. 如式(6)(7)所示，我们分别采用航向一致性和平均相对距离两个序参量[13,14]来度量“蜂群”群体飞行的有序性.

$$\varphi=\frac{1}{N}\left\|\sum_{i=1}^{N}\frac{\boldsymbol{v}_i}{\|\boldsymbol{v}_i\|}\right\| \tag{6}$$

$$d_{avg}=\frac{1}{N}\sum_{i=1}^{N}\sum_{j=1}^{N}\|\boldsymbol{X}_i-\boldsymbol{X}_j\| \tag{7}$$

其中，$\boldsymbol{v}_i$ 为第 i 架“蜂群”无人机的速度矢量，其它符号含义同前.

5 蜂拥涌现行为抑制机理

5.1 抑制机理

屈强[10,15]曾指出，环境刺激是涌现的外因，而个体之间的相互作用是涌现的内因. 而无人机“蜂群”正是借助内部通信网络与邻域内个体进行局部交互，实现刺激信息在“蜂群”整个网络内部的层层有效传播，对“蜂群”通信网络的干扰必然会对蜂拥涌现行为产生抑制.

如图 3 所示，当某节点的通信接收机受到干扰后，该节点将无法接收友邻节点的运动状态信息，失去了与友邻节点协调一致行动的机会，如无法实现聚合、避碰或速度一致等协同动作. 当整个“蜂群”的通信网络受到压制时，“蜂群”内部无人机的飞行控制将出现紊乱，“蜂群”也将面临解体.

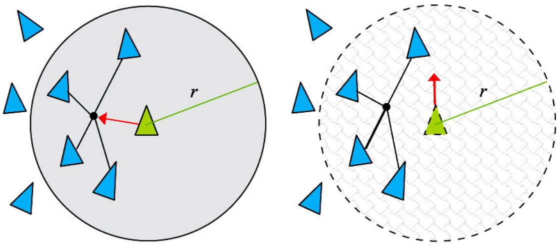

(a) 抑制前 (b) 抑制后

图3 “蜂群”蜂拥涌现行为抑制原理图

5.2 失效判别模型

目前，在多智能体蜂拥控制领域，以 Olfati-Saber 提出的算法[16]最为代表性，并在分布式移动传感器网络研究方向衍生出一系列具有实际工程意义的多目标蜂拥跟踪控制算法. 本文在该算法框架基础上，首次提出了干扰条件下无人机“蜂群”蜂拥控制失效判别模型，以动态反映无人机“蜂群”群体飞行控制失效后的效果.

为后续描述模型方便，给出如下定义.

定义 1：将无人机“蜂群”抽象成一个复杂网络，无人机 i 视为网络中的一个节点，节点之间的通信关系用直线 E_{ij} 表示，由此“蜂群”内全部无人机的集合记为 $V=\{V_1,V_2,\cdots,V_n\}$，网络中边的集合记为 $E=\{E_{ij}=(V_i,V_j):V_i,V_j\in V\}$，遭受通信干扰的节点记为 J，且 $J\subseteq V$，节点 i 的邻近节点集合记为 $N_i=\{V_j\in V:(i,j)\in E\}$.

无人机“蜂群”的蜂拥行为通过 3 个控制项间接实现，即 $\boldsymbol{u}'_i=\boldsymbol{u}_i^g+\boldsymbol{u}_i^d+\boldsymbol{u}_i^\gamma$. 其中，$\boldsymbol{u}_i^g$ 表示分离—聚合控制项，实现无人机之间的分离与聚合，$\boldsymbol{u}_i^d$ 表示速度匹配

(C)1994-2022 China Academic Journal Electronic Publishing House. All rights reserved. http://www.cnki.net

项，实现无人机之间的速度一致，u_i^{γ} 表示反馈引导项，引导无人机飞向目标. 当部分“蜂群”无人机节点通信设备受到干扰时，将无法与“蜂群”内其它无人机彼此交换飞行控制信息. 由此，进一步得到干扰条件下的蜂拥控制算法，如式(8)所示.

$$u'_i = \underbrace{\sum_{j\in N_i/J}\varphi_\alpha(\|X_j - X_i\|_\sigma)\frac{X_j - X_i}{\sqrt{1+\varepsilon\|X_j - X_i\|^2}}}_{u_i^g} + \underbrace{\sum_{j\in N_i/J}a_{ij}(X)(v_j - v_i)}_{u_i^d} + u_i^{\gamma},\quad i\in V/J \tag{8}$$

式(8)中的 u'_i 表示第 i 个“蜂群”无人机在未受到通信干扰情况下的蜂拥控制向量，它只能与未受通信干扰的节点交换位置和速度信息，式(8)的其它各符号含义详见文献[16].

由于无人机通信是双向的，如果干扰条件下与某些无人机交换飞行控制信息的节点减少时，则在友邻区域内与其实现协调一致运动的节点也在相应地减少，从而造成“蜂群”在某个空域内部分无人机节点发生局部失调.

6　仿真与分析

6.1　仿真设置

为动态展现无人机“蜂群”蜂拥涌现行为的识别与抑制过程，我们构建了无人机“蜂群”攻防对抗原型系统，设置了如图4所示的想定场景: A 恐怖组织(红方)企图通过摧毁 B 国(蓝方)重要军事基础设施的方式达到扰乱 B 国安定团结、分裂国家的政治目的. 红方凭借多方情报信息准确获知蓝方重要军事基础设施的准确位置，在距蓝方目标 100km 处起飞数十架无人机，并在特定空域形成无人机“蜂群”，试图对蓝方目标实施饱和式攻击; 蓝方依托在己方保护目标附近部署的各类传感器对周边空域进行感知，一旦发现有不明意图的无人机，立即采取电子对抗措施予以反击.

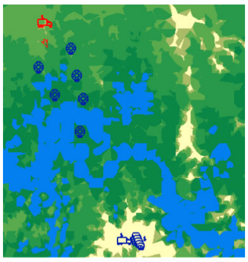

图4　反恐作战想定

初始仿真场景设置如图4所示，红方各无人机随机部署在(34°42′52″N，86°43′32″W)附近空域，飞行速度为 50m/s，飞行高度为 1000m，蓝方重要军事目标部署在(34°28′33″N，86°35′32″W)，蓝方通信干扰机部署在(34°28′29″N，86°36′51″W)，蓝方低空补盲雷达部署在多个前沿阵地，以便对周边空域进行监视. 其它相关仿真参数设置如下: $r = 36\text{m}$，$d = 30\text{m}$，$a = b = 5$，$\varepsilon = 0.1$，$h = 0.6$，$c_1 = 0.02$，$c_2 = 0.2$.

6.2　仿真分析

6.2.1　涌现性度量分析

在识别“蜂群”蜂拥涌现行为的仿真实验中，我们引入了基于信息熵[17,18]的涌现性度量方法，与本文提出的基于 f-散度的涌现性度量方法作比较，以验证各类方法的有效性和优缺点. 在基于信息熵的涌现性度量方法中，分别采用了 Mnif[17] 提出的离散熵差(discrete entropy difference，DED)以及程健[18] 提出的连续熵差(continuous entropy difference，CED). 两种方法的本质都是利用熵差来度量涌现性，区别在于: 前者用离散估计的方式(如直方图法)计算熵值，而后者在前者的基础上采用更为精确的连续估计方式(如 KDE)计算熵值. 两种计算方法的细节详见文献[17,18]，不再赘述. 需要注意的是，当所观测系统的涌现性度量变量是由多个系统属性构成时，DED 方法要求逐个对每一属性进行涌现性度量，如无人机“蜂群”在仿真场景中的 X、Y、Z 坐标，即图5中的 DED-X、DED-Y、DED-Z.

如图5、图6所示，无干扰条件下，在仿真时刻 $t = 10\text{s}$ 以后，Hel 散度值明显在增大，说明“蜂群”在局部交互作用下，其蜂拥涌现行为正在形成，并在三维空间中呈现类似鸟群“聚集”飞行的形态. 当“蜂群”持续飞行一段时间后，Hel 散度值将达到最大并趋于稳定，说明整个“蜂群”在不同时刻的空间位置分布差异基本保持不变，即“蜂群”已形成稳定有序的飞行队形. 通过图7不同仿真时刻所显示的“蜂群”三维空间位置分布，能够动态观察“蜂群”从无序到有序状态过渡的全过程，其结果与图5的仿真结果基本吻合.

由图5可知，基于信息熵的方法(即 DED 和 CED)并不能有效识别“蜂群”的蜂拥涌现行为. 根本原因在于基于信息熵和 f-散度的涌现性度量方法对涌现性的度量方式有所不同. 基于信息熵的涌现性度量方法倾向于从系统有序性角度来度量涌现性，但有序性并不完全等同于涌现性，学术界对此存在争议，只是认为复杂系统的有序性会伴随着涌现现象而出现. 值得注意的是，多属性系统的熵值度量与实际系统特性密切相关，不能仅仅通过简单的叠加方式(如加权平均)得到系统涌现程度的综合评价. 这也是 DED 方法的局限性. 而基于 f-散度的涌现性度量方法，它倾向于从统计分布的角度来度量涌现性，通过比较观测样本分布的差异来计算涌现程度，而且这种计算方式不会受到多属性问题的影响，因而用在度量无人机“蜂群”涌现性方面更为自然.

(C)1994-2022 China Academic Journal Electronic Publishing House. All rights reserved.　http://www.cnki.net

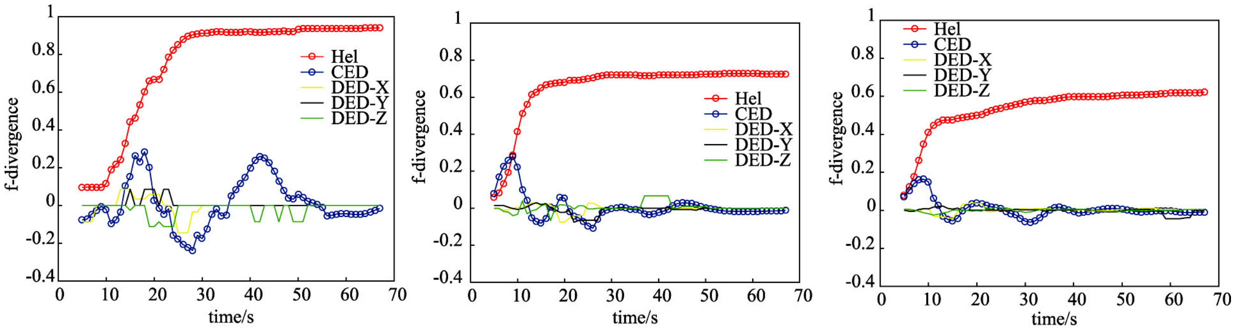

(*a*) *N*=10 (*b*) *N*=50 (*c*) *N*=100

图5 不同“蜂群”规模的涌现性度量曲线

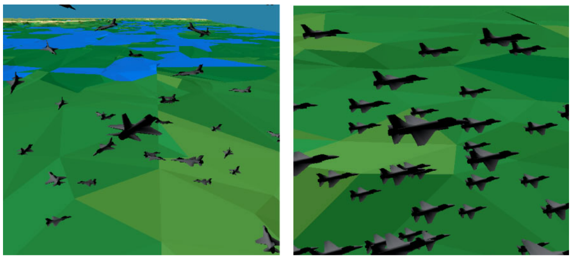
(*a*) *t*=6 s (*b*) *t*=25 s

图6 “蜂群”蜂拥涌现行为产生前后对照图

显然，基于 f-散度的涌现性度量方法较基于信息熵的方法具有一定优越性. 但在自动识别“蜂群”蜂拥涌现行为的实际过程中，仍然需要根据经验设定门限来判断“蜂群”蜂拥涌现行为正在产生的“升温点”，如 $e_{\mathrm{Hel}}=0.25$，以此作为反制“蜂群”的最佳时间窗口起始点.

6.2.2 干扰强度对蜂拥涌现行为的影响分析

在对“蜂群”通信网络实施干扰压制时，由于对抗双方的武器装备性能各不相同，造成实际的对抗效果也有所不同. 若要达到理想的“蜂群”通信压制效果，需要完成空间对准、频率对准和能量对准. 为简化复杂的建模工作，本文采用概率模型来模拟真实的通信对抗效果，如图 7、图 8 所示，将干扰压制概率分别为 50% 和 75%，分析“蜂群”的蜂拥涌现行为的抑制效果. 由于“蜂群”是高度冗余的自组织作战网络，部分未受通信干扰的无人机仍然能彼此进行交互，并在三维空间中实现聚集，如图 7(*a*)、图 8(*a*)所示的黑色圆圈部分；而另一部分“蜂群”无人机因长时间受到通信干扰而无法执行任务，则返回任务起始航路点，成为孤立的作战单元，如图 7(*a*)、图 8(*a*)所示的黑色方框部分. 同时，采用第 4 节度量有序性的两个指标辅助分析“蜂群”蜂拥涌现行为的抑制效果，如图 7(*b*)、图 7(*c*)和图 8(*b*)、图 8(*c*)所示. 由仿真结果可知，未受到通信干扰的无人机所组成的“蜂群”，其航向一致性和平均相对距离均保持不变，说明这些无人机在动态的网络拓扑条件下仍能形成新的稳定“蜂群”；而受到通信干扰的无人机所组成的“蜂群”，其航向一致性的度量值较低，而平均相对距离则逐渐增大，说明这些无人机由原来的有序状态向无序状态过渡.

6.2.3 干扰时机对蜂拥涌现行为的影响分析

事实上，“蜂群”无人机在投放阶段，“蜂群”尚未成型，绝大多数“蜂群”无人机始终处于无序的混乱状态，若此时防御方能够发现该情况，则可占领先机，率先对其发起电子进攻，将“蜂群”扼杀在“摇篮”中. 针对上述情况，我们将通信干扰机的干扰起始时间设为 $t_{jam}=5\mathrm{s}$，得到如图 9 所示的仿真结果. 由图 9(*a*)可知，φ 值始终保持在 0.13 附近，d_{avg} 值则越来越大，说明“蜂群”始终处于无序的混乱状态. 需要注意的是，虽然这种方式的

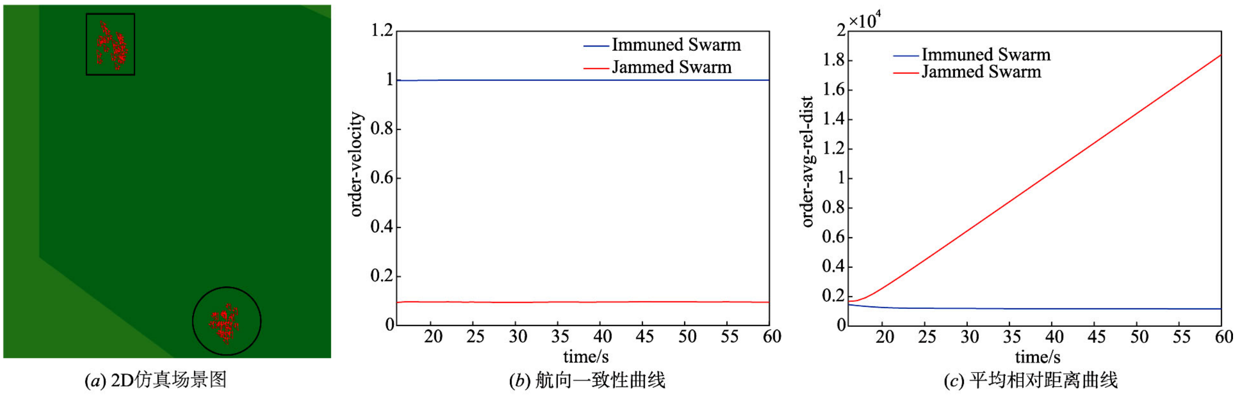

(*a*) 2D仿真场景图 (*b*) 航向一致性曲线 (*c*) 平均相对距离曲线

图7 “蜂群”蜂拥涌现行为抑制效果图（$P_{压制}=0.5$）

(C)1994-2022 China Academic Journal Electronic Publishing House. All rights reserved. http://www.cnki.net

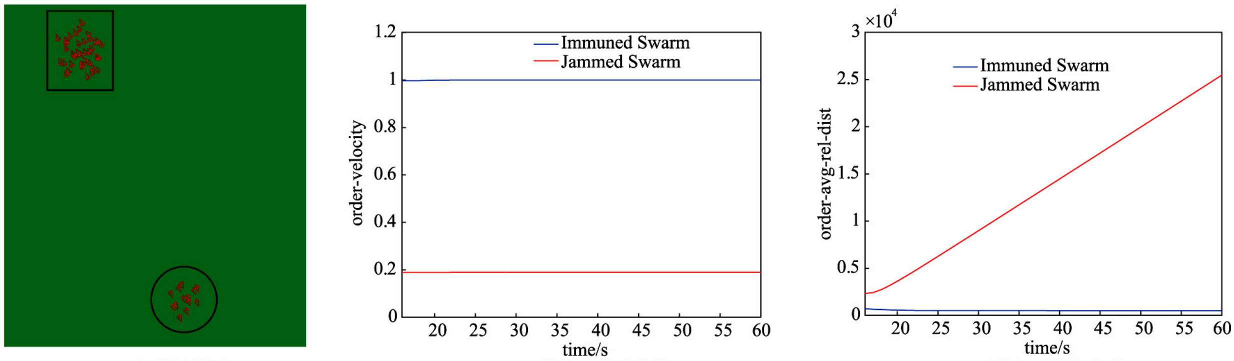

(*a*) 2D仿真场景图　　(*b*) 航向一致性曲线　　(*c*) 平均相对距离曲线

图8 “蜂群”蜂拥涌现行为抑制效果图（$P_{压制}$=0.75）

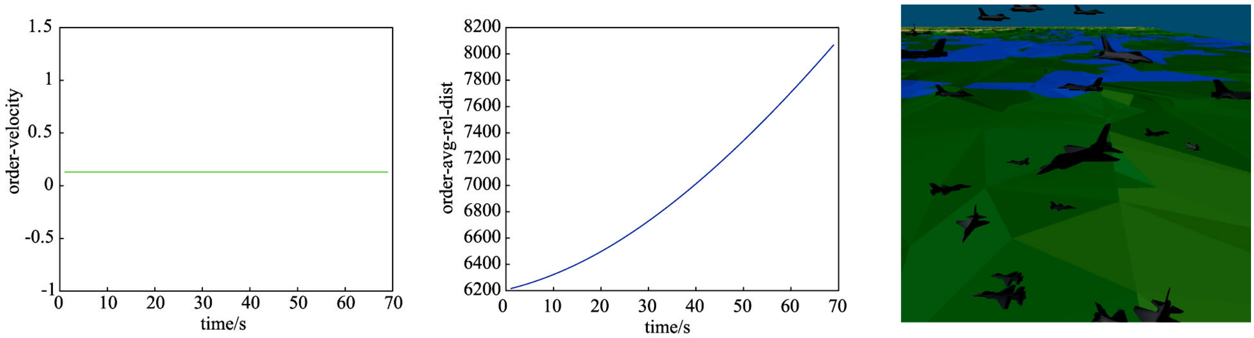

(*a*) 航向一致性曲线　　(*b*) 平均相对距离曲线　　(*c*) 3D仿真场景图

图9　早期压制干扰对蜂拥涌现行为的影响

干扰效果更好，但“蜂群”无人机在三维空间上分散程度更高，若要达到理想的反制效果需要调度更多的干扰资源对其进行压制，与识别“蜂群”蜂拥涌现行为后再采取通信干扰的策略相比，后者的反制效果会更好.

若防御方采取反制措施的时机比较晚，同时“蜂群”已经形成比较稳定的飞行队形，如 $t_{jam}=55$s. 我们假设无人机受到通信干扰后仍然飞向任务区，其情况会有何不同? 从原理上，“蜂群”达到稳态后，各“蜂群”无人机的航向、速度、相对位置基本趋于一致，此时防御方对其实施电子压制来抑制蜂拥涌现行为是徒劳的. 由如图 10(*a*)(*b*) 可知，φ 值和 d_{avg} 值在 $t=55$s 后始终保持不变，说明“蜂群”的飞行队形并没有被打乱. 但从信息融合的角度来说，此时，无人机不能建立信息共享机制，导致其各自为战，无法对重要目标实施饱和式攻击，这种干扰策略在一定程度上也是有意义的.

综上，在面临数量众多的无人机“蜂群”攻击时，运用适当的干扰策略是必要的，过早或过晚采取反制措施都不是理想的对抗方案，而基于 f-散度的涌现性度量方法恰能利用传感器网络观测到的“蜂群”运动状态信息，从宏观上识别其蜂拥涌现行为，为建立快速的“蜂群”应对响应机制提供了先决条件，打开了应对处置的时间窗口. 同时，考虑到无人机“蜂群”是高度冗余的自

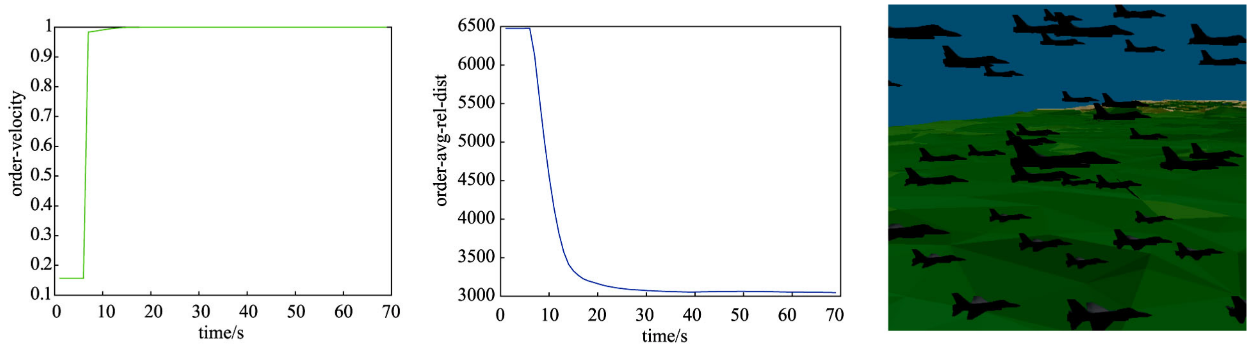

(*a*) 航向一致性曲线　　(*b*) 平均相对距离曲线　　(*c*) 3D仿真场景图

图10　滞后压制干扰对蜂拥涌现行为的影响

(C)1994-2022 China Academic Journal Electronic Publishing House. All rights reserved.　http://www.cnki.net

组织作战网络，单点故障所引起的破坏并不会让系统的功能瞬间消失，即无人机“蜂群”的涌现现象不会立即消失．为此，应尽可能地利用更多的电子干扰资源对其进行通信压制，将“蜂群”内的无人机孤立成独立的作战个体，在一定程度上削弱“蜂群”所体现的整体大于部分之和的作战优势．

7 结束语

本文在剖析无人机“蜂群”蜂拥涌现行为产生机理的基础上，首次运用f-散度度量无人机“蜂群”的涌现性，以此来识别蜂拥涌现行为．根据蜂拥控制算法背后所采用的启发式规则提出“蜂群”蜂拥涌现行为的抑制原理，首次建立了干扰条件下无人机“蜂群”蜂拥控制的失效模型．通过仿真实验，分析和讨论了干扰强度、干扰时机对抑制“蜂群”蜂拥涌现行为的影响，并指出“在正确识别无人机“蜂群”涌现行为的前提下对其进行通信压制是抑制其涌现行为产生的最佳反制策略”，这为后续开展无人机“蜂群”反制理论与技术奠定了基础．

本文提出了一种基于完全信息的涌现性度量方法，即假设防御方完全掌握无人机“蜂群”在不同时刻的运动状态信息．在未来的工作中，我们将进一步讨论在不完全信息情况下的涌现性度量方法，以贴近实战．

参考文献

[1] Ahin E. Swarm robotics: from sources of inspiration to domains of application [A]. Sahin E, Spears W M. International Conference on Swarm Robotics [C]. Berlin, Heidelberg: Springer, 2004. 10 - 20.

[2] 钮伟，黄佳沁，缪礼锋．无人机蜂群对海作战概念与关键技术研究 [J]．指挥控制与仿真，2018，40(1)：20 - 27.
Niu Wei, Huang Jiaqin, Miao Lifeng. Research on the concept and key technologies of unmanned aerial vehicle swarm concerning naval attack [J]. Command Control & Simulation, 2018, 40(1): 20 - 27. (in Chinese)

[3] 高杨，李东生．无人机集群协同态势觉察一致性评估 [J]．电子学报，2019，47(1)：190 - 196.
Gao Yang, Li Dongsheng. UAV swarm cooperative situation perception consensus evaluation [J]. Acta Electronica Sinica, 2019, 47(1): 190 - 196. (in Chinese)

[4] Wagner G, Choset H. Gaussian reconstruction of swarm behavior from partial data [A]. Seattle, WA, USA: IEEE International Conference on Robotics and Automation [C]. IEEE, 2015. 5864 - 5870.

[5] Berger M, Seversky L M, Brown D S. Classifying swarm behavior via compressive subspace learning [A]. Stockholm, Sweden: IEEE International Conference on Robotics and Automation [C]. IEEE, 2016. 5328 - 5335.

[6] Birdsey L, Szabo C. An architecture for identifying emergent behavior in multi-agent systems [A]. International Conference on Autonomous Agents and Multi-Agent Systems. International Foundation for Autonomous Agents and Multiagent Systems [C]. Paris, France: ACM, 2014. 1455 - 1456.

[7] Szabo C, Teo Y M. Formalization of weak emergence in multiagent systems [J]. ACM Transactions on Modeling & Computer Simulation, 2015, 26(1): 6.

[8] Reynolds C W. Flocks, herds and schools: A distributed behavioral model [J]. ACM SIGGRAPH Computer Graphics, 1987, 21(4): 25 - 34.

[9] Ali S M, Silvey S D. A general class of coefficients of divergence of one distribution from another [J]. Journal of the Royal Statistical Society. Series B (Methodological), 1966, 131 - 142.

[10] 屈强，何新华，陆皖麟．基于f-散度的复杂系统涌现度量方法 [J]．装甲兵工程学院学报，2017，31(3)：106 - 110.
Qu Qiang, He Xinhua, Lu Wanlin. A new approach to measure the emergence of complex system based on f-divergence [J]. Journal of Academy of Armored Force Engineering, 2017, 31(3): 106 - 110. (in Chinese)

[11] Botev Z I, Grotowski J F, Kroese D P. Kernel density estimation via diffusion [J]. The Annals of Statistics, 2010, 38(5): 2916 - 2957.

[12] 赫尔曼·哈肯．协同学：大自然构成的奥秘 [M]．上海：上海译文出版社，2001，7 - 9.

[13] Vicsek T, Czirók A, Ben-Jacob E, et al. Novel type of phase transition in a system of self-driven particles [J]. Physical review letters, 1995, 75(6): 1226.

[14] Liu M, Yang P, Lei X, et al. Self-organized fission control for flocking system [J]. Journal of Robotics, 2015, 2015.

[15] 屈强，何新华，刘中晅．系统涌现的要素和动力学机制 [J]．系统科学学报，2017，25(3)：25 - 29.
Qu Qiang, He Xinhua, Liu Zhongxuan. Essential factors and dynamic mechanism of the system emergence [J]. Journal of Systems Science, 2017, 25(3): 25 - 29. (in Chinese)

[16] Olfati-Saber R. Flocking for multi-agent dynamic systems: algorithms and theory [J]. IEEE Transactions on Automatic Control, 2006, 51(3): 401 - 420.

[17] Mnif M, Müller-Schloer C. Organic computing—a paradigm shift for complex systems [J]. Springer, Basel, 2011, 27(4): 332 - 336.

[18] 程建，张明清，唐俊，等．基于信息熵的复杂系统涌现量化方法研究 [J]．信息工程大学学报，2014，15(3)：270 - 274.

(C)1994-2022 China Academic Journal Electronic Publishing House. All rights reserved. http://www.cnki.net

Cheng Jian，Zhang Mingqing，Tang Jun，et al. Emergence quantitative analysis of complex adaptive systems based on shannon's information entropy [J]. Journal ofInformation Engineering University，2014，15(3)：270 – 274. (in Chinese)

作者简介

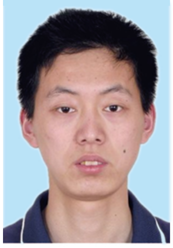

柳　强　男，1983 年出生于辽宁锦州. 讲师，博士研究生，研究方向为指挥控制系统工程.
E-mail：lq_paper_2018@163. com

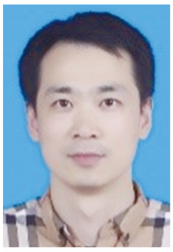

何　明(通信作者)　男，1978 年出生于新疆石河子，教授，博士生导师，研究方向为指挥控制系统工程、人工智能.
E-mail：hm_paper@sina. com

刘锦涛　男，1981 年出生于山东烟台. 工程师，博士，研究方向为自动控制工程.
E-mail：top1944@163. com

牛彦杰　女，1979 年出生于安徽界首. 讲师，硕士，研究方向为军事运筹学.
E-mail：niuyanjie@126. com

黄　倩　男，1981 年出生于江苏海安. 副研究员，博士，研究方向为多媒体计算、大数据分析.
E-mail：huangqian@hhu. edu. cn

(C)1994-2022 China Academic Journal Electronic Publishing House. All rights reserved.　http://www.cnki.net
