# Supplementary material for: A game-based approach for designing a collaborative evolution mechanism for unmanned swarms on community networks
Source: Sci Rep. 2022 Nov 7;12:18892. doi: 10.1038/s41598-022-22365-z (PMC9640601; doi:10.1038/s41598-022-22365-z)
Supplement: Supplementary file 1 — Supplementary Information. [file 41598_2022_22365_MOESM1_ESM.zip › Supporting material/6.í╢Research on unmanned aerial vehicle swarms system resilienceú¿╬▐╚╦╗·╝»╚║╧╡═│╡»╨╘╤╨╛┐ú⌐í╖.pdf]

第 43 卷 第 1 期
2021 年 1 月

系统工程与电子技术
Systems Engineering and Electronics

Vol. 43 No. 1
January 2021

文章编号:1001-506X(2021)01-0156-07

网址:www.sys-ele.com

无人机集群系统弹性研究

顾凌枫[1], 何 明[1], 陈国友[1], 季敏惠[2], 刘锦涛[1]
(1. 陆军工程大学指挥控制工程学院, 江苏 南京 210001; 2. 江苏省公安厅, 江苏 南京 210001)

摘 要:为提升无人机集群(unmanned aerial vehicle swarm, UAVS)应对恶劣天气、人为干扰、组件故障等扰动的能力,借鉴复杂网络弹性理念,对 UAVS 扰动场景进行建模,提出了 UAVS 弹性的概念与内涵,通过与鲁棒性、可靠性、抗毁性等指标对比,阐述了弹性指标对于研究 UAVS 系统特性的意义;分析 UAVS 性能变化曲线和选取指标,刻画了弹性定量指标,从而提出了 UAVS 系统弹性评估方法;从吸收扰动和恢复两方面,提出了 UAVS 系统弹性的优化策略。最后,展望 UAVS 系统弹性未来研究的发展趋势。

关键词: 无人机集群; 复杂网络; 弹性; 扰动
中图分类号: TP 273 **文献标志码**: A **DOI**:10.3969/j.issn.1001-506X.2021.01.19

Research on unmanned aerial vehicle swarm system resilience

GU Lingfeng[1], HE Ming[1], CHEN Guoyou[1], JI Minhui[2], LIU Jintao[1]
(*1. School of Command and Control Engineering, Army Engineering University, Nanjing 210001, China;*
2. Department of Public Security of Jiangsu Province, Nanjing 210001, China)

Abstract: In order to improve the ability of the unmanned aerial vehicle swarm (UAVS) to deal with disturbances such as severe weather, electronic jamming, component failure, etc., the UAVS disturbance scenarios are modeled, the concept and connotation of the UAVS resilience is proposed by referring to the concept of complex network resilience. By comparing with the robustness, reliability, invulnerability and other indicators, the significance of resilience for studying the characteristics of UAVS is explained. The performance curve and selected indicators of UAVS are analyzed, the quantitative indicator of resilience is depicted, and the methods for evaluating the resilience of UAVS system are proposed. The optimization strategy for UAVS system resilience is put forward from two spects of disturbance absorption and recovery. Finally, the development tendency of future research on the resilience of UAVS system is forecasted.

Keywords: unmanned aerial vehicle swarm(UAVS); complex network; resilience; disturbance

0 引 言

无人机集群(unmanned aerial vehicle swarm, UAVS)是由一定数量的无人机(unmanned aerial vehicle, UAV)组成,以交感网络为基础,整体具有自组织特性的空中移动多智能体系统[1]。在侦察、监视、搜救、作战等方面,UAVS 具有广阔的应用前景[2],引起了广泛关注[3]。

UAVS 领域现有研究主要集中于改进任务执行性能,如集群拓扑设计[4]、协同控制[5-6]、路径规划[7-8]、任务规划[9-11]等。上述研究普遍默认 UAVS 处于无干扰环境,但在现实情况中,UAVS 运行环境中存在着大量扰动,这些扰动可以分为外部因素和内部因素。外部因素包括恶劣天气影响、人为干扰破坏[12]等;内部因素包括组件故障、通信中断等。扰动会造成 UAVS 网络节点失效、链路中断等问题,进而导致集群网络拓扑发生变动,影响 UAVS 任务执行性能。扰动具有不可预测性,包括扰动来源、

收稿日期:2020-02-22; 修回日期:2020-06-30; 网络优先出版日期:2020-08-20。
网络优先出版地址:https://kns.cnki.net/kcms/detail/11.2422.TN.20200820.0910.004.html
基金项目:国家重点研发计划(2018YFC0806900);中国博士后科学基金(2018M633757);江苏省重点研发计划(BE2016904、BE2017616,BE2018754,BE2019762)资助课题
引用格式: 顾凌枫, 何明, 陈国友, 等. 无人机集群系统弹性研究[J]. 系统工程与电子技术, 2021, 43(1):156-162.
Reference format: GU L F, HE M, CHEN G Y, et al. Research on unmanned aerial vehicle swarm system resilience[J]. Systems Engineering and Electronics, 2021, 43(1):156-162.

(C)1994-2022 China Academic Journal Electronic Publishing House. All rights reserved. http://www.cnki.net

扰动范围以及扰动造成后果的随机性和不可预测性；扰动同时具有不可避免性，即对于 UAVS 要做好必然遭受扰动的准备。因此，对 UAVS 承受扰动能力进行考察，是十分有必要的。

已经有部分研究开始关注 UAVS 应对扰动的方法，并提出了相关指标。文献[13]将抗毁性引入多智能体系统中，通过 K-连通性和系统能耗两个标准进行抗毁性设计。文献[14]提出了 UAVS 鲁棒性指标，用于衡量集群遭受攻击后维持原有功能的能力。文献[15－16]分别从 UAV 自身和集群网络拓扑两方面分析了 UAVS 的可靠性。上述几类评估指标从不同维度对 UAVS 承受扰动的能力进行了描述，但并不能反映集群从遭受破坏到恢复性能的完整过程。

近年来，弹性概念开始逐步应用到 UAVS 研究中[17－18]。与上述指标相比，弹性考虑了系统从受损到恢复的全过程，可以更好地衡量应对方法的有效性，并对 UAVS 抵御干扰能力进行全面评估。

本文对 UAVS 弹性研究和发展进行了综述。首先介绍了 UAVS 的理论研究现状和相关概念，对弹性的应用领域进行了概述，进而提出了 UAVS 弹性的定义，并与近似指标进行了对比分析，说明了在 UAVS 中引入弹性的意义。进一步，对集群性能曲线、性能指标选取和弹性定量指标选取进行述评，对当前提升集群弹性的措施进行了概述，为 UAVS 弹性研究提供了一个较为完整的框架。最后，对集群弹性未来研究的主要问题和方向进行了讨论。

1　UAVS 复杂网络模型

通常基于复杂网络理论对 UAVS 进行建模，把各 UAV 视为节点，UAV 之间的联系视为边（本文假定 UAV 通信为双向链路），集群的动态变化视为复杂网络节点和边的变化，从而将集群系统视为一类复杂网络[13－14,17－18]。根据控制算法的不同[5－6,19]，集群网络的结构也会不同，一类简化的集群网络如图 1(a)所示。在扰动阶段，通过移除失效节点 v_i 和与之相连的边 $\{v_i, v_j\}$，建立集群遭受扰动时的模型，如图 1(b)所示，阴影节点为失效节点，虚线边为失效边。根据移除节点的重要性不同（通常按照节点度、介数、集聚系数等分类）[20]，扰动对于集群的破坏程度也不同。图 1(c)表示集群一类恢复模型，红边为增加边。将图论引入 UAVS 网络研究，可以对 UAVS 网络进一步分析[21－24]。集群网络中的节点和边组成顶点集 V 和边集 E。继而，UAVS 可以抽象表示为图 $G=\{V,E\}$。在集群中，UAV 只与自身通信范围内的 UAV 进行信息交互，因此 UAVS 网络拓扑又可归类于邻近图[25]，即边 $\{v_i, v_j\} \in E$ 当且仅当 v_j 处于 v_i 邻域内。通过拉普拉斯矩阵[23,26]等工具，可以对集群性质进行有效分析。

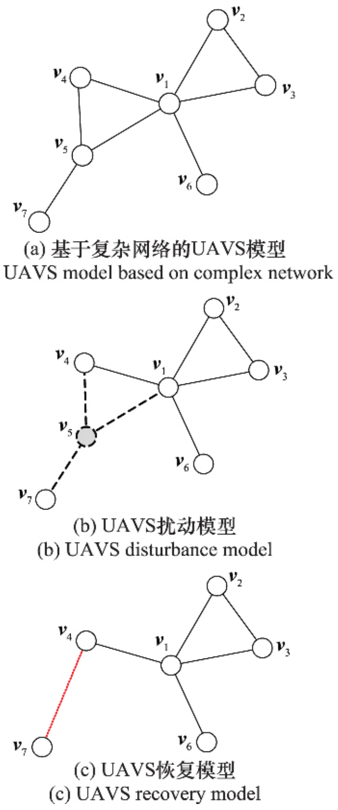

图 1　UAVS 系统建模

Fig. 1　UAVS system modeling

2　UAVS 弹性的概念

弹性用于衡量系统或者实体在遭受破坏后恢复到正常状态的能力[27]。弹性概念由生态学家 Holling 提出[28]，并将其引入到工程领域[29]，随后开始应用到基础设施[30]、通信网络[31]、物流网络[32]等领域，将其建模为复杂网络进行弹性研究[33]。弹性指系统对变化因素有准备和规划，可以承受、吸收扰动，并具有从扰动中恢复的能力[34]。

近年来，随着 UAVS 研究逐步由理论走向实践，扰动也成为 UAVS 实际应用中需要考虑的重要因素。UAVS 自组织特性使得集群对于扰动具有一定适应性和恢复力。传统的可靠性设计并不能预测复杂环境中的未知扰动，且不具备成本效益[35]。鲁棒性和抗毁性指标并不能反映集群自适应和恢复能力。弹性可以对系统应对未知扰动以及系统从扰动中恢复的能力进行评估，因此将弹性概念引入 UAVS 中更为合适。

UAVS 弹性指的是集群在遭受扰动（例如电磁干扰、组件故障）的情况下，能否及时降低损失，并尽快恢复至预期性能的能力。从广义上讲，UAVS 弹性包含 UAV 自身弹性和集群网络拓扑弹性两部分。UAV 自身弹性指 UAV 个体抵御扰动并从扰动中恢复至理想状态的能力；UAVS 网络拓扑弹性指集群网络拓扑吸收扰动，并能从扰动产生

(C)1994-2022 China Academic Journal Electronic Publishing House. All rights reserved.　http://www.cnki.net

的破坏中及时恢复到预期性能的能力。由于 UAVS 的理论研究通常把 UAV 视为智能体[36]，并没有太多考虑 UAV 自身变化，因此对于集群弹性的研究也主要关注较为狭义的集群弹性概念，即 UAVS 的网络拓扑弹性。本文中的 UAVS 弹性特指较为狭义的弹性概念。为便于理解，以第 1 节图 1 举例，集群在扰动阶段能保持的连通子图越大，在恢复阶段失联节点连接时间越短，说明该集群弹性越高。

在 UAVS 中，还存在除上述指标以外的生存性[31]、容错性[37]等属性。这些属性从不同角度对 UAVS 特性进行刻画，区分并不明显[31]。弹性与这些概念相比，既有重叠[27]，也有更进一步的发展。

在 UAVS 属性描述中，鲁棒性与弹性的区分最为关键。鲁棒性是 UAVS 中较为常见的度量指标，可以有效衡量集群在面临已知扰动中承受少量节点或者链路损失的能力，常用的鲁棒性指标通常综合节点度、聚类系数等网络参数进行计算[14]。通过增加节点和链路实现系统冗余等方法，集群可以达到很高的鲁棒性指标。然而，该集群并不一定拥有高弹性，因为鲁棒性指标并没有反映集群遭受扰动后的恢复时效和面对未知扰动时的性能变化。如果在发生较大规模破坏后，集群不能在有限时间内恢复拓扑，可以认为该集群弹性并不高。总的来说，相比于鲁棒性，弹性可以更加全面地反映集群受损和恢复的能力，可以评估应对威胁的各种措施，弹性概念涵盖了鲁棒性。

通过弹性指标可以对 UAVS 应对各类扰动的能力进行定量反映，继而可以研究不同网络和拓扑结构设计与恢复措施对于集群应对扰动的提升作用并进行比较，从而对集群对于扰动的应对能力进行总体评估，对于 UAVS 进一步走向实际应用具有推动作用。

3　UAVS 弹性评估方法

本节给出了 UAVS 性能变化曲线、性能选取指标以及弹性定量指标。

3.1　UAVS 性能变化曲线

UAVS 弹性研究主要关注两个部分：一是吸收扰动的能力，二是从扰动中恢复的能力。通过鲁棒性和快速性两个弹性属性，可以对上述两部分能力进行评估。鲁棒性在第 2 节中已有相关介绍，在此不做赘述；快速性用于衡量集群网络拓扑快速从遭受扰动恢复到预期性能的能力。结合上述两个属性，利用集群在扰动中的性能变化曲线，可以度量集群的弹性指标。

UAVS 在扰动过程中的典型性能变化曲线如图 2 所示[17-18]。在 t_0 时刻，当 UAVS 正常运行时，可以认为集群性能为 100%。在 t_1 时刻，集群受到扰动，性能会出现降级；在 t_{min} 时刻，集群性能到达最低值 Q_{min}。通过执行恢复策略或扰动减弱，性能逐渐上升，至 t_2 时刻恢复至稳定水平 Q_{final}，直到观察期 t_{end} 结束。

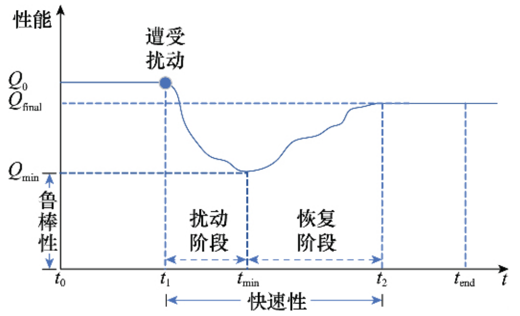

图 2　UAVS 扰动中的性能变化曲线

Fig. 2　Performance variation curve of UAVS in disturbance

3.2　UAVS 性能选取指标

在当前研究中，UAVS 性能通常指集群执行或完成特定任务的能力，例如在联合侦察任务中，用集群感知区域大小作为性能指标[18]；在搜索－攻击任务中，用战场覆盖率和目标存活时间衡量集群性能[38]。UAVS 通过内部的信息交互反馈实现自组织并执行任务，因此上述性能的选取可以认为是以集群的信息交换能力为基础[39]，结合特定任务分别增加相关指标。具体可表示为：UAVS 性能 Q＝集群信息交换能力 Q_E＋任务执行能力 Q_P。Q_E 一般以集群网络拓扑相关参数或集群内信息流量作为指标，文献[40－41]用网络连通性表达 Q_E，以此反映任务完成的可能性，文献[18]引入 UAV 数量(可抽象为节点数量)、UAV 邻居数量(可抽象为节点度)用于度量 Q_E，文献[17,42]则用集群内接收或交换的信息总量对 Q_E 进行评估。Q_P 则以具体任务设定指标，表 1 给出了在几类任务中 UAVS 的性能选取指标。

表 1　执行不同任务中 UAVS 性能指标选取

Table 1　Performance indicator selection for UAVS in different tasks

任务	性能选取指标		
	基础指标	体现方式	任务执行能力指标 Q_P
联合侦察	信息交换能力 Q_E	UAV 通讯邻居数量	UAV 数量、UAV 侦察范围
战场监视	信息交换能力 Q_E	最短路径长度、当前 UAV 数量、UAV 接收信息总量	——
搜索－救援	信息交换能力 Q_E	——	幸存者数量、搜救时间等
搜索－攻击	信息交换能力 Q_E	——	战场覆盖率、已发现目标的生存时间

(C)1994-2022 China Academic Journal Electronic Publishing House. All rights reserved.　　http://www.cnki.net

3.3 适用于 UAVS 的弹性定量指标

3.3.1 以网络拓扑参数定义弹性指标

该方法通过选取网络拓扑中可承受的最大故障节点数[43]、受扰动后的最大连通子图相对大小[44]等参数的函数对网络弹性进行度量。这类弹性研究表述方式简单直观，可以体现网络在受扰动后的相关参数变化趋势，但该方法没有考虑 UAVS 特性，且无法反映网络的恢复过程，仅适用于从复杂网络模型角度进行简单弹性分析。文献[45]在最大连通子图的基础上作出了改进，但是仍然无法体现弹性的完整定义。

3.3.2 以性能累积变化定义弹性指标

此方法将弹性指标定义为网络在观察期内的总体性能变化，用积分形式[46]表示为

$$R_1=\frac{\int_{t_0}^{t_{\mathrm{end}}}Q(t)\,\mathrm{d}t}{t_{\mathrm{end}}-t_0} \tag{1}$$

在实验环境中，$Q(t)$通过离散时间步长获得，因此上述弹性还可以表示为

$$R_2=\frac{\sum_{t_0}^{t_{\mathrm{end}}}Q(t)}{t_{\mathrm{end}}-t_0} \tag{2}$$

以性能累积变化的弹性指标反映了性能的动态变化，可以完整地体现网络吸收扰动和从扰动中恢复的过程，普遍运用于系统弹性度量，但单纯的指标值并不能精确地反映鲁棒性和快速性，需要结合性能变化曲线考察。

3.3.3 考虑鲁棒性和快速性的弹性指标

该指标基于性能变化曲线，综合考虑了集群网络的总体性能变化 α、鲁棒性 β、快速性 τ 和恢复度 γ。其中，

$$\alpha=\frac{\int_{t_0}^{t_{\mathrm{end}}}Q(t)\,\mathrm{d}t}{Q_0(t_{\mathrm{end}}-t_0)} \tag{3}$$

$$\beta=\frac{Q_{\min}}{Q_o} \tag{4}$$

$$\tau=\frac{t_2-t_1}{t_{\mathrm{end}}-t_0} \tag{5}$$

$$\gamma=\frac{Q_{\mathrm{final}}}{Q_o} \tag{6}$$

文献[18]分别从扰动阶段和恢复阶段进行考虑，将 α 分为扰动时的性能变化α_1 和恢复时的性能变化α_2，将 τ 表示为吸收快速性τ_1 和恢复快速性τ_2，进而将弹性指标定义为

$$R_3=\alpha_1\ \tau_1\beta+\alpha_2\ \tau_2\gamma \tag{7}$$

Tran 等人[39]考虑到了网络的波动性，提出了波动因子 θ，并将弹性指标表述为

$$R_4=\begin{cases}\alpha\gamma[\beta+\theta+1-\tau^{(\gamma-\beta)}],\ \gamma\geqslant\beta\\ \alpha\gamma(\beta+\theta),\ \text{其他}\end{cases} \tag{8}$$

对于遭受多次扰动的情况，可以对每次扰动时的系统弹性值加权求和得出总弹性值。此类指标可以较为完整准确地体现集群弹性的定义。

在后续的研究中，可以在上述主要基于复杂网络的弹性研究基础上，进一步纳入 UAV 自身特征，例如 UAV 通信功率调整成本对集群续航的影响等。

4 提升集群弹性的措施

扰动会影响集群网络的信息交换能力，导致网络中的节点和链路出现缺失，进而影响集群性能。根据 UAVS 弹性定义，从提高吸收扰动能力和恢复能力两方面对提升措施进行阐述。

4.1 提高集群吸收扰动能力的措施

提高集群吸收扰动能力主要指采取相关策略，尽可能减少集群受损范围，保持预期性能。根据遭受扰动时间点的不同，本节从扰动发生前和扰动阶段对提高吸收能力进行概述。

扰动发生前采取的措施主要是对网络拓扑结构进行优化。一是将集群进行分区，每个区根据相关指标动态选取该区域的关键节点，其余节点作为边缘节点，边缘节点仅与该区的关键节点进行交互[22,47]，以获得更灵活的通信结构，实现更高的连通性。另外，通过提高关键节点间的通信冗余度，可以进一步提高网络拓扑鲁棒性[48]。二是采用切换通信拓扑结构，当扰动造成拓扑变化时，可以及时建立新的可靠连接[49]。

在 UAVS 中，UAV 之间通过自组网技术[50－52]进行信息交换，因此网络的性能与网络拓扑结构变化密切相关。在扰动阶段，通过引入链路状态预测和中断/延迟容忍网络(delay tolerant networks，DTN)，改善扰动状态下的集群网络性能，进而提高网络拓扑结构的鲁棒性。链路状态预测用于发现网络中的连接情况[53]、链路质量[54]和链路稳定性[55]，进而采用链路优化等方式进行重连。近年，机器学习[56]、深度学习[57]方法开始引入链路预测中，用于分析未知扰动和拓扑发生频繁变化的网络。DTN 通过“摆渡”节点执行“存储－携带－转发”协议实现受限网络中的信息传递[58]，适用于高延迟、不可靠连接网络，对于解决 UAVS 网络因高速运动、复杂环境扰动造成的拓扑变化、通信中断具有很强的实用意义。

4.2 提高集群恢复能力的措施

提高恢复能力是指在恢复阶段，UAVS 执行相关策略，尽快达到或者接近预期性能。UAVS 自组织算法允许 UAVS 在出现个别 UAV 或链路故障时逐步恢复到新的稳定状态[59]。采用相关恢复策略可以进一步提高集群恢复能力。一是执行边重连策略[26]，失联 UAV 采用随机连接到附近 UAV、优先加入节点度较高的 UAV 等方式重新加入集群。二是采用链路评估算法，调整节点度分布使集群趋于稳定[21]。上述两类方式主要通过提高 UAV 通信功率实现，图 1(c)显示了 v_5 失效后，孤立节点v_7 通过增大功率与节点v_4 相连以重新加入集群。三是采用自修复算法，检测损坏 UAV，采用递归修复[22,60]、冗余节点备份[61]等方式用边缘 UAV、备份 UAV 代替损坏的关键节点 UAV。

(C)1994-2022 China Academic Journal Electronic Publishing House. All rights reserved.　http://www.cnki.net

通过防护策略和恢复策略的配合，UAVS 可以有效提高鲁棒性和快速性，从而提高 UAVS 弹性。

5 未来研究关键问题和主要方向

UAVS 领域相关研究方兴未艾，对于 UAVS 系统弹性研究也正处于起步阶段。针对当前有限的 UAVS 系统弹性研究现状，提出以下关键问题及研究方向，以期为后续研究提供参考，推动 UAVS 真正走向实际运用。

5.1 对集群遭受大规模破坏和集群崩溃边界的研究

崩溃边界是指集群可以承受的扰动范围，即最多可以接受多少节点和链路的损失，剩余节点在有限时间内仍可以通过相关策略继续执行并完成任务。

在当前的研究中，对于 UAVS 所遭受的扰动主要从集群小规模受损方面考虑。然而，UAVS 会用于执行一些危险任务，在执行这些任务的过程中，不乏一些会对集群造成重大破坏的因素（例如饱和攻击、电磁干扰），导致集群出现大规模的节点失效和链路中断，迅速达到崩溃边界。集群是否可以从这样的破坏中恢复预期性能，同样也是弹性的研究内容。

5.2 UAVS 特性与复杂网络的结合

对于 UAVS 弹性的研究方法目前还处于理想化阶段，主要采用复杂网络的方式进行研究，每个 UAV 通常都被抽象成为一个节点。但在实际应用中，UAVS 需要考虑诸多约束条件，例如有限的通信范围、UAV 动力学模型[62－63]、姿态控制、户外风扰动[64]等。此外，对于集群的扰动也通常从复杂网络角度出发，按照节点的重要性，将扰动分为随机失效和恶意攻击。但是现实中的扰动（如导弹攻击）会对整个杀伤半径内的 UAV 造成伤害，此时应更多考虑将集群自组织特性应用到集群恢复策略中。

5.3 集群弹性的整体评估方法

在 UAVS 中引入弹性概念不仅是为了衡量集群在特定任务中对于扰动的响应，更是为了从整体上描述集群应对各项扰动的能力。事实上，一类完整的任务执行过程不仅指到达指定地点执行指定任务，还需要完成准备阶段的投放集结、协同控制、路径规划、避碰避障等任务。在这些准备阶段，集群同样面临扰动，而目前的研究主要集中于任务执行阶段。整体评估就是要从整个任务执行过程出发，根据集群在每个阶段的行为特征，构造完整的扰动场景集合，对集群弹性进行评估。

6 结　论

随着 UAVS 逐步由理论走向实践，UAVS 受到的各种扰动也开始成为研究热点。本文总结了复杂网络弹性的相关文献，并应用于 UAVS 中，介绍了集群弹性的概念、意义、评估方法以及防护和恢复措施。针对现有集群弹性评估、恢复策略等研究应用于实际场景和重大破坏时的不足，本文提出了 UAVS 的关键问题和可能的研究方向，为后续的研究提供了相关参考。

参考文献：

[1] 梁晓龙，张桂强，吕娜．无人机集群[M]．西安：西北工业大学出版社，2018.
LIANG X L, ZHANG G Q, LYU N. UAV Swarms[M]. Xi'an: Northwestern Polytechnic University Press, 2018.

[2] 段海滨，张岱峰，范彦铭，等．从狼群智能到无人机集群协同决策[J]．中国科学：信息科学，2019，49(1)：112－118.
DUAN H B, ZHANG D F, FAN Y M, et al. From wolf pack intelligence to UAV swarm cooperative decision-making[J]. SCIENTIA SINICA Informationis, 2019, 49(1): 112－118.

[3] 贾永楠，田似营，李擎．无人机集群研究进展综述[J]．航空学报，2020，41(S1)：723738.
JIA Y N, TIAN S Y, LI Q. The development of unmanned aerial vehicle swarms[J]. Acta Aeronautica ET Astronautica Sinica, 2020, 41(S1): 723738.

[4] GU J J, SU T, WANG Q H, et al. Multiple moving targets surveillance based on a cooperative network for multi-UAV[J]. IEEE Communications Magazine, 2018, 56(4): 82－89.

[5] SUN Y P, WANG Z J, SU H S, et al. A brief overview of flocking control for multi-agent systems[C]//Proc. of the 11th International Conference on Intelligent Robotics and Applications, 2018: 48－58.

[6] OH K K, PARK M C, AHN H S. A survey of multi-agent formation control[J]. Automatica, 2015, 53: 424－440.

[7] PENG R. Joint routing and aborting optimization of cooperative unmanned aerial vehicles[J]. Reliability Engineering and System Safety, 2018, 177: 131－137.

[8] CEKMEZ U, OZSIGINAN M, SAHINGOZ O K. Multi-UAV path planning with multi colony ant optimization[C]//Proc. of the 17th International Conference on Intelligent Systems Design and Applications, 2017: 407－417.

[9] BERNARDINI S, FOX M, LONG D. Combining temporal planning with probabilistic reasoning for autonomous surveillance missions[J]. Autonomous Robots, 2015, 41(1): 181－203.

[10] SAMPEDRO C, BAVLE H, SANCHEZ J L, et al. A flexible and dynamic mission planning architecture for UAV swarm coordination[C]//Proc. of the International Conference on Unmanned Aircraft Systems, 2016: 355－363.

[11] LIU J J, WANG W P, LI X B, et al. Solving a multi-objective mission planning problem for UAV swarms with an improved NSGA-III algorithm[J]. International Journal of Computational Intelligence Systems, 2018, 11(1): 1067－1081.

[12] 刘献伟，陈虎林，李飞，等．一网打尽——无人集群时代的新攻防[J]．航空兵器，2019，26(1)：70－75.
LIU X W, CHEN H L, LI F, et al. Catch all in nets: new attack and defense in the age of unmanned cluster[J]. Aero Weaponry, 2019, 26(1): 70－75.

[13] 王强．面向任务的多智能体系统抗毁性拓扑结构构建与群集控制[D]．北京：北京理工大学，2014.
WANG Q. Task-oriented fault-tolerant topology and flocking control for multi-agent systems[D]. Beijing: Beijing Institute

(C)1994-2022 China Academic Journal Electronic Publishing House. All rights reserved. http://www.cnki.net

of Technology, 2014.

[14] WANG X H, ZHANG Y, WANG L G, et al. Robustness evaluation method for unmanned aerial vehicle swarms based on complex network theory[J]. Chinese Journal of Aeronaut, 2020, 33(1): 352－364.

[15] PETRITOLI E, LECCSE F, CIANI L. Reliability assessment of UAV systems[C]//Proc. of the IEEE International Workshop on Metrology for AeroSpace, 2017: 266－270.

[16] CUI X Y, WANG S P, SHI S A, et al. A delay-based reliability assessment for multiUAV with time-varying topologies[C]//Proc. of the International Conference on Aircraft Utility Systems, 2018.

[17] BAI G H, LI Y J, FANG Y N, et al. Network approach for resilience evaluation of a UAV swarm by considering communication limits[J]. Reliability Engineering and System Safety, 2020, 193: 106602.

[18] CHENG C C, BAI G H, ZHANG Y A, et al. Resilience evaluation for UAV swarm performing joint reconnaissance mission[J]. Chaos, 2019, 29(5): 053132.

[19] WANG L, LU D, ZHANG Y, et al. A complex network theory-based modeling framework for unmanned aerial vehicle swarms[J]. Sensors, 2018, 18(10): 3434.

[20] 何明, 马子玉, 刘锦涛, 等. 基于影响度介数中心性的多智能体牵制控制算法[J]. 控制与决策, 已录用.
HE M, MA Z Y, LIU J T, et al. Multi-agent pinning control algorithm based on betweenness centrality with influence degree[J]. Control and Decision, accepted.

[21] 陈昕, 范铭楷, 李泽宏, 等. 蜂群无人机系统的网络鲁棒性设计[J]. 系统工程与电子技术, 2019, 41(11): 2633－2640.
CHEN W, FAN M K, LI Z H, et al. Design of network robustness for drone swarm system[J]. Systems Engineering and Electronics, 2019, 41(11): 2633－2640.

[22] 符小卫, 魏可, 李斌, 等. 基于联盟的无人机集群编队控制方法[J]. 系统工程与电子技术, 2019, 41(11): 2559－2572.
FU X W, WEI K, LI B, et al. Formation control method of UAVs based on alliance[J]. Systems Engineering and Electronics: 2019, 41(11): 2559－2572.

[23] OLFATI-SABER R. Flocking for multi-agent dynamic systems: algorithms and theory[J]. IEEE Trans. on Automatic Control, 2006, 51(3): 401－420.

[24] SAMPEDRO C, BAVLE H, SANCHEZ-LOPEZ J L, et al. A flexible and dynamic mission planning architecture for UAV swarm coordination[C]//Proc. of the International Conference on Unmanned Aircraft Systems, 2016: 355－363.

[25] LU G, ZHOU M T, NIU X Z, et al. A survey of proximity graphs in wireless networks[J]. Journal of Software, 2008, 19(4): 888－911.

[26] CHEN P Y, HERO A O. Assessing and safeguarding network resilience to nodal attacks[J]. IEEE Communications Magazine, 2014, 52(11): 138－143.

[27] HOSSEINI S, BARKER K, RAMIREZ-MARQUEZ J E. A review of definitions and measures of system resilience[J]. Reliability Engineering and System Safety, 2015, 145: 47－61.

[28] HOLLING C S. Resilience and stability of ecological systems[J]. Annual Review of Ecology and Systematics, 1973, 4: 1－23.

[29] FRANCIS R, BEKERA B. A metric and frameworks for resilience analysis of engineered and infrastructure systems[J]. Reliability Engineering and System Safety, 2014, 121: 90－103.

[30] TURNQUIST M, VUGRIN E, Design for resilience in infrastructure distribution networks[J]. Environment Systems and Decisions, 2013, 33(1): 104－120.

[31] STERBENZ J P G, HUTCHISON D, CETINKAYA E K, et al. Resilience and survivability in communication networks: strategies, principles, and survey of disciplines[J]. Computer Networks, 2010, 54(8): 1245－1265.

[32] 颜炳莅. 分形物流网络拓扑弹性研究[D]. 武汉: 武汉理工大学, 2016.
YAN B L. Research on topological resilience of fractal logistics networks[D]. Wuhan: Wuhan University of Technology, 2016.

[33] GAO J, LIU X, LI D, et al. Recent progress on the resilience of complex networks[J]. Energies, 2015, 8(10): 12187－12210.

[34] Department of Homeland Security. Critical infrastructure security and resilience functional relationships[R]. Washington DC: Department of Homeland Security, 2013.

[35] TRAN H T, DOMERCANT J C, MAVRIS D N. A network-based cost comparison of resilient and robust system-of-systems[J]. Procedia Computer Science, 2016, 95: 126－133.

[36] 孙强, 梁晓龙, 尹忠海, 等. UAV 集群自组织飞行建模与控制策略研究[J]. 系统工程与电子技术, 2016, 38(7): 1649－1653.
SUN Q, LIANG X L, YIN Z H, et al. UAV swarm self-organized flight modeling and control strategy[J]. Systems Engineering and Electronics: 2016, 38(7): 1649－1653.

[37] BJERKNES J D, WINFIELD A F T. On fault tolerance and scalability of swarm robotic systems[M]. Heidelberg: Springer, 2013.

[38] GAO C, ZHEN Z Y, GONG H J. A self-organized search and attack algorithm for multiple unmanned aerial vehicles[J]. Aerospace Science and Technology, 2016, 54: 229－240.

[39] TRAN H T, BALCHANOS M, DOMERCANT J C, et al. A framework for the quantitative assessment of performance-based system resilience[J]. Reliability Engineering and System Safety, 2016, 158: 73－84.

[40] WANG L Z, ZHAO X J, ZHANG Y, et al. Unmanned aerial vehicle swarm mission reliability modeling and evaluation method oriented to systematic and networked mission[J]. Chinese Journal of Aeronautics, accepted.

[41] AKSARAY D, YASIN Y A, FERON E, et al. Message-passing strategy for decentralized connectivity maintenance in multiagent surveillance[J]. Journal of Guidance Control and Dynamics, 2013, 39(3): 11153303.

[42] KURDI H, HOW J, BAUTISTA G. Bio-inspired algorithm for task allocation in multi-UAV search and rescue missions[C]//Proc. of the AIAA Guidance, Navigation, and Control Conference, 2016.

[43] NAJJAR W, GAUDIOT J L. Network resilience: a measure of network fault tolerance[J]. IEEE Trans. on Computers, 1990, 39(2):174－181.

[44] XIAO Y D, LAO S Y, HOU L L, et al. Mitigation of mali-

(C)1994-2022 China Academic Journal Electronic Publishing House. All rights reserved. http://www.cnki.net

cious attacks on network observation[J]. International Journal of Modern Physics C, 2015, 26(10):1550108.
[45] BHATIA U, KUAMR D, KODRA E, et al. Network science based quantification of resilience demonstrated on the Indian Railways Network[J]. PlosOne, 2015,10(11): e0142890.
[46] REED D A, KAPUR K C, CHRISTIE R D. Methodology for assessing the resilience of networked infrastructure[J]. IEEE Systems Journal, 2009, 3(2): 174-180.
[47] CHEN S M, HUA Y X, ZHU Z M, et al. Fast flocking algorithm for multi-agent systems by optimizing local interactive topology[J]. Acta Automatica Sinica, 2015, 41(12): 2092-2099.
[48] ZHAO H L, YANG H T, FU Y. Analysis method of resilience in networked command and control information system[J]. Journal of Command and Control, 2015, 1(1):14-18.
[49] MA S Q, DONG C Y, MA M Y, et al. Formation reconfiguration control of quadrotor UAVs based on adaptive communication topology[J]. Journal of Beijing University of Aeronautics and Astronautics, 2018, 44(4): 841-850.
[50] BEKMEZCI I, SAHINGOZ O K, TEMEL Ş. Flying ad-hoc networks (FANETs): a survey[J]. Ad Hoc Networks, 2013, 11(3): 1254-1270.
[51] XIONG F, LI A, WANG H, et al. An SDN-MQTT based communication system for battlefield UAV swarms[J]. IEEE Communications Magazine, 2019, 57(8): 41-47.
[52] **文少杰，黄传河. FANET 中时延感知的垮层优化方法**[J]. **通信学报**, 2018, 39(4): 2018070.
WEN S J, HUANG C H. Delay-aware cross-layer optimization method for FANET[J]. Journal on Communications, 2018, 39(4): 2018070.
[53] **王凯，刘树新，陈鸿昶，等. 一种基于节点间资源承载度的链路预测方法**[J]. **电子与信息学报**, 2019, 41(5): 214-223.
WANG K, LIU S X, CHEN H X, et al. A new link prediction method for complex networks based on resources carrying capacity between nodes[J]. Journal of Electronics and Information Technology, 2019, 41(5): 214-223.
[54] SHU J, LIU M L, SHANG Y Q, et al. Link quality prediction model based on Gaussian process regression[J]. Journal on Communications, 2018, 39(7): 148-156.
[55] **胡曦，李喆，刘军. 移动 Ad hoc 网络中基于链路和稳定性预测的按压路由协议**[J]. **电子与信息学报**, 2010, 32(2): 284-289.
HU X, LI Z, LIU J. A link stability prediction-based on-demand routing protocol in mobile Ad hoc networks[J]. Journal of Electronics and Information Technology, 2010, 32(2): 284-289.
[56] **张薇玮，丁文锐，刘春辉. 复杂环境中无人机数据链干扰效果预测方法**[J]. **系统工程与电子技术**, 2016, 38(4): 760-766.
ZHANG W W, DING W R, LIU C H. Prediction of interference effect on UAV data link in complex environment[J]. Systems Engineering and Electronics, 2016, 38(4): 760-766.
[57] SHU J, ZHANG X P, LIU L L, et al. Multi-nodes link prediction method based on deep convolution neural networks[J]. Acta Electronica Sinica, 2018, 46(12): 2970-2977.
[58] GONCALVES F J, PATEL A, BATISTA B L A, et al. A systematic technical survey of DTN and VDTN routing protocols[J]. Computer Standards and Interfaces, 2016, 48: 139-159.
[59] **柳强，何明，刘锦涛，等. 无人机"蜂群"的蜂拥涌现行为识别与抑制机理**[J]. **电子学报**, 2019, 47(2): 374-381.
LIU Q, HE M, LIU J T, et al. A mechanism for identifying and suppressing the emergent flocking behaviors of UAV swarms[J]. Acta Electronica Sinica, 2019, 47(2): 374-381.
[60] **张飞，陈卫东. 移动机器人编队自修复的切换拓扑控制**[J]. **控制理论与应用**, 2010, 27(3): 289-295.
ZHANG F, CHEN W D. Switched topology control for self-healing of mobile robot formation[J]. Control Theoryand Applications, 2010, 27(3): 289-295.
[61] **何明，梁文辉，陈秋丽，等. 水下水下移动无线传感器网络拓扑愈合与优化**[J]. **控制与决策**, 2015,2: 251-255.
HE M, LIANG W H, CHEN Q L, et al. Topology self-healing algorithm of mobile underwater wireless sensor networks[J]. Control and Decision, 2015,2: 251-255.
[62] Al-SHABI M A, HATAMLEH K S, ASAD A. UAV dynamics model parameters estimation techniques: a comparison study[C]// Proc. of the Applied Electrical Engineering and Computing Technologies, 2013.
[63] CANTARELO O C, ROLLAND L, O'YOUNG S. Validation discussion of an unmanned aerial vehicle(UAV) using JSBSim flight dynamics model compared to Matlab/Simulink AeroSim blockset[C]// Proc. of the IEEE International Conference on Systems, Man, and Cybernetics, 2016: 3989-3994.
[64] **代波，何玉庆，谷丰，等. 基于加速度反馈增强的旋翼无人机抗风扰控制**[J]. **机器人**, 2020, 42(1): 79-88.
DAI B, HE Y Q, GU F, et al. Acceleration feedback enhanced controller for wind disturbance rejection of rotor unmanned aerial vehicle[J]. Robot, 2020, 42(1): 79-88.

作者简介：

顾凌枫(1991-)，**男，硕士研究生，主要研究方向为无人机指控。**
E-mail:LingFeng802@126.com
何　明(1978-)，**男，教授，博士研究生导师，博士，主要研究方向为智联网与无人机指控。**
E-mail:paper_review@126.com
陈国友(1973-)，**男，副教授，硕士，主要研究方向为通信工程。**
E-mail:49862832@qq.com
季敏惠(1985-)，**女，工程师，硕士，主要研究方向为网络通信。**
E-mail:240793335@qq.com
刘锦涛(1981-)，**男，工程师，博士，主要研究方向为多智能体协同控制。**
E-mail:top1944@163.com

(C)1994-2022 China Academic Journal Electronic Publishing House. All rights reserved. http://www.cnki.net
