# Supplementary material for: A game-based approach for designing a collaborative evolution mechanism for unmanned swarms on community networks
Source: Sci Rep. 2022 Nov 7;12:18892. doi: 10.1038/s41598-022-22365-z (PMC9640601; doi:10.1038/s41598-022-22365-z)
Supplement: Supplementary file 1 — Supplementary Information. [file 41598_2022_22365_MOESM1_ESM.zip › Supporting material/7.í╢Research on Countermeasures Equipment Demand and Coping Strategies for Maritime Small UAV Swarmsú¿║ú╔╧╨í╨═╬▐╚╦╗·╝»╚║╡─╖┤╓╞╫░▒╕╨Φ╟≤╙δ╙a╢╘╓«▓▀╤╨╛┐ú⌐í╖.pdf]

海上小型无人机集群的反制装备需求与应对之策研究

柳强[1,2] 何明[1]
(1.陆军工程大学 指挥控制工程学院,江苏 南京 210007; 2.海军指挥学院,江苏 南京 210000)

摘 要: 为提高海上应对小型无人机集群的响应处置能力,首次将小型无人机集群作为新型空中威胁对象进行分析,研究其在海战场环境下的作战模式、组织运用方式以及战技术性能,探究其遂行完成既定任务对我海上作战行动所产生的不利影响。运用定性与定量分析相结合的方法,对现有的舰载探测系统、交战系统在未来反集群作战的能力现状进行分析,并从预警探测、通信干扰、伪装欺骗和毁伤拦截四个方面提出反制装备建设需求。结合海战场环境与海上兵力运用的特点,从作战指挥体系、预警侦察体系和反制拦截体系构建三个方面积极寻找应对之策。采用离散事件仿真方法对"宙斯盾"舰反集群作战过程进行动态模拟,通过仿真实验,分析和讨论了舰载探测跟踪性能、毁伤拦截性能等因素对集群突防能力的影响,并指出建立梯次纵深的预警探测区和火力拦截区具有一定的必要性。

关键词: 集群; 小型无人机集群; 反制装备; 反集群作战
中图分类号: V279; E917 **文献标志码:** A **文章编号:** 1672-8211(2019) 04-0059-07

1 引言

近年来,集群概念逐步推广到军事作战领域。早在 2014 年,美军就利用这一概念先后启动了小精灵(Gremlins) 、山鹑(Perdix) 、低成本无人机集群技术(LOCUST) 、进攻性蜂群赋能技术(OFFSET) 等多个无人机集群项目,各项目在功能上互补、各有侧重,标志着美军的集群作战技术已正式从理论研究转入研发阶段[1-3]。

作为一门新型技术,无人机集群无疑是一把"利刃",对防御方构成新的空中威胁。为此,一些反无人机的技术与装备也应运而生。根据 2018 年 2 月美国巴德学院无人机研究中心发布的一份研究报告称[4],国外已有超过 230 种系统用于反无人机的探测与拦截,其中 88 种专门用于探测无人机,80 种专门用于拦截无人机,而另外 67 种则集探测和拦截无人机功能于一身,这些系统的特点是采用电子压制技术对无人机实施电子软杀伤。值得注意的是,当来袭的小型无人机比较多(数十架甚至上百架) ,且采用无线组网方式形成小型无人机集群时,上述提到的反无人机系统很可能无法抵消集群在数量上的优势,呈现饱和状态,从而导致反制措施无效。在应对无人机集群这类新型威胁时,一方面是要提高单个反制装备的探测性能与拦截能力,另一方面是要采用科学、合理的分散部署方式来解决单一反制装备抗饱和能力不足的问题。同时,与陆上反制小型无人机集群的情况相比,海上应对小型无人机集群威胁的形势更为严峻: ①反制装备部署方式受到限制,无法在战场前沿和纵深采用固定站的方式进行超前部署; ②海洋水文气象环

收稿日期: 2019-04-30; 修回日期: 2019-08-23
基金项目: 国家重点研发计划(2018YFC0806900、2016YFC0800606、2016YFC0800310) ; 江苏省自然科学基金(BK20161469) ; 江苏省重点研发计划(BE2016904、BE2017616、BE2018754)
作者简介: 柳强(1983—) ,男,讲师,博士研究生,主要研究方向为指挥控制系统工程; 何明(1978—) ,男,教授,博士,博士生导师,主要研究方向为指挥控制理论与技术。

(C)1994-2020 China Academic Journal Electronic Publishing House. All rights reserved. http://www.cnki.net

境复杂多变，反制装备的探测性能、指挥通信链路以及反制手段的使用均会受到影响；③海上作战舰艇留给反制装备部署的软硬件环境空间有限，极有可能要考虑拦截武器数量上限、电子压制实施过程中的电磁兼容等问题，这将导致反制装备的效能无法得到充分发挥。

目前，国内外关于海上反制小型无人机集群的研究尚不多见。为提高海上应对小型无人机集群威胁的处置能力，本文根据小型无人机集群的系统组成、技战术性能、兵力编组与战术运用特点，探究其对我未来海上防空作战行动的影响，对海上反制小型无人机集群的装备能力现状与差距进行定性分析，提出相应的反制装备能力需求，并从作战指挥体系、预警侦察体系和反制拦截体系构建三个方面积极寻找海上反集群作战应对之策。最后，以美国最先进的“宙斯盾”舰为例，采用离散事件仿真方法模拟水面舰艇反制小型无人机集群的动态过程，通过对比不同的反制装备性能改进方案来定量分析当前水面舰艇反集群作战的能力，为未来海上反制小型无人机集群的装备研制与建设提供决策建议。

2 小型无人机集群对海上作战行动的影响

2.1 小型无人机集群

近年来，随着小型化、智能化技术的不断发展，小型无人机日趋成熟，在战术组织运用的形式上，更多地采用网络化集群作战的新模式，由此产生了小型无人机集群。

在系统组成上，小型无人机集群一般由三部分组成，即无人机集群子系统、集群发射/回收子系统以及集群指挥控制子系统。其中，无人机集群子系统是由一群同构或异构的小型无人机组成，每个无人机都能利用自身搭载的侦察/成像载荷或电子战载荷感知战场环境，通过集群内部数据链彼此交互所感知的战场环境信息，遂行完成多样化军事任务；而集群发射/回收子系统则会部署在大型作战平台上（如空中运输机、集装车辆），并采用空中发射、空中抛射、管式发射等形式将小型无人机集群投放到指定作战空域；对于集群指挥控制子系统，则采用“人在回路上”的控制模式，即无人系统集群在交互回路中接收人下达的任务指令，自主完成任务；人只是在交互回路中监视与预测集群系统的运行状态与任务执行情况，必要时可对集群进行干预[5-7]。但这并不代表小型无人机集群可以完全脱离人的控制。对于携带致命武器的小型无人机集群来说，出于法律和道德层面的考虑，其控制方式也会严格限定为远程遥控方式，如“扣扳机”的动作指令仍然需要由人来下达。为此，未来的小型无人机集群的作战模式将是如图 1 所示的有人—无人协同作战模式，集群的指挥控制子系统部署在发射/回收集群的大型运载平台上，或者部署在与集群一同前出执行任务的有人战斗机上。

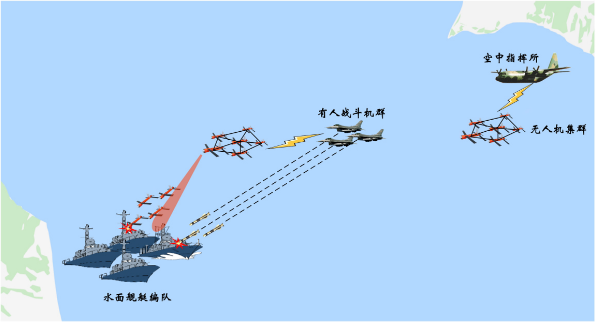

图 1 有人—无人协同作战模式概念图

2.2 战术运用模式及其影响

小型无人机集群可依托自身平台搭载的各类小型任务载荷担负情报侦察与战场监视、空中电子

表 1 美军典型集群无人机平台及技战术性能表

型号	重量(kg)	最大飞行速度(m/s)	最大飞行高度(m)	续航时间(h)	任务载荷	造价(万美元)	用途
灰山鹑(Perdix)	0.45	40	≤1000	0.3	射频	不详	ISR 电子打击
郊狼(Coyote)	5.9	30	6094	1.5	光电/红外	1.5	ISR 电子打击
小精灵(Gremlins)	320	238~272	9000	1~3	雷达、射频<br>光电/红外	≤60	ISR 电子打击

(C)1994-2020 China Academic Journal Electronic Publishing House. All rights reserved. http://www.cnki.net

压制与欺骗、自杀式饱和攻击等具体任务，表 1 列举了美军遂行完成上述特定任务的典型集群无人机平台以及其战技术性能[8-11]。在战术运用模式上，可将一定数量的小型无人机编组为具有特定作战用途的小型无人机侦察群、小型无人机伴动群、小型无人机攻击群或小型无人机察打群，从而参与海上进攻作战行动。

2.2.1 情报侦察与战场监视

利用前视红外仪、电视摄像机、电子侦察设备，小型无人机集群可对指定海域进行实时的空中协同侦察、监视，全面掌握敌方海上目标的兵力部署与作战行动情况。一般情况下，具备光学/红外侦察能力或电子侦察能力的无人机既可以独立编组为一个同构的小型集群，也可以与彼此具备不同侦察能力的无人机混合编组为一个异构的小型集群。这些集群各有优势，具备光学/红外侦察能力的小型同构集群可以在敌方采用电磁静默措施后继续对其实施抵近式侦察和监视，相比之下，具备电子侦察能力的小型同构集群可以在更远距离对敌方辐射源进行侦察定位；而异构集群则充分发挥了前两类同构集群的各自优势，将电子信号情报与图像情报作为查明敌方目标的相互印证手段。

由于小型无人机集群采用静默方式执行情报侦察与战场监视任务，加上小型无人机平台轻小，雷达散射截面积较小，具有较强的渗透性，这对于应对传统空中威胁的海上防空作战体系而言，进一步弱化了其预警侦察能力。而预警侦察是防空体系得到正常运转的重要前提，失去了预警侦察能力，整个海上防空作战行动将陷入被动。

2.2.2 空中电子压制与欺骗

若具备电子侦察能力的小型无人机集群在飞行平台上搭载了小型武器载荷，则进一步升级为压制或摧毁敌海上防空火力单元的理想武器。一方面，此类集群可利用电子侦察设备截获敌辐射源辐射的电磁信号，随后进行定位、摧毁；另一方面，若敌方为躲避集群侦收其辐射源信号而被迫采取电磁静默措施，使得集群达到了压制敌方防空系统的效果，为后续突击兵力采取攻击行动开辟空中走廊。此外，携带电子欺骗设备的小型无人机集群可以模拟有人战斗机机载雷达信号，或转发敌方雷达回波信号，引诱舰载火控雷达开机，吸引敌方防空火力，查明敌方海上防空力量部署与编成情况，为后续突击兵力采取攻击行动提供实时战场情报信息。

小型无人机集群的空中电子压制能力和欺骗能力将导致海上防空体系作战效能进一步降低。火控雷达是海上组织防空火力打击行动的关键组成部分，一旦采取电磁静默措施，防御方将完全陷入被动挨打的境地。同时，小型无人机集群内部可能含有具备红外侦察能力的无人机，可以无视防御方采取的电磁静默措施，采用红外寻的攻击方式，致使防御方海上防空武器生存更为困难。

2.2.3 自杀式饱和攻击

由于小型无人机集群所采用的作战平台造价成本低廉，是采用非对称方式攻击海上高价值目标（如航母）的理想作战应用模式。小型无人机集群可按照多方向、多批次方式进入敌方防空作战体系，对高价值目标实施自杀式饱和攻击。同时，小型无人机集群在对敌方海上目标实施首次突击后，可利用小型无人机集群携带的侦察/成像载荷照射目标，实时评估战损情况，以确定是否组织多轮突击行动，直至目标瘫痪或完全被摧毁。

小型无人机集群采用“以多打少”的对抗形式攻击海上重要目标，在数量上呈压倒性优势，这使得防御方的预警探测系统和防空火力系统在短时间内饱和。防御方需要将有限的防空系统（探测系统与武器系统）分散到多个方向、多个批次的来袭目标上，受武器装备性能影响，可能存在探测盲区或射击死区，防空武器的指挥控制难度进一步增加，难以招架规模较大的小型无人机集群。

3 海上小型无人机集群作战的反制装备需求分析

3.1 反制装备能力现状

目前，海上水面舰艇反制小型无人机集群的能力不容乐观。在探测跟踪能力方面，水面舰艇并没有列装用于探测小型无人机这类“低慢小”目标的专门设备，与反舰导弹或战斗机等常规空中威胁目标相比，若沿用传统的舰载搜索雷达或舰载光电探测系统，其对“低慢小”目标的发现距离相对较短，一般不会超过 10km，海上舰艇编队无法达成区域协同防空作战的条件，致使整个海上编队的一体化防空作战能力弱化为单舰防空作战能力。同时，探测能力不足也会进一步弱化海上编队对小型无人

(C)1994-2020 China Academic Journal Electronic Publishing House. All rights reserved. http://www.cnki.net

机集群威胁的早期预警能力，造成后续防空拦截系统组织反制措施的时间减少。在反制拦截方面，舰艇中程、近程防空武器并不是拦截小型无人机集群的理想武器。一是中程舰空导弹虽然具备精确打击能力，但相对于低成本、可消耗的小型无人机来说，无异于“大炮打蚊子”，效费比低；二是近防武器系统（如密集阵）和小型舰载武器（如舰炮）有效作用距离较短，命中率低，在单舰抗击小型无人机集群的情况下，极容易让防空火力通道饱和。

通过上述分析，探测跟踪系统对“低慢小”目标的早期发现能力仍然是海上能否在反集群作战中取得成功的关键，这也是防御方能否积极采取反制措施、合理配置反制作战资源的先决条件。

3.2 反制装备能力需求

为此，我们将重点从探测跟踪、电子干扰、伪装欺骗和毁伤拦截四个方面来探讨未来海上反集群作战装备的能力需求。

（1）探测跟踪能力。应积极研制固态多普勒、超材料电子扫描等新体制舰载雷达[12,13]，采用特殊的探测与跟踪算法，抑制海杂波，分离海面与空中目标。同时，在探测跟踪系统设计中，强化多源/多模信息融合能力，尽可能将雷达、光电传感器、红外传感器和无线电侦察传感器组合起来，形成信息情报相互印证手段，对“低慢小”目标的频率特征、电磁特征、图像特征进行综合识别，从而实现对小型无人机集群的持续探测与跟踪，为后期反制阶段留有足够的应急响应时间。

（2）通信干扰能力。小型无人机集群无论是进行图像/信号情报的信息融合，还是用于协调群体飞行的蜂拥控制，都离不开数据链的支持。同时，小型无人机集群还要通过数据链与有人控制站、有人战斗机群保持无线电通信联系，从而达到有人/无人协同作战的目的，这说明小型无人机集群对通信资源严重依赖。应重视具备强大通信干扰能力的装备建设，对敌方集群内部、集群之间以及集群与有人作战平台之间通联的数据链实施通信压制，从而降低甚至丧失其协同作战能力。

（3）伪装欺骗能力。对于攻击方而言，一般会利用外部手段（如天基探测系统）对海面大型目标概略定位后，再通过小型无人机集群遂行实时侦察任务。而这种实时侦察任务需要借助于集群平台搭载的侦察/成像载荷或电子侦察载荷完成，而电子侦察载荷相比侦察/成像载荷，能在更远的距离上获取海上情报，但这给防御方采用电子伪装欺骗技术提供了可乘之机。例如，依托具备伪装欺骗能力的多艘无人艇主动辐射舰载对空搜索雷达或火控雷达信号来构建“电子欺骗陷阱”，引诱敌方小型无人机集群进入防御方的防空火力打击范围，对其实施围歼。

（4）毁伤拦截能力。小型无人机集群采用低成本的非对称方式突击海上目标，而防御方除了使用传统火力毁伤武器作为反制集群的补充手段外，还应积极寻求性价比更高的、具备精确打击或者面杀伤能力的武器，如微型导弹或集束弹药[14,15]。其中，微型导弹是一种体积小、成本低、精度高的精确制导武器，一般采用可见光成像制导方式，具有“发射后不管”的使用特点，是打击“低慢小”目标的理想武器；而集束弹药是一种具备面杀伤能力的武器，拥有瞬时火力密集、覆盖面积广、毁伤效能大等特点，非常适合毁伤和拦截敌方空中集群目标。目前，美国海军研究生院 Christopher 研究团队也在致力于研究一款拦网式舰射集束导弹，极有可能在不久的将来列装舰艇部队使用[16,17]。

4 未来海上小型无人机集群应对策略

小型无人机集群作为改变战争游戏规则的颠覆性装备，在未来必然成为我海上难以应对的新型空中威胁，但并不表示在未来的防空作战中我们将毫无作为、束手就擒，应坚持以“体系破击战”的作战思想为指导，对抗对我实施海上打击行动的空中无人作战力量，建立科学、合理、高效的海上反集群作战指挥体系，完善和健全我海上反集群作战的预警侦察体系和反制拦截体系。

4.1 建立一体化、扁平化的反集群作战指挥体系

未来海战场情况变化快，决策时间短，实时性要求高，同时海上各兵力兵器的指挥与控制都需依赖信息化程度较高的各类电子信息系统，指挥与协同较为复杂，需要按照海上作战体系结构建立科学、合理、高效的反集群作战指挥体系，以适应新情况。①坚持按级指挥与越级指挥相结合。通过简化指挥层次，缩短指挥流程，建立一种扁平化的指挥结构，以减少因决策延误或指挥失效所导致的不良恶果；②重视电子战指挥体系的建立。未来海上

(C)1994-2020 China Academic Journal Electronic Publishing House. All rights reserved. http://www.cnki.net

作战将大量使用电子战飞机、电子对抗无人机、舰载综合电子战系统，所采取的各类电子战行动将贯穿其始终，若能借助先进的现代化手段指挥和控制各类电子战系统，将有力提高我海上反集群作战的电磁态势感知能力、电子对抗情报信息处理能力以及电子战指挥控制引导能力。

4.2 建立全方位、多层次的反集群预警侦察体系

小型无人机集群与传统的海上突击兵力不同，具有低可探测性，渗透效果好，必然会对我重点防护的高价值目标实施“以多打少”的饱和攻击。为此，亟须建立具有多层防御、综合立体的反集群预警侦察体系。首先，由于小型无人机集群现阶段仍然采用有人—无人协同作战模式，海战场环境中必然存在投放大量无人机的空中运输平台或者指挥集群作战的有人战斗机群，与“低慢小”目标相比，这些敌方目标雷达或红外特征明显，易于发现，目标价值同样重要，应立足现有预警侦察装备，加强对有人作战平台的早期预警，从而因势利导为后续反制作战行动提供有价值的信息情报。其次，应着手考虑在防空作战的中远区前出无人巡逻警戒兵力，综合运用雷达、光电/红外设备以及被动电子侦察设备查明周边海空域情况，一旦发现集群目标，立即发出预警信息。

4.3 建立综合化、专业化的反集群反制拦截体系

虽然小型无人机集群凭借数量上的优势，通过多点、多次、快速打击，致使防御方预警探测传感器和拦截武器系统在短时间内饱和，达成了小火力实现对时敏目标(如航母)的精确打击，起到了“四两拨千斤”的非对称作战效果。但小型无人机集群对通信网络和 GPS 导航系统的高度依赖却成为其难以躲避的技术软肋。因此，我们应运用“非对称作战”思维，重点从软对抗和硬对抗两个方面加强小型无人机集群对抗装备的建设。一是在软对抗方面，大力发展舰载电子战飞机、舰载电子对抗无人机，提升对小型无人机集群导航控制系统与数据链通信系统实施压制干扰的作战能力。二是在硬对抗方面，积极研发新型面杀伤集群拦截系统，以应对在拒止环境(通信和 GPS 受到干扰的环境)下仍能工作的小型无人机集群。同时，坚持“软硬抗击一体、点面杀伤结合”的思路，利用预警侦察体系获取的精确集群目标信息，判断来袭目标的攻击意图，依托一体化防空作战网络组织反制武器拦截集群目标，从而达到保护高价值目标的目的。

5 仿真与分析

5.1 仿真设置

本文运用 ExtendSim7.0 仿真软件对水面舰艇反制小型无人机集群这一动态过程进行模拟。在对抗过程中，假定小型无人机集群以低空匀速方式飞行，按照一定到达速率(默认为 3 架/s)进入海上舰艇目标的探测与毁伤拦截区，而舰艇目标则保持静止不动，并利用舰载探测系统对周边海空域进行监视预警，当发现小型无人机威胁时，立即通过舰载交战系统对其实施毁伤拦截。因舰载交战系统一般由多个近程防空武器组成，可同时对多个来袭的小型无人机实施火力拦截，故可将该交战过程抽象为 M/M/k 排队系统。

利用 ExtendSim7.0 仿真软件中自带的离散事件仿真功能模块建立小型无人机空袭流生成模型、预警探测模型和火力拦截模型。在建模过程中，结合未来海上反制装备的能力需求，考虑模型参数对舰艇反集群作战能力的影响。例如，增大探测距离、火力杀伤距离以及杀伤概率间接反映出反制装备探测跟踪能力和火力拦截能力的提升；而反制

表 2 美国“宙斯盾”舰反无人机装备性能对照表

	装备型号	装备性能
探测系统<br>(Detection Systems)	AN/SPY-1D(V)<br>AN/SPS-67<br>EOSS	0—250m，探测概率为 0.6<br>251—500m，探测概率为 0.5<br>501—1000m，探测概率为 0.4
交战系统<br>(Engagement Systems)	MK-38(x2)<br>M2HB(x4)<br>CIWS(x2)	0—250m，杀伤概率 0.1，射击周期 0.6s<br>251—500m，杀伤概率 0.15，射击周期 0.6s<br>251—500m，杀伤概率 0.3，射击周期 1.25s<br>500—750m，杀伤概率 0.2，射击周期 1.25s

(C)1994-2020 China Academic Journal Electronic Publishing House. All rights reserved. http://www.cnki.net

表 3 仿真评估方案

序号	仿真模型参数	备注
方案 1	—	以“宙斯盾”舰现有的装备性能进行评估
方案 2	CIWS 的火力杀伤距离增大至 1km,其他参数不变	将舰载探测系统与交战系统的有效工作时段衔接起来
方案 3	舰载探测系统的探测距离增大至原来的 1.5 倍,CIWS 的火力杀伤距离增大至原来的 2 倍,其他参数不变	考察探测跟踪能力和火力拦截能力对舰艇反集群作战能力的影响
方案 4	小型无人机集群的到达速率增大为 6 架/s,其他参数不变	间接考察通信干扰能力和伪装欺骗能力对舰艇反集群作战能力的影响
方案 5	在方案 3 的基础上,将交战系统的杀伤概率都增大至原来的 1.5 倍,其他参数不变	考察精确打击能力对舰艇反集群作战能力的影响

装备通信干扰能力和伪装欺骗能力的提升则间接引起小型无人机到达舰艇探测与火力攻击区的速率增大。同时,仿真中还要考虑小型无人机的数量和飞行速度对集群突防能力的影响。

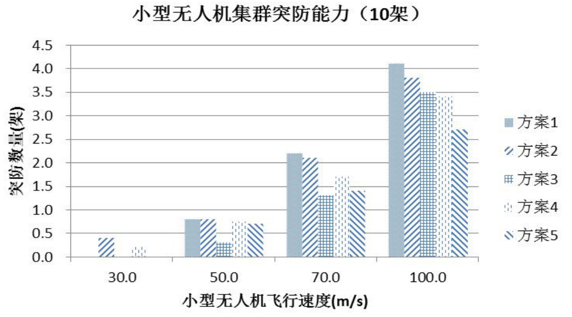

(a)

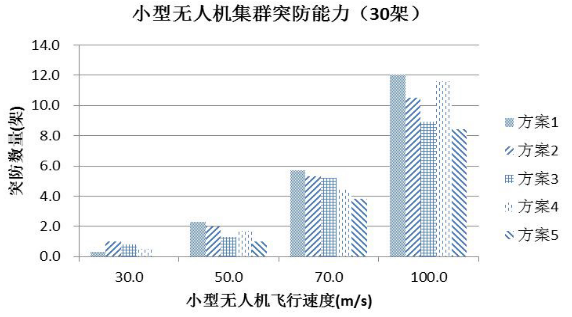

(b)

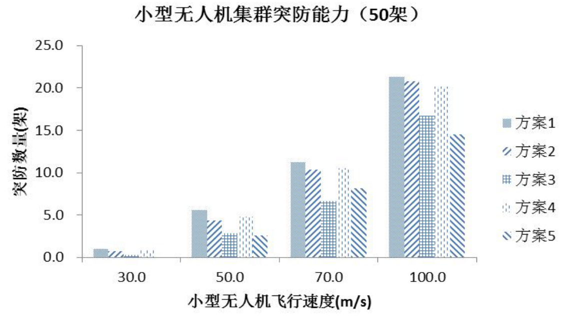

(c)

图 2 小型无人机集群突防单个“宙斯盾”舰的仿真结果

为此,本文以美国最先进的“宙斯盾”舰为例,将其舰载探测系统与交战系统作为对抗小型无人机集群的反制装备,以表 2 提供的装备性能参数作为蓝本[18,19],给出的仿真评估方案见表 3。

5.2 仿真分析

根据给定的仿真参数与评估方案,共模拟水面舰艇反制小型无人机集群过程 6000 次,得到如图 2 所示的仿真结果。

由图 2 可知,方案 3、方案 5 与其他评估方案相比,无论是低速飞行(30m/s)的小规模无人机集群,还是快速飞行(≥70m/s)的大规模无人机集群,都能在一定程度上降低小型无人机集群的突防率,这说明提高反制装备的探测跟踪能力可以尽早发现小型无人机,而提高反制装备的毁伤拦截能力(包括有效杀伤距离和杀伤概率)可以在提早发现小型无人机的前提下有更大的概率毁伤拦截小型无人机。同样,对于方案 2,将舰载探测系统与交战系统的有效工作时段衔接起来也是必要的,这样可避免小型无人机集群在海上防空行动中出现“真空期”,增加反制方毁伤打击小型无人机的次数,从而降低集群成功突防的可能性。在仿真过程中,我们只是通过减小无人机集群的到达速率来间接考察通信干扰能力和伪装欺骗能力对集群突防能力的影响,其效果并不理想,但在实际的作战过程中,对小型无人机集群实施有效的通信干扰和伪装欺骗,一般会使规模较大的无人机集群演变成多个规模较小的无人机集群,大大降低其在数量上的非对称优势,从而减小反制方的防空压力。

值得注意的是,对于快速飞行(≥70m/s)的大规模无人机集群来说,仅仅单纯提高反制装备的探测跟踪能力与毁伤拦截能力是远远不够的,主要是由于在数量上呈现压倒性优势的无人机集群可使反制方的探测系统与武器交战系统瞬间饱和,故需

(C)1994-2020 China Academic Journal Electronic Publishing House. All rights reserved. http://www.cnki.net

要将多个反制装备进行分散配置,建立具备梯次纵深特点的预警探测区和火力拦截区,从而减少探测系统与武器交战系统瞬间饱和状态的出现。

6 结束语

本文以未来海上防空行动面临的新型威胁——小型无人机集群为出发点,从系统组成、指挥控制方式以及典型作战平台的战技术性能探究其在未来海上进攻作战行动中可能出现的 3 种战术运用模式,并剖析了小型无人机集群对我海上行动所产生的不利影响。同时,对水面舰艇的探测系统与交战系统是否具备反制小型无人机集群的装备能力进行了定性定量分析,并从预警探测、通信干扰、伪装欺骗和毁伤拦截四个方面着眼反集群作战装备的能力建设。针对海战场环境与海上兵力运用的特点,从作战指挥体系、预警侦察体系和反制拦截体系构建三个方面寻找符合"体系破击战"指导思想的应对之策。最后,我们以"宙斯盾"舰作为仿真实例,运用离散事件仿真软件对水面舰艇反制小型无人机集群的过程进行了动态建模,探测性能、武器杀伤性能的提升有助于降低小型无人机集群的突防能力。同时,在未来反制装备能力建设中,应加大通信干扰装备和伪装欺骗装备的研制力度,通过合理配置反集群作战资源来提高海上应对小型无人机集群的响应处置能力。目前,我们在仿真中所构建的模型都是粗粒度的概率模型,并未考虑海战场环境、探测盲区、拦截武器射击死区等具体细节。在未来的工作中,我们将考虑上述情况,进一步讨论多舰反制小型无人机集群的能力评估方法,以贴近实战。

参考文献

[1] 赵彦杰.无人机蜂群系统的国外现状与趋势 [EB/OL]. http://mp.weixin.qq.com/s/A3uCXsQ67jqe6aQGbnqa8A,2017.04.23.

[2] 美军郊狼无人机大编队飞行试验蜂群进攻宙斯盾舰 [EB/OL].(2016-07-27) http://mp.weixin.qq.com/s/ZoZ0S4RnLvQMXCqd7gHBEA.

[3] 美国雷神公司"郊狼"无人机即将开展下一阶段蜂群试验 [EB/OL].(2016-08-02) http://mp.weixin.qq.com/s/SX-LXd__SWC7lz8Xnmq2E-g.

[4] ARTHUR H M. Counter-Drone Systems [R/OL]. Center for the Study of the Drone at Bard College,(2018-01-18) [2019-02-07]. https://dronecenter.bard.edu/files/2018/02/CSD-Counter-Drone-Systems-Report.pdf.

[5] 牧野.无人机集群作战技术研究进展及发展建议 [J].远望周刊,2017(1):1-17.

[6] 黄雷.美军小精灵无人机群项目发展现状综述 [J].飞航导弹,2018(7):44-47.

[7] 许瑞明. 无人机集群作战涌现机理及优化思路研究 [J].军事运筹与系统工程,2018,32(2):14-17.

[8] 张巍巍."小精灵"无人机发展研究 [J].飞机设计参考资料,2017(4):35-40.

[9] 李晗,苏京昭,闫咏.智能无人机集群技术概述 [J].科技视界,2017(26):5-7.

[10] 王虎,邓大松.集群式无人机能力分析及其御对策研究 [J].飞航导弹,2017(4):15-20.

[11] 申超,武坤琳,宋怡然.无人机蜂群作战发展重点动态 [J].飞航导弹,2016(11):28-33.

[12] Echodyne In Final Stages of Testing its Metamaterials Based Electronically Scanning Array Radar [EB/OL].(2018) [2019-02-22]. https://www.everythingrf.com/News/details/6939-Echodyne-In-Final-Stages-of-Testing-its-Metamaterials-Based-Electronically-Scanning-Array-Radar.

[13] AUDS Anti-UAV Defence System [EB/OL].(2017) [2019-02-22]. http://www.blighter.com/-products/auds-anti-uav-defence-system.html.

[14] 阎胜利.微型导弹捷联光学制导信息提取方法研究 [J].航天电子对抗,2017(5): 13-15,33.

[15] 汪德武,曹延伟,董靖.国际军控背景下集束弹药技术发展综述 [J].探测与控制学报,2010,32(4):1-6.

[16] GROHE K. Design and development of a counter swarm prototype air vehicle [R]. Monterey,California. Naval Postgraduate School,2017: 14-16.

[17] RYDALCH F D. Missile demonstrator for counter UAV applications [D]. Monterey,California. Naval Postgraduate School,2016: 13-49.

[18] PHAM L V,DICKERSON B,SANDERS J,et al. UAV swarm attack: protection system alternatives for destroyers [R]. Naval Postgraduate School Monterey CA,2012: 100.

[19] MARFO S. UAV Swarm operational risk assessment system [R]. Naval Postgraduate School Monterey CA,2015: 9-15.

(C)1994-2020 China Academic Journal Electronic Publishing House. All rights reserved. http://www.cnki.net
