# Supplementary material for: A game-based approach for designing a collaborative evolution mechanism for unmanned swarms on community networks
Source: Sci Rep. 2022 Nov 7;12:18892. doi: 10.1038/s41598-022-22365-z (PMC9640601; doi:10.1038/s41598-022-22365-z)
Supplement: Supplementary file 1 — Supplementary Information. [file 41598_2022_22365_MOESM1_ESM.zip › Supporting material/Thesis Title & Website Address.docx]

| **S/N** | **Thesis Title** | **Website Address** |
| --- | --- | --- |
| 1 | Cooperative Evolution Mechanism of Unmanned Swarms within the Framework of Public Goods Game | https://doi.org/10.1155/2021/5575815 |
| 2 | An Approach to Coordinated Control of Structured Unmanned Swarms based on Evolutionary Game | https://ieeexplore.ieee.org/document/9275030 |
| 3 | Evolutionary Mechanism of Unmanned Swarms Cooperation based on Evolutionary Game of Multiple Public Goods. (in chinese) | https://kns.cnki.net/KCMS/detail/detail.aspx?dbcode=CJFD&filename=XTYD202012016 |
| 4 | Strategy Dominance Condition of Unmanned Combat Cluster based on Multi-player Public Goods Game. (in chinese) | https://kns.cnki.net/kcms/detail/detail.aspx?dbcode=CJFD&dbname=CJFDLAST2021&filename=XTYD202109024&uniplatform=NZKPT&v=eV6a0WLq41qdyumDytMEml9a8SBoqJ-GqtcDBlci_oJLQ7qxiT2pF8R-e6laW-Ke |
| 5 | A Mechanism for Identifying and Suppressing the Emergent Flocking Behaviors of UAV Swarms. (in chinese) | https://kns.cnki.net/kcms/detail/detail.aspx?dbcode=CJFD&dbname=CJFDLAST2019&filename=DZXU201902017&uniplatform=NZKPT&v=u_snSdORxIMnC2KfWZB2-Qf5aMXckp38HmsHHPSvo6BMHaEr9cEGpw8rUSQoDYAZ |
| 6 | Research on Unmanned Aerial Vehicle Swarms System Resilience. (in chinese) | https://kns.cnki.net/kcms/detail/detail.aspx?dbcode=CJFD&dbname=CJFDLAST2021&filename=XTYD202101020&uniplatform=NZKPT&v=eV6a0WLq41oOdMzP7Z5EXo6Zf-vcUXMELsjJ-LVisf3u81vm4Z9vC6M2yu8lziwi |
| 7 | Research on Countermeasures Equipment Demand and Coping Strategies for Maritime Small UAV Swarms. (in chinese) | https://kns.cnki.net/kcms/detail/detail.aspx?dbcode=CJFD&dbname=CJFDLAST2020&filename=JSYC201904011&uniplatform=NZKPT&v=1LN0DjkxzbFf_dQXDxhLVZdx5xngmVFyet2bEfCN7eiOw-EPhuHNNTRvm6dD0PtY |
